# Synthetic analogs of an *Entamoeba histolytica* glycolipid designed to combat intracellular *Leishmania* infection

**DOI:** 10.1038/s41598-017-09894-8

**Published:** 2017-08-25

**Authors:** Siew Ling Choy, Hannah Bernin, Toshihiko Aiba, Eugenia Bifeld, Sarah Corinna Lender, Melina Mühlenpfordt, Jill Noll, Julia Eick, Claudia Marggraff, Hanno Niss, Nestor González Roldán, Shinji Tanaka, Masato Kitamura, Koichi Fukase, Joachim Clos, Egbert Tannich, Yukari Fujimoto, Hannelore Lotter

**Affiliations:** 1 Department of Molecular Parasitology, Bernhard Nocht Institute for Tropical Medicine, Hamburg, Germany; 2 Department of Chemistry, Faculty of Science and Technology, Keio University, Yokohama, Japan; 3 Graduate School of Science, Osaka University, Toyonaka, Japan; 4 Junior Group of Allergobiochemistry, Research Center Borstel, Leibniz Center for Medicine and Biosciences, Airway Research Center North (ARCN), German Center for Lung Research (DZL), Borstel, Germany; 5 Graduate School of Pharmaceutical Sciences, Nagoya University, Nagoya, Japan

## Abstract

Intracellular pathogens belonging to the genus *Leishmania* have developed effective strategies that enable them to survive within host immune cells. Immunostimulatory compounds that counteract such immunological escape mechanisms represent promising treatment options for diseases. Here, we demonstrate that a lipopeptidephosphoglycan (LPPG) isolated from the membrane of a protozoan parasite, *Entamoeba histolytica* (*Eh*), shows considerable immunostimulatory effects targeted against *Leishmania* (*L*.) *major*, a representative species responsible for cutaneous leishmaniasis (CL). Treatment led to a marked reduction in the number of intracellular *Leishmania* parasites *in vitro*, and ameliorated CL in a mouse model. We next designed and synthesized analogs of the phosphatidylinositol anchors harbored by *Eh*LPPG; two of these analogs reproduced the anti-leishmanial activity of the native compound by inducing production of pro-inflammatory cytokines. The use of such compounds, either alone or as a supportive option, might improve the currently unsatisfactory treatment of CL and other diseases caused by pathogen-manipulated immune responses.

## Introduction

Immunostimulatory molecules are promising candidates on which to base new treatment strategies that target infectious diseases. A specific and complex group of such molecules comprises glycolipids, and includes self-lipids or microbial lipid antigens that mediate CD1d-restricted activation of an innate-like lymphocyte population that expresses an invariant T cell receptor (TCR): natural killer T (iNKT) cells^1–3^. iNKT cells make a marked contribution to the control of several diseases caused by bacteria, protozoa, and fungi^4, 5^. The most potent activator of NKT cells is α-galactosylceramide (αGalCer), a lipid antigen first identified in a marine sponge (or more likely in a bacterium present in the original preparation)^3, 6^. An alternative to direct ligation of the invariant TCR by glycolipid antigens is binding of microbial ligands to toll-like receptors (TLRs) and subsequent production of interleukin (IL)−12 or IL-18 by dendritic cells, which then activate NKT cells^1^.

We recently isolated an immunostimulatory molecule, lipopeptidephosphoglycan (LPPG), from the membrane of the human pathogenic protozoan parasite *Entamoeba (E.) histolytica* (*Eh*). *Eh*LPPG is a specific inducer of interferon (IFN)-γ (and other cytokines) production by human and murine NKT cells^7, 8^. *Eh*LPPG contains a highly acidic polypeptide component, which is rich in Asp, Glu, and phosphoserine residues. This peptide backbone is extensively modified with linear glycan chains bearing the general structure Glcalpha1–6(n)Glcbeta1-6Gal (where n = 2–23)^9^. The molecule harbors two phosphatidylinositol (PI) anchors, *Eh*PIa and *Eh*PIb, which show structural and immunological similarities to αGalCer. The glycerol component of *Eh*PIa is substituted by a single long fatty acid (28:0 or 30:1) at the sn-1 position; *Eh*PIb bears a similar substitution but also has an additional 16:0 fatty acid in the inositol ring^7^.

Cytokine production by NKT cells stimulated by *Eh*LPPG requires expression of CD1d molecules on the surface of antigen presenting cells (APCs), simultaneous TLR signaling via MyD88, and secretion of IL-12. Moreover, treating mice with hepatic amebiasis with *Eh*LPPG reduces abscesses^7^.

Dual activation of innate immune cells by *Eh*LPPG or its PI anchors led us to assume that these compounds might be ideal candidate molecules for targeting pathogens (e.g., protozoan parasites belonging to the genus *Leishmania*) that invade, multiply, and survive in immune cells. This sand fly-transmitted and poverty-related disease occurs in three forms, depending on the *Leishmania* species involved: visceral, mucocutaneous, and cutaneous leishmaniasis (CL). CL is one of the WHO top-listed Neglected Tropical Diseases and is endemic in 88 countries, with 1.0 million cases reported over the last 5 years^10^. In Asia and Africa, CL is mainly caused by *Leishmania* (*L*.) *major* and manifests as ulcerating skin lesions that are at high risk of bacterial superinfection and, when they do heal, leave disfiguring scarring^11^. Depending on the *Leishmania* species and the host, the parasite can escape elimination from its primary host cells (macrophages, monocytes, dendritic cells, and neutrophils) by switching the host’s protective T_H_1 type immune response into a non-protective T_H_2 type of immune response^12^. Protective immune responses are mediated by cytokines such as IFNγ, inflammasome-activated IL-1β, TNFα, reactive oxygen species, and nitric oxide (NO)^12–14^. To date, no really effective therapeutic agents are available; therefore, new drugs are urgently required^15–18^.

Although NKT cells are thought to control *Leishmania* infection^19–21^, their activation by αGalCer or its synthetic derivate KRN7000 yielded controversial results, which seem to be strain-, host-, and organ-dependent. Treatment of *L. donovani-* or *L. major-*infected C57BL/6 mouse models of visceral leishmaniasis (VL) and CL, respectively, led to exacerbation of disease^22, 23^, whereas improvements were noted in BALB/c mouse models of CL^23^. Modification of the fatty acid chains (i.e., length and/or saturation) has a considerable influence on the immunological properties of this strong immune modulator, but no study has yet examined the therapeutic efficacy of these analogs against leishmaniasis^3, 24, 25^. Similar to *E. histolytica*, *Leishmania* parasites possess a glycophosphoconjugate lipophosphoglycan (LPG) moiety within the membrane, which acts as a virulence factor necessary for survival of the parasite in its vertebrate or eukaryotic host. The structure of the GPI anchor in the parasite is slightly different from that in *E. histolytica* in terms of the length and saturation of the fatty acids. The *Leishmania* LPG prevents the lysis of the parasite by the complement system and interferes with the pro-inflammatory immune response by engaging TLR2 and 4 on macrophages^26^.

Here, we conducted both *in vitro* and *in vivo* (a BALB/c mouse model of CL) experiments to examine the efficacy of the moderate immune modulator *Eh*LPPG as a treatment for infections caused by *L*. *major*. Because of the considerable disadvantages associated with the use of natural compounds for therapeutic purposes (reproducibility, cost, time, and the need for sophisticated purification steps), we here synthesized four analogs of the two *Eh*LPPG GPI anchors, *Eh*PIa and *Eh*PIb, and investigated their capacity to stimulate cytokine production by immune cells of both murine and human origin, as well as their ability to ameliorate intracellular infection by *L. major*.

## Results

### Activity of native *Eh*LPPG against *L. major* infection both *in vitro* and *in vivo*

**Figure 1:**
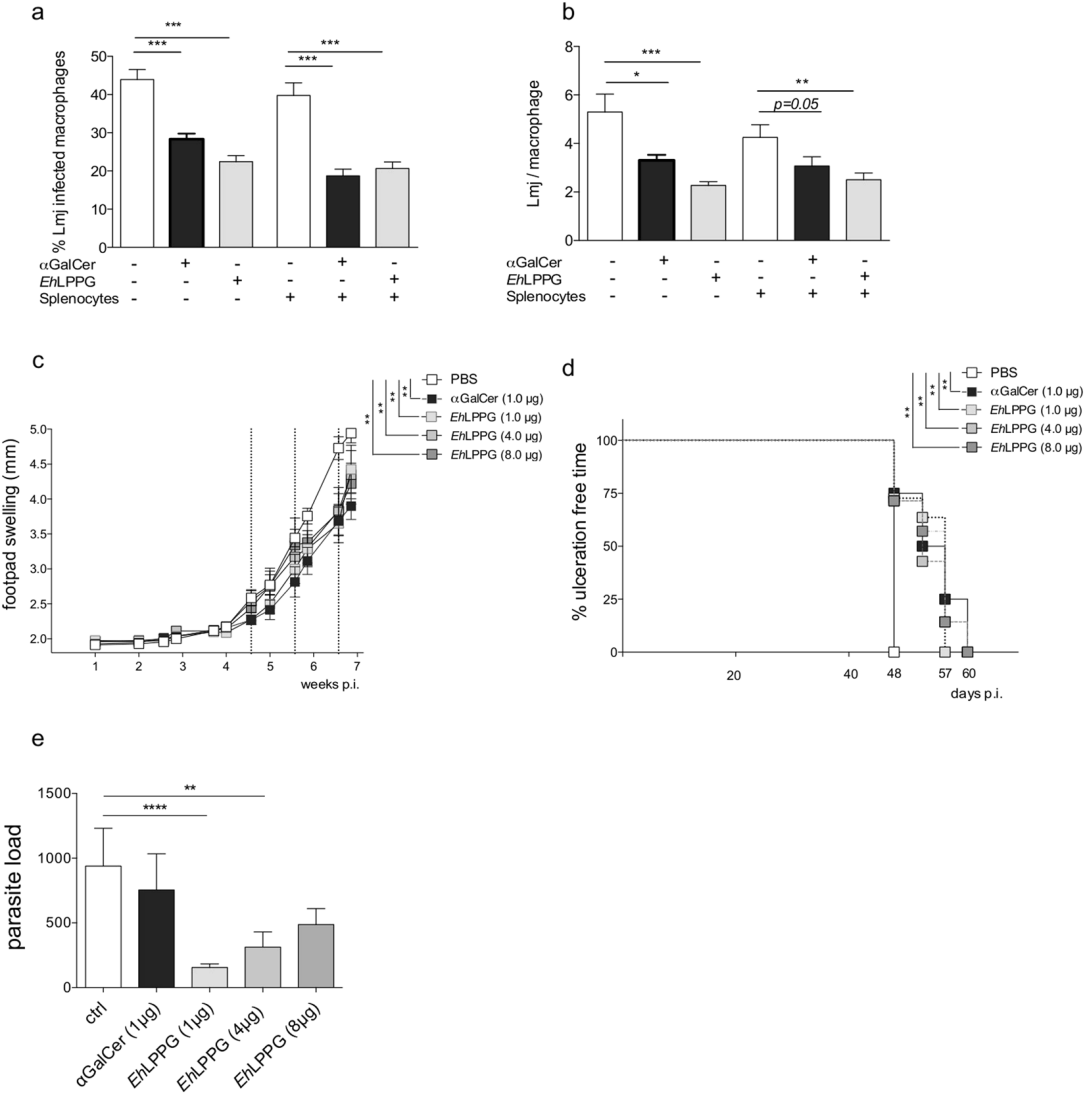
Activity of αGalCer and *Eh*LPPG against *L. major* infection both *in vitro* and *in vivo*. Murine bone marrow-derived macrophages (BMMs; 2 × 10^5^ cells/well) were infected with stationary phase promastigotes parasites (at a MOI of 10 parasites per macrophage) and then treated with αGalCer (1.0 µg/ml) or *Eh*LPPG (8.0 µg/ml) for 48 h in the presence/absence of spleen-derived lymphocytes (4 × 10^5^). Intracellular *L. major* parasites were stained with DAPI, and intracellular infection was examined by immunofluorescence microscopy. (**a**) Percentage of *L. major*-infected macrophages and (**b**) the *Leishmania* amastigote load per macrophage. Female BALB/c mice (group n = 8) were infected with 2 × 10^5^ metacyclic promastigotes and then injected with αGalCer (1.0 µg), *Eh*LPPG (1.0 µg/4.0 µg/8.0 µg), or PBS once per week for three weeks (dashed line). (**c**) Parasite-induced footpad swelling was measured in mm. (**d**) Ulceration-free time in treated animals and (**g**) parasite burden in the draining lymph nodes (determined by qPCR). Results are expressed as the mean ± SEM of 2–3 independent experiments. *p < 0.05; **p < 0.01; and ***p < 0.001 (Mann-Whitney U-test (**a,b,e**) and unpaired Student’s t test (**c,d**)). LMJ, *Leishmania major*.

In the first set of *in vitro* experiments, we asked whether treatment of *L. major*-infected murine bone marrow-derived macrophages (BMMs) with αGalCer or native *Eh*LPPG would reduce the intracellular parasite load. We first examined the percentage of infected macrophages (Fig. 1a) and the *Leishmania* load in each individual macrophage in the presence/absence of spleen-derived lymphocytes (Fig. 1a,b). We found that treatment with both αGalCer (1.0 µg/ml) and *Eh*LPPG (8.0 µg/ml) led to a significant reduction in the percentage of infected macrophages (Control [Ctrl], 43.9 ± 2.7%; αGalCer, 28.3 ± 1.5 [p < 0.0001]; *Eh*LPPG, 22.4 ± 1.6 [p < 0.0001]). Addition of spleen-derived lymphocytes led to a further reduction in the percentage of infected macrophages (Ctrl, 39.8 ± 3.3%; αGalCer, 18.7 ± 1.8 [p < 0.0001]; *Eh*LPPG, 20.6 ± 1.7 [p < 0.0001]) (Fig. 1a). Treatment with αGalCer and *Eh*LPPG also reduced the parasite load per macrophage (Ctrl, 5.3 ± 0.74; αGalCer, 3.3 ± 0.2 [p < 0.043]; *Eh*LPPG, 2.3 ± 0.15 [p < 0.0001]); however, addition of spleen cells had a stronger effect on the parasite/macrophage counts after αGalCer treatment than after *Eh*LPPG treatment (Ctrl, 4.3 ± 0.5; αGalCer, 3.1 ± 0.4 [p = 0.05]; *Eh*LPPG, 2.5 ± 0.3 [p < 0.0018]), supporting the notion that αGalCer is the more potent stimulator of peripheral immune cells (Fig. 1b). Overall, *Eh*LPPG exhibit a similar﻿ *in vitro* anti-leishmanial activity compared to αGalCer. To determine the therapeutic potential of *Eh*LPPG, we subcutaneously (s.c.) injected the footpad of BALB/c mice with 2 × 10^5^ metacyclic promastigotes. Beginning at around 4–5 weeks post-infection (p.i.), when the mice developed typical footpad swelling, the footpad was injected with either αGalCer (1.0 µg), *Eh*LPPG (1.0 µg/4.0 µg/8.0 µg), or PBS (three times at weekly intervals). At around 5 weeks p.i., the degree of footpad swelling in PBS-treated control mice was similar to that in the treated groups (Fig. 1c); however, after the second treatment, we observed less footpad swelling in the treatment groups than in the control group, which became statistically significant after 6 weeks (Day 46 p.i.) (Ctrl, 4.7 ± 0.16 mm; αGalCer, 1.0 µg: 3.7 ± 0.19 mm [p = 0.0029]; *Eh*LPPG, 1.0 µg: 3.7 ± 0.27 mm [p = 0.013]; 4.0 µg: 3.0 ± 0.24 mm [p = 0.02]; 8.0 µg: 3.8 ± 0.34 mm [p = 0.03]). Ethical considerations mandated that the experiments were terminated when ulceration at the site of infection was first observed. The time up until this point, designated as the “ulceration-free time”, was significantly longer in the treatment groups. Lesions in PBS-treated mice ulcerated around Day 48 p.i. By contrast, αGalCer or *Eh*LPPG led to significant prolongation (up to 13 days) of the ulceration-free time (αGalCer (1.0 µg) [p = 0.006]; *Eh*LPPG (1.0 µg) [p = 0.002]; *Eh*LPPG (4.0 µg) [p = 0.004]; *Eh*LPPG (8.0 μg) [p = 0.004]) (Fig. 1d). To assess the effect of treatment on parasite spread, the draining lymph nodes were excised and analyzed by *L. major* actin-specific TaqMan PCR to detect parasites (Fig. 1e). In contrast to αGalCer, treatment with *Eh*LPPG led to a significant reduction in the parasite load (*Eh*LPPG (1.0 µg), 155.2 ± 27.7 [p < 0.0001]; *Eh*LPPG (4.0 µg), 312.6 ± 117.7 [p = 0,001]; *Eh*LPPG (8.0 µg), 487.1 ± 123.7 [not significant; ns]; Ctrl, 939.6 ± 291.4; αGalCer (1.0 µg), 755.1 ± 278.9 [ns]), although it is noteworthy that the parasite load showed an inverse correlation with the dose of *Eh*LPPG.

### Chemical synthesis of the EhLPPG fragments EhPIa and EhPIb

**Figure 2:**
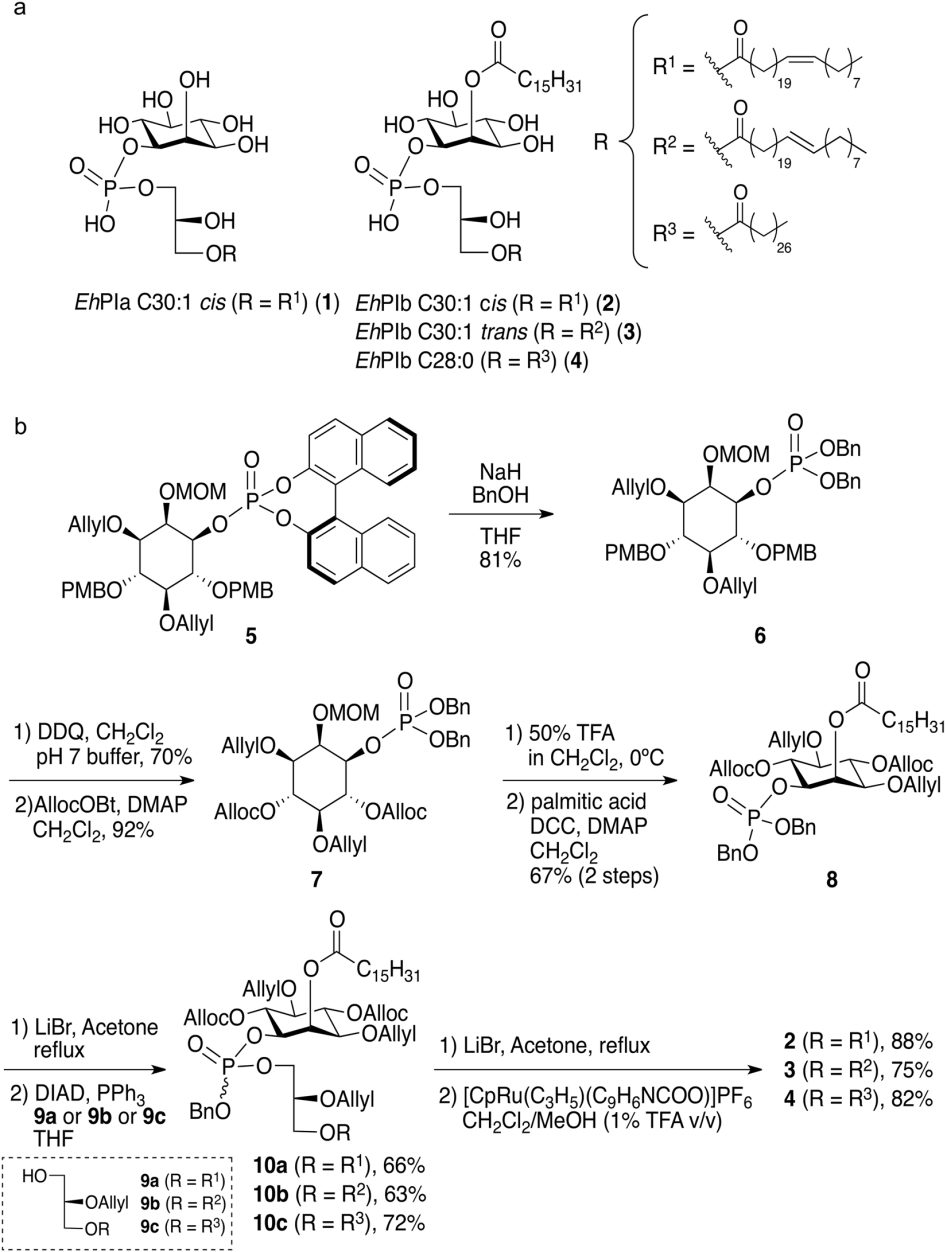
Synthesis of *Eh*LPPG analogs. (**a**) Structure of *Eh*PIa C30:1 *cis* (**1**)^27^, *Eh*PIb C30:1 *cis* (**2**), *Eh*PIb C30:1 *trans* (**3**), and *Eh*PIb C28:0 (**4**). (**b**) Schematic showing synthesis of the *Eh*LPPG analogs.

We identified two isoforms of the GPI anchor harbored by *Eh*LPPG: *Eh*PIa and *Eh*PIb^7^ (Fig. 2a). Each comprise lysophosphatidylglycerol, which has a characteristic long-chain fatty acid of either 28 saturated carbons (C28:0) or 30 mono-unsaturated carbons (C30:1). *Eh*PIb has an additional fatty acid at the 2-position of *myo*-inositol. Therefore, we synthesized an *Eh*PIa analog with a C30:1 *cis* fatty acid structure (*Eh*PIa C30:1 *cis*, **1**) and three *Eh*PIb analogs: *Eh*PIb C30:1 *cis* (**2**), *Eh*PIb C30:1 *trans* (**3**), and *Eh*PIb C28:0 (**4**). The configuration of the glycerol moiety was not clearly determined by spectroscopy^7^; therefore, we synthesized analogs **2**, **3**, and **4**, which have *sn*-glycerol 1-phosphate moieties, as the model structure via **10a**–**10c** using the chiral acyl glycerol **9a**–**9c**.

In combination with newly developed phosphorylation methods used to synthesize *Eh*PIa **1** ^27^, we developed a method for synthesizing inositol phospholipids harboring an unsaturated fatty acid. During synthesis, allyl and allyloxycarbonyl (alloc) groups were used to permanently protect the hydroxyl groups. The allyl and alloc groups could be removed under mild and neutral conditions incorporating transition metal catalysts. Here, we used a specially developed Ruthenium (Ru) catalyst (a cationic CpRu^II^ complex) in combination with quinaldic acid, which was used as a catalytic deprotecting reagent^28^ in the presence of an alcoholic solvent. Commonly used benzyl type protecting groups that can be removed by conventional hydrogenolysis were not applicable to the *Eh*LPPG analogs due to their unsaturated fatty acid structure.

Synthesis of allyl/alloc-protected inositol building blocks was initiated from a mono-phosphorylated inositol intermediate **5** (Fig. 2b)^27^. Transesterification of **5** to a benzyl ester under basic conditions yielded **6** (81% yield). *p*-Methoxybenzyl groups were then converted to alloc groups in a two-step protocol. After removal of the methoxymethyl group under an acidic condition, palmitic acid was used for the coupling reaction to yield **8**. One of the benzyl esters in **8** was then cleaved by LiBr in acetone, and a mono-acylglycerol **9a**, **9b**, or **9c** (harboring C30:1 *cis*, C30:1 *trans*, or C28:0 fatty acid structures, respectively) was introduced to yield **10a**, **10b**, or **10c**, respectively, via the Mitsunobu reaction. After removal of the benzyl group with LiBr, we attempted global deprotection using the Ru complex and CH_2_Cl_2_/MeOH as a solvent system. However, not all alloc groups were removed. This was presumably caused by lithium salt of the phosphate from compound **8**, which acted as a buffer and deprotonated the active form of the Ru complex. Addition of trifluoroacetic acid to the reaction system overcame this problem^29^, and the reaction proceeded smoothly under mild conditions to yield **2**, **3**, and **4** (with yields of 88%, 75%, and 82%, respectively) (see also Supporting information 1).

### The synthetic *Eh*LPPG analogs show only mild cytotoxicity

**Figure 3:**
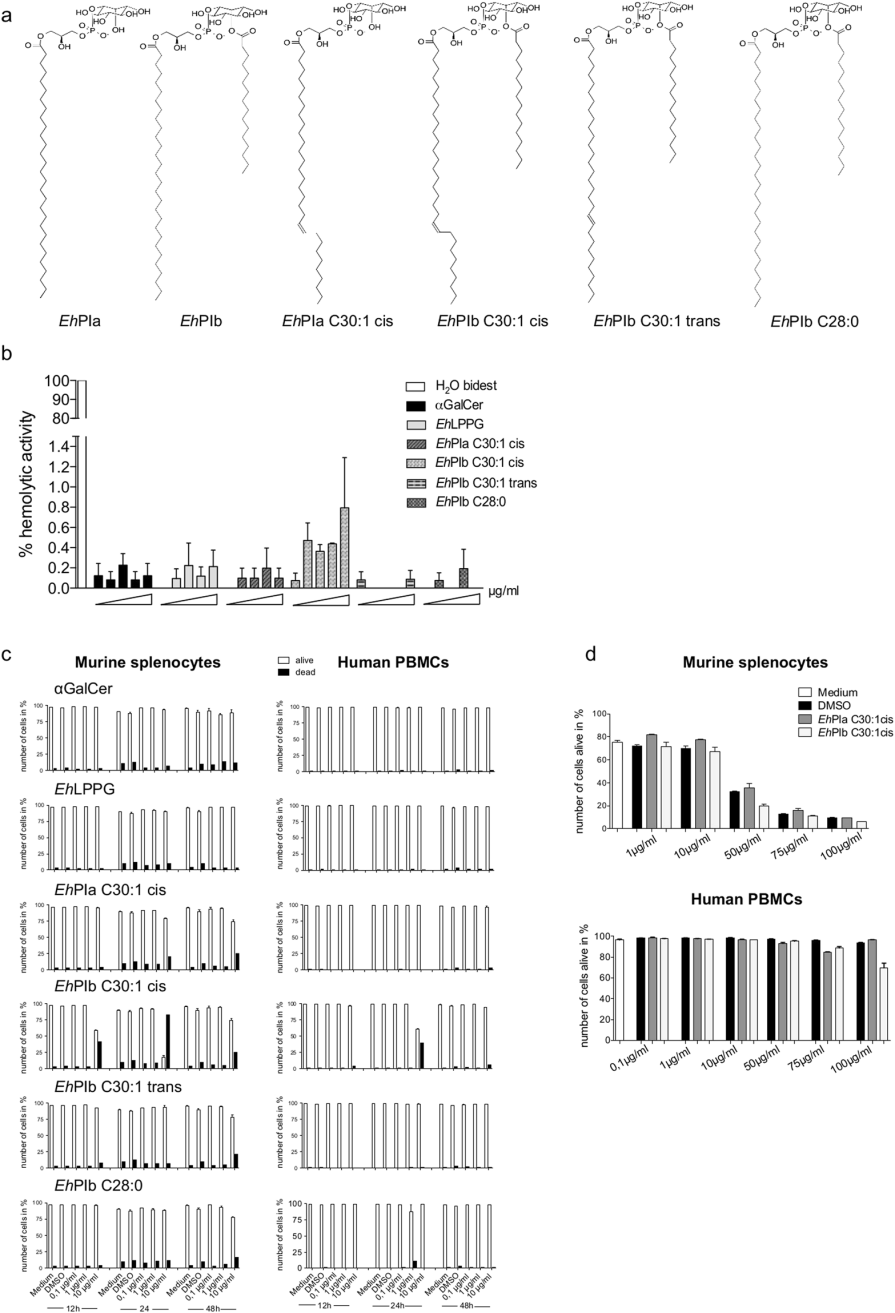
Toxicity of αGalCer, *Eh*LPPG, and the synthetic *Eh*PI analogs *in vitro*. (**a**) Chemical structures of the native *Eh*LPPG GPI anchors (*Eh*PIa and *Eh*PIb) and the synthetic *Eh*PI analogs *Eh*PIa C30:1 cis, *Eh*PIb C30:1 cis, *Eh*PIb C30:1 trans, and *Eh*PIb C28:0. (**b**) Hemolytic activity of αGalCer, *Eh*LPPG, and synthetic *Eh*PI or DMSO (3%) against human red blood cells (RBCs) and (**c**) cytotoxicity against murine splenocytes and human PBMCs after 12, 24, and 48 h of incubation. (**d**) Concentration-dependent cytotoxicity of DMSO, *Eh*PIa C30:1 cis, and *Eh*PIb C30:1 cis against murine splenocytes and human PBMCs. Results are expressed as the mean ± SEM of 2–3 independent experiments.

Due to their long, hydrophobic, single or double fatty acid chains, the synthetic analogs of the *Eh*LPPG PI anchor may be cytotoxic to eukaryotic cells via potential insertion into the lipid bilayer (Fig. 3a). Therefore, we tested the cytotoxicity of the molecules in two assays: a classical cytotoxicity assay, which determined the toxicity of new compounds against erythrocytes, and an assay based on murine or human lymphocytes. Human erythrocytes were incubated with different amounts of the synthetic analogs or water; the latter efficiently lyses erythrocytes within several minutes. Of all compounds tested, only *Eh*PIb C30:1 cis showed (minimal) hemolysis at increasing concentrations (Fig. 3b). Better assessment of cytotoxic properties can be achieved using eukaryotic lymphocytes. Here, we tested the time- and concentration-dependent cytotoxicity of the synthetic analogs against murine splenocytes and human peripheral blood mononuclear cells (PBMCs) by FACS analysis based on live/dead staining (Fig. 3c).

In general, we observed a slight concentration- and time-dependent increase in the percentage of dead murine splenocytes in the presence of the synthetic analogs, which was more pronounced at higher concentrations; this was not the case for αGalCer and *Eh*LPPG. However, *Eh*PIb C30:1 cis appeared to be more cytotoxic than the other synthetic compounds or the native molecule itself. Only *Eh*PIb C30:1 cis (at a concentration of 10 µg/ml for 24 h) was cytotoxic to PBMCs (Fig. 3c), suggesting that it has low potential *in vivo* cytotoxicity. We further investigated the toxicity of *Eh*PIa C30:1 cis and EhPIb C30:1 cis using murine spleen-derived lymphocytes and human PBMCs (Fig. 3d). As seen in Fig. 3c, murine lymphocytes appear more sensitive to the synthetic analogs than human lymphocytes. More specifically, we found that both analogs killed 50% of murine cells when used at a concentration of 50 µg/ml (*Eh*PIa C30:1 cis: log IC_50_, 49.75; *Eh*PIb C30:1 cis: log IC_50_, 49.39; DMSO: log IC_50_, 49.75). However, since the same concentration of DMSO alone exhibited a similar effect, we assumed that the toxicity might be attributed mostly to the solvent. By contrast, human peripheral lymphocytes were not lysed by DMSO or *Eh*PIa C30:1 cis, even at the highest concentrations (100 µg/ml) tested. *Eh*PIb C30:1 cis caused only minor cytotoxicity when used at a concentration of 100 µg/ml for 24 h. The data further suggest that the compounds would have low cytotoxicity *in vivo*.

### Immunostimulatory characteristics of the synthetic *Eh*LPPG analogs

**Figure 4:**
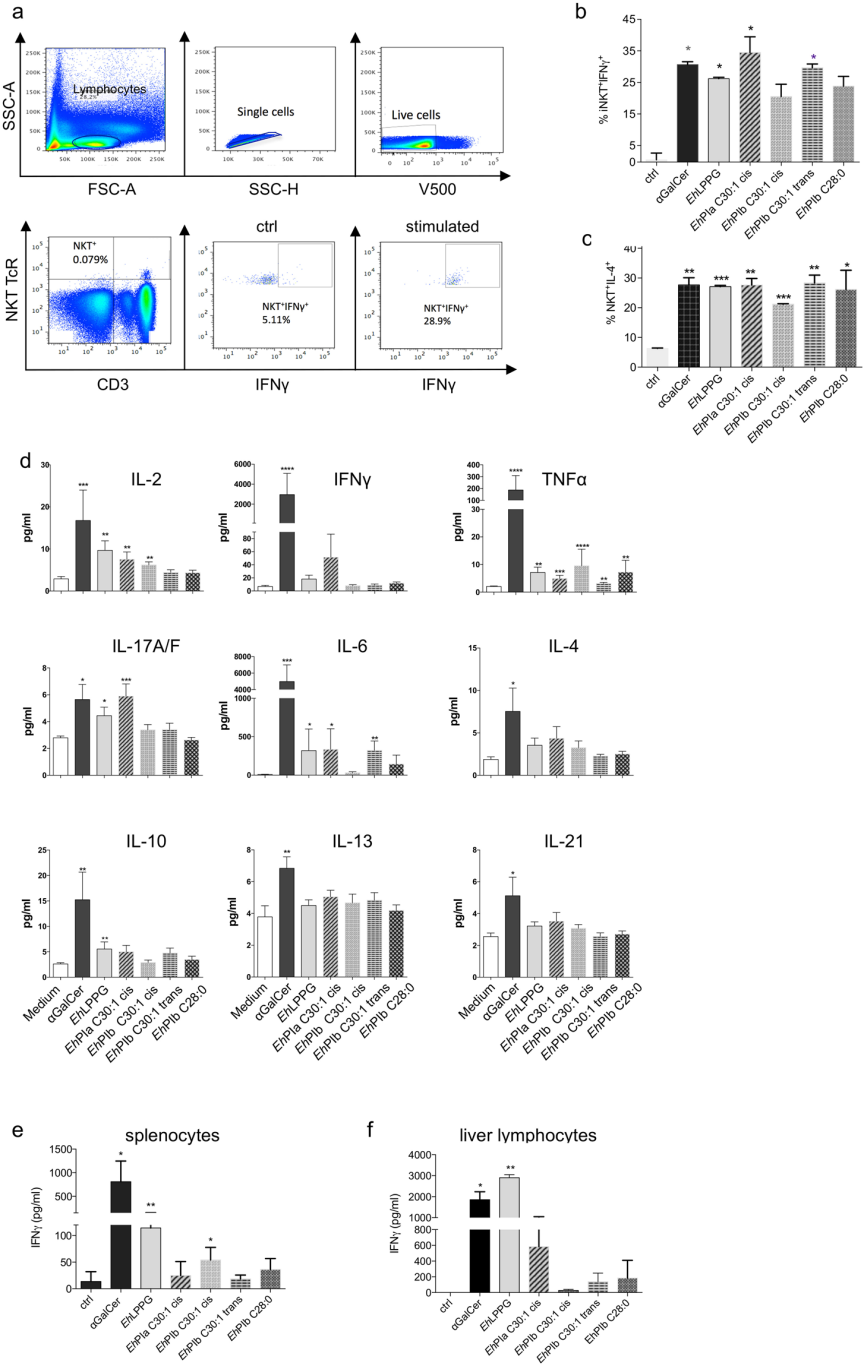
Cytokine profiles of human and murine immune cells in response to αGalCer, *Eh*LPPG, and the synthetic *Eh*PI analogs. (**a**) Gating strategy used for analysis of intracellular IFNγ and IL-4 cytokine production by human peripheral NKT cells. (**b**) Percentage of NKT^+^IFNγ^+^ and (**c**) NKT^+^IL-4^+^ cells present after stimulation of PBMCs with αGalCer, *Eh*LPPG, or synthetic *Eh*PI analogs (0.1 µg/ml). Data from one experiment out of four (all with similar results) are shown. (**d**) Cytokine concentrations in supernatants from human PBMCs (n = 4 donors) stimulated with αGalCer, *Eh*LPPG, or the synthetic *Eh*PI analogs, as determined in a cytometric bead assay (LEGENDplex™, BioLegend). IFNγ concentrations in supernatants from BMMs pulsed with αGalCer, *Eh*LPPG, or the synthetic *Eh*PI analogs and then incubated with murine (**e**) splenocytes and (**f**) liver lymphocytes for 48 h, as determined by ELISA. The following concentrations were used: αGalCer (1.0 µg/ml), *Eh*LPPG (8.0 µg/ml), and synthetic *Eh*PI analogs (1.0 µg/ml). Data are expressed as the mean ± SEM. *p < 0.05; **p < 0.01; and ***p < 0.001 (unpaired Student’s t test (**a–d**) and Mann-Whitney U-test (**e,f**)).

The native molecule *Eh*LPPG was identified due to its immunostimulatory effect on NKT cells and macrophages^7, 8, 30^. To assess whether the synthetic analogs derived from this molecule still show favorable immunostimulatory properties, we investigated and characterized the cytokine profiles elicited by these compounds. The most convenient way to do this is to demonstrate intracellular cytokine production by NKT cells. Therefore, PBMCs from human blood donors were incubated with the synthetic compounds for several hours prior to FACS analysis. After gating on lymphocytes and including single cells and excluding dead cells, *i*NKT cells were identified as CD3 + hNKT-TCR + (Fig. 4a). Figure 4b shows that a percentage of *i*NKT cells from one representative individual showed higher production of IFNγ and IL-4 after stimulation with αGalCer, *Eh*LPPG, or the synthetic analogs than CD28-stimulated Ctrl NKT cells. We observed a significant increase in the percentage of IFNγ + *i*NKT cells following stimulation with αGalCer (30.9 ± 0.65; p = 0.029), *Eh*LPPG (26.4 ± 0.3; p = 0.038), *Eh*PIa C30:1 cis (34.75 ± 3.5; p = 0.032), and *Eh*PIb C30:1 trans (29.9 ± 0.85; p = 0.03). IL-4 was induced by all molecules tested (Fig. 4c). We also analyzed the broader cytokine spectrum in the supernatant of stimulated PBMC cultures using a cytometric bead assay (Fig. 4d). Stimulation with αGalCer led to highly significant induction of pro-and anti-inflammatory cytokines, while stimulation with the native compound (*Eh*LPPG) induced much lower cytokine levels, with greater induction of pro-inflammatory cytokines (i.e., IL-2, TNFα, IL-17, and IL-6) than anti-inflammatory cytokines (i.e., IL-10). Stimulation with *Eh*PIa C30:1 cis induced levels of pro-inflammatory cytokine (IL-2, TNFα, IL-17A/F, and IL-6) production similar to those induced by the native molecule, but did not increase production of anti-inflammatory cytokines. *Eh*PIb C30:1 cis also induced only pro-inflammatory cytokines (IL-2 and TNFα). *Eh*PIb C30:1 trans induced TNFα and IL-6, whereas *Eh*PIb C28:0 induced only TNFα (Fig. 4d). Since we aimed to use the synthetic analogs as therapeutic agents in the mouse model of CL, we examined whether they induced IFNγ production by murine splenocytes or liver lymphocytes (the latter harbor the highest organ-specific concentration of *i*NKT cells in mice). A classical stimulation assay was therefore performed in which BMMs were generated and pulsed with the synthetic analogs for 48 h following addition of lymphocytes (splenocytes or liver lymphocytes). IFNγ levels in the culture supernatant were then measured in an ELISA. We observed significant induction of IFNγ following stimulation of splenocytes (Fig. 4e) and liver lymphocytes (Fig. 4f) with αGalCer, *Eh*LPPG, and *Eh*PIb C30:1 cis. However, we observed only a slight increase in IFNγ production following stimulation of liver lymphocytes with *Eh*PIb C30:1 cis (Fig. 4f). Taken together, these data show that two out of the four synthetic analogs exhibited considerable immunostimulatory activity (*Eh*PIa C30:1 cis on immune cells of human origin, and *Eh*PIb C30:1 cis on immune cells of murine origin).

### Treatment of murine and human macrophages with the EhPI analogs ameliorates *L. major* infection

**Figure 5:**
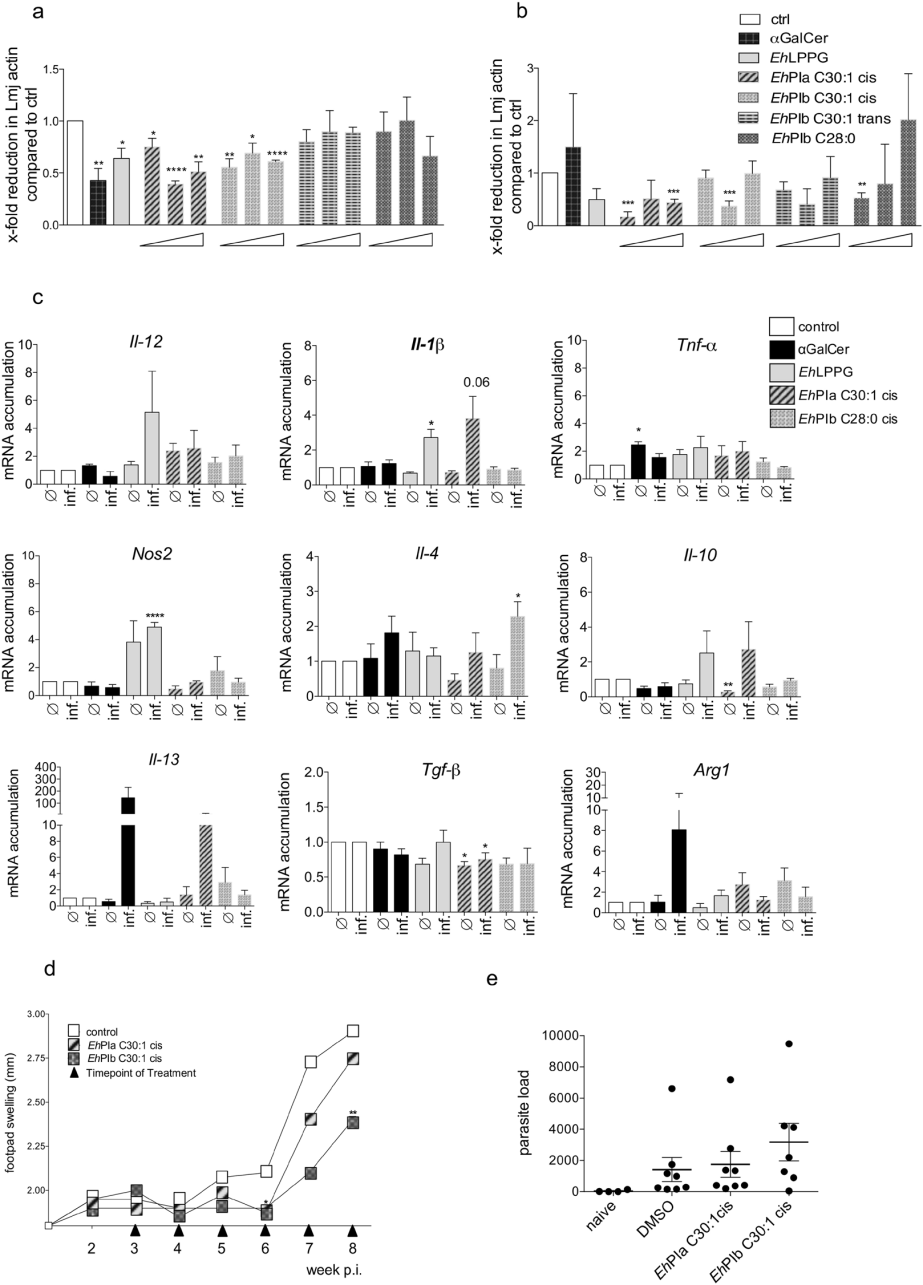
*In vitro* and *in vivo* anti-leishmanial activity of αGalCer, *Eh*LPPG, and the synthetic *Eh*PI analogs. BMMs and human THP1 macrophages were infected with *L. major* at a MOI of 8:1 and then treated with αGalCer (1.0 µg/ml), *Eh*LPPG (8.0 µg/ml), or *Eh*PIa C30:1 cis, *Eh*PIb C30:1 cis, *Eh*PIb C30:1 trans, or *Eh*PIb C28:0 (0.1/1.0/10.0 µg/ml). Genomic DNA was extracted from infected and treated murine and human macrophages and used in a TaqMan probe PCR. Fold reduction in *L. major* actin in (**a**) murine macrophages and (**b**) human macrophages is shown along with controls. Data are expressed as the mean ± SEM of two independent experiments. (**c**) Cytokine (mRNA) expression profile of non-infected (∅) and *L. major*-infected (inf.) murine macrophages treated with αGalCer (1.0 µg/ml), *Eh*LPPG (8.0 µg/ml), or synthetic *Eh*PI analogs *Eh*PIa C30:1 cis and *Eh*PIb C28:0 (0.1 µg/ml), as determined by qPCR. (**d**) Footpad swelling in *L. major*-infected female BALB/c mice treated with DMSO (diluted 1:10/25 µl) or *Eh*PIa or *Eh*PIb C30:1 cis (5 µg in PBS/25 µl). (**e**) Parasite load in the footpads of *L. major*-infected female BALB/c mice treated with DMSO (diluted 1:10/25 µl) or *Eh*PIa or *Eh*PIb C30:1 cis (5 µg in PBS/25 µl), as determined by probe PCR of tissue lysates. Data are expressed as the mean ± SEM of two independent experiments (n = 6–7/experiment). *p < 0.05; **p < 0.01; and ***p < 0.001 (unpaired Student’s t test (**a–k**)).

Since treatment with native *Eh*LPPG showed a parasite reducing effect, we next asked whether synthetic analogs *Eh*PIa C30:1 cis, *Eh*PIb C30:1 cis, *Eh*PIb C30:1 trans, and *Eh*PIb C28:0 had a similar impact on the intracellular *L. major* load. Therefore, we infected murine BMMs and human THP1 macrophages with *L. major* promastigotes (at stationary phase) and measured the intracellular *Leishmania* load using a *Leishmania*-specific TaqMan PCR^31^. We found that treatment of murine macrophages with αGalCer, *Eh*LPPG, *Eh*PIa C30:1 cis, or *Eh*PIb C30:1 cis led to significant inhibition of parasite infection compared with that in infected but untreated cells (αGalCer 1.0 µg/ml: 0.43 ± 0.1 [p = 0.003]; *Eh*LPPG 4.0 µg/ml: 0.64 ± 0.09 [p = 0.011]; *Eh*PIa C30:1 cis, 0.1 µg/ml: 0.75 ± 0.08 [p = 0.025]; *Eh*PIa C30:1 cis, 1.0 µg/ml: 0.39 ± 0.03 [p = 0.0001]; *Eh*PIa C30:1 cis, 10.0 µg/ml: 0.51 ± 0.09 [p = 0.002]; *Eh*PIb C30:1 cis, 0.1 µg/ml: 0.56 ± 0.081 [p = 0.002]; *Eh*PIb C30:1 cis, 1.0 µg/ml: 0.69 ± 0.09 [p = 0.018]; and *Eh*PIb C30:1 cis, 10.0 µg/ml: 0.61 ± 0.013 [p = 0.0001]) (Fig. 5a). However, there was no direct concentration-dependent correlation with the reduction in the parasite load. This may be due to the spontaneous formation of micelles by the amphiphilic fatty acid side chains of the synthetic analogs. We found it interesting that the *L. major* load increased following treatment of human macrophages with αGalCer, but fell following treatment with *Eh*LPPG. Again, *Eh*PIa C30:1 cis and *Eh*PIb C30:1 cis (*Eh*PIa C30:1 cis, 0.1 µg/ml: 0.17 ± 0.095 [p = 0.000]; *Eh*PIa C30:1 cis, 10.0 µg/ml: 0.44 ± 0.06 [p = 0.0002]; *Eh*PIb C30:1 cis, 1.0 µg/ml: 0.65 ± 0.05 [p = 0.0004]; *Eh*PIb C28:0, 0.1 µg/ml: 0.53 ± 0.0.094 [p = 0.003]) were the strongest inhibitors, while *Eh*PIb C30:1 trans and αGalCer had no effect (Fig. 5b).

To further characterize the treatment-specific, immunostimulatory effects of αGalCer, *Eh*LPPG, the most promising analog (*Eh*PIa C30:1 cis), and the least promising analog (*Eh*PIb C28:0), we measured the levels of mRNA encoding infection-relevant protective cytokines (e.g., *Il-12*, *Il-1β*, *Tnfα, Nos2* [iNOS]) and non-protective cytokines (*Il-4*, *Il-10*, *Il-13*, *Tgf-β*, and Arginase 1 [*Arg1*])^12–14^ in non-infected and infected murine macrophages (Fig. 5c). Overall, *Eh*LPPG induced the strongest infection-and treatment-specific increases in mRNA encoding protective cytokines (*Il-1β*, 2.7 ± 0.47 [p = 0.018]; *NOS2*, 4.9 ± 0.3 [p > 0.0001]); however, it had only a moderate effect on mRNA encoding non-protective cytokines (*Il-10*, 2.5 ± 1.2 [ns]). αGalCer increased the accumulation of mRNA encoding protective pro-inflammatory cytokines (*Tnfα*, 1.5 ± 0.29 [p = 0.018]) but induced a strong, although not statistically significant, increase in mRNA encoding non-protective cytokines *Il-4* (1.8 ± 0.47, p = 0.1), *Il-13* (145.9 ± 85.0, p = 0.1), and *Arg1* (8.1 ± 5.3, p = 0.2). Treatment with *Eh*PIa C30:1 cis showed inconsistent results, although there was a marked increase in expression of mRNA encoding *Il-1β* (3.8 ± 1.2, p = 0.06), one of the most relevant cytokines that protects against *Leishmania* parasites. We also observed an increase in expression of mRNA encoding anti-inflammatory cytokines *Il-10* (2.7 ± 1.5, p = 0.2) and *Il-13* (10.2 ± 6.8, p = 0.2). Treatment with *Eh*PIb C28:0, an analog with only minimal immuno-and anti-leishmanial properties, led to an increase in mRNA encoding one non-protective cytokine (*Il-4*, 2.3 ± 0.4; *p* = 0.02), but no protective cytokines. To summarize, *Eh*LPPG and *Eh*PIa C30:1 cis tended to induce expression of mRNA encoding protective pro-inflammatory cytokines (*Il-1β*, *Il-12*, and *Nos2*).

Finally, we examined the therapeutic potential of the most promising analogs (*Eh*PIa C30:1 cis and *Eh*PIb C30:1 cis) in a BALB/c mouse model that is highly susceptible to CL. Mice were infected with *L. major* as described above and treated by local injection of each synthetic analog (5.0 µg) beginning at Week 3 p.i. Both synthetic analogs reduced footpad swelling when compared with the solvent control (DMSO) (Fig. 5d). For *Eh*PIb C30:1 cis, this difference was significant at Weeks 6 and 8 p.i. (p < 0.042* and p < 0.0046, respectively). However, the effect was temporary and the *Leishmania* lesions relapsed at Week 7 (although they remained smaller than those in Ctrl mice). We next determined the parasite load in a tissue lysate prepared from footpads at the time of sacrifice (Week 8 p.i.) but found no significant differences in the *L. major* actin DNA to mouse actin DNA ratio in lysates from treated mice or the DMSO ctrl group (DMSO ctrl: 1420 ± 768.9; *Eh*PIa C30:1 cis: 1755 ± 831.7; *Eh*PIb C30:1 cis: 3699 ± 1288; n = 4, mean ± SE) (Fig. 5e). In addition, we found that treatment with *Eh*PIa C30:1 cis and *Eh*PIb C30:1 cis led to a significant reduction in the levels of anti-inflammatory cytokines IL-4 (*Eh*PIa: p = 0.0002), IL-10 (*Eh*PIa C30:1 cis: p = 0.0001; *Eh*PIb C30:1 cis: p = 0.0001), and IL-13 (*Eh*PIb C30:1 cis: p = 0.01), as well as C-C Chemokine Ligand 2, following treatment with *Eh*PIbC30:1 cis (p = 0.02). This indicates that treatment results in a more favorable protective cytokine response since parasite survival depends on an anti-inflammatory Th2 type immune response (see Suppl. Figure 1). Therefore, we conclude that, although the synthetic analogs reduce disease severity, this reduction is transient in a highly susceptible BALB/c mouse model of the disease.

## Discussion

Intracellular pathogens such as *Leishmania* parasites that invade immune cells are very successful in manipulating the host immune response to circumvent their own clearance. Thus, without the support of the immune system, treatment and elimination of these agents is challenging. Most of the effective therapeutics used to treat VL target the parasite itself; however, they can have considerable side effects. These effects are often not tolerable in cases of the non-lethal and often self-limiting cutaneous form of the disease. There is no effective treatment for CL, although the options explored to date include surgery, curettage, laser, thermo- and cryotherapy, infiltration of the lesion with Pentostam, or the use of ointments containing paromocycin and methlybenzetonium^15, 17, 18, 32^. However, these therapies are either inconvenient or largely ineffective. Therefore, activating or re-activating the immune system using immunostimulatory molecules represents a promising strategy.

Here, we examined the ability of the immunostimulatory glycolipid *Eh*LPPG, as well as newly synthesized analogs derived from its PI anchor, to stimulate a protective cytokine response in immune cells, and their efficacy against *L. major*, in *in vitro* and *in vivo* models.

We previously showed that *Eh*LPPG induces production of IFNγ in a variety of immune cells such as murine spleen and liver lymphocytes, APCs, human and murine iNKT cells, and human PBMCs^8, 30^. In particular, specific stimulation of NKT cells, which shape the immune response after infection^33^, is a promising property of *Eh*LPPG. Besides lymphocytic cells, APCs are activated by *Eh*LPPG. We found that *Eh*LPPG and *Eh*PI activate APCs by binding to TLR2 or TLR6, leading to production of the pro-inflammatory cytokine IL-12 via engagement of MyD88. Simultaneously, *Eh*LPPG is internalized and loaded on CD1d molecules. Both of these pathways are relevant for optimal NKT cell activation^8^. NKT cells also play an important role in immune responses during early stages of *Leishmania* infection; however, these responses appear to be dependent on the *Leishmania* species, mouse strain, and organ. For example, NKT cells contribute to clearance of *L. donovani* from the liver of BALB/c mice^20^ but aggravate VL in C57BL/6 J mice^22^. During a *L. major* infection, the protection afforded by NKT cells seems to be organ-specific in that the cells contribute to parasite elimination from the spleen and skin lesions, but not from the lymph nodes^21^.

The experiments conducted herein show that the native molecule, *Eh*LPPG, and the most potent NKT-agonist and immunomodulator, αGalCer, led to a significant reduction in the parasite burden in *L. major*-infected murine macrophages *in vitro*, an effect that increased slightly upon addition of spleen-derived lymphocytes (Fig. 1a). Also, treatment of a mouse model of CL with *Eh*LPPG had a positive effect on the disease outcome by reducing *Leishmania*-induced footpad swelling, increasing the time to euthanasia due to ulceration of the lesions and reducing parasite dissemination (Fig. 1c–e). This agrees with previous findings showing that the αGalCer analog PBS57 delays lesion growth and reduces lesion size and parasite burden in BALB/c mice^23^. However, the same manuscript described exacerbation of disease pathology in C57BL/6 mice^23^. Most interestingly, another study shows that treatment of C57BL/6 mice with VL with αGalCer aggravates the disease due to increased production of non-protective IL-4 and reduces production of IFNγ^+^CD8^+^ T cells^22^.

However, results obtained using native *Eh*LPPG suggest that a synthetic version, produced under more reproducible conditions than the native compound, might represent a promising therapeutic agent. Since the complexity of the native *Eh*LPPG molecule renders chemical synthesis impossible, we decided to focus on synthesis of the *Eh*PI anchor. Certainly, synthesis of this anchor, which consists of two isoforms with one (*Eh*PIa) or two (*Eh*PIb) fatty acid chains and exhibits immunostimulatory properties, is more feasible^7^. Therefore, we designed and synthesized one analog that is very similar to *Eh*PIa (*Eh*PIa C30:1 cis) and three analogs that are similar to *Eh*PIb (*Eh*PIb C30:1 cis, *Eh*PIb C30:1 trans, and *Eh*PIb C28:0) (Fig. 2; Fig. 3a).

Due to their hydrophobic long fatty acid chains, the structure of the synthetic analogs may induce cytotoxicity via insertion into the lipid bilayer of eukaryotic cell membranes (as is the case for Miltefosine, a drug used to treat VL). Miltefosine also possesses a long single fatty acid chain that, among other things, is responsible for its hemolytic and cytotoxic activity^34, 35^.

Therefore, we examined the hemolytic and cytotoxic activity of the synthetic analogs *in vitro* and found that only *Eh*PIb C30:1 cis showed minor hemolytic activity and almost no cytotoxicity against human peripheral lymphocytes (Fig. 3). We then examined the ability of the synthetic analogs to stimulate immune cells of either human or murine origin. We found that the analogs induced production of IFNγ and IL-4 by human NKT cells (Fig. 4a); therefore, we went on to characterize the cytokine profiles in the supernatants of stimulated human PBMCs. All synthetic analogs induced production of TNFα, while *Eh*PIa induced production of pro-inflammatory cytokines (IL-2, IFNγ, IL-17, or IL-6). Prior analysis of modifications to the length of lipid chains in αGalCer suggested that a single chain analog like *Eh*PIa would show similar or higher activity against human immune cells than a double chain analog like *Eh*PIb, although αGalCer analogs with two lipid chains show higher affinity for murine immune cells^25^. As reported by others, we also found that αGalCer induced strong production of a broad variety of cytokines by PBMCs, indicating the critical potential of this immunostimulator to induce adverse immunological site effects and questioning its use for therapeutic purposes.

However, the synthetic analogs also activated macrophages. Indeed, some analogs reduced the *L. major* load in infected macrophages (Fig. 5a,b). The most immunoactive analogs, *Eh*PIa C30:1 cis and *Eh*PIb C30:1 cis, yielded the most significant reduction in the *L. major* load, irrespective of whether the infected macrophages were murine or human. Strikingly, there was no dose-dependent effect on the *Leishmania* load; we can only speculate that this might be due to rapid and spontaneous formation of micelles, resulting in defective immune activation. However, this might be overcome by incorporating the synthetic analogs into suitable nanocarriers.

Macrophages are not only the main target cells for *Leishmania* parasites, but also the major effector cells required for parasite clearance. Generally, macrophages are activated by two distinct pathways: the classical and alternative pathways. Classical activation is mediated by cytokines such as IFNγ produced by T helper (Th)1 and natural killer cells, resulting in production of inducible nitric oxide synthase (*i*NOS or NOS2) by macrophages. *i*NOS and toxic NO kill intracellular bacteria and parasites, including *Leishmania*. Thus, classically activated macrophages are crucial for parasite elimination. Alternative macrophage activation is induced by IL-4 and IL-13 (Th2 cytokines), which upregulate arginase and polyamine biosynthesis. Because iNOS and arginase compete for the same substrate (L-arginine), upregulation of arginase can reduce *i*NOS activity and production of NO, resulting in parasite survival^12^. We assume that *Eh*LPPG and the effective synthetic *Eh*PI analogs activate and polarize macrophages via the classical pathway, a notion supported by the significantly higher expression of NOS2 mRNA in *L. major*-infected murine macrophages treated with *Eh*LPPG. By contrast, expression of Arg1 mRNA did not differ from that in control cells. The mechanism seems to be a little different for *Eh*LPPG and *Eh*PIa C30:1 cis. *L. major*-infected murine macrophages treated with *Eh*LPPG showed significantly higher expression of NOS2, IL-1β, and IL-12 mRNA, while infected macrophages treated with *Eh*PIa C30:1 cis showed higher expression of only IL-1β and IL-12 mRNA. Thus, *Eh*LPPG can directly stimulate NOS2 upregulation, possible via its sugar residues and greater ability to activate TLR signaling. Finally, the most immunoactive synthetic analogs, *Eh*PIa C30:1 cis and *Eh*PIb C30:1 cis, reduced the size of *Leishmania* lesions in a BALB/c mouse model of CL (Fig. 5). Although only transient, the reductions induced by *Eh*PIb C30:1 cis were significant. Further studies should examine *in vivo* effects using, for example, a less susceptible mouse model, synthetic analogs that are better protected from degradation, and combination therapy with anti-parasitic drugs.

## Methods

### Synthesis of *Eh*LPPG analogs

The *Eh*LPPG analogs *Eh*PIa C30:1 *cis* (**1**)^27^, *Eh*PIb C30:1 *cis* (**2**), *Eh*PIb C30:1 *trans* (**3**), and *Eh*PIb C28:0 (**4**) were chemically synthesized from compound **5** (Fig. 2a)^27^. The detailed synthetic procedures and spectroscopic data are provided in the Supporting information.

### Mice

C57BL/6 J and BALB/c mice were bred and kept under specific pathogen-free conditions in the animal facility at the Bernhard Nocht Institute for Tropical Medicine, Hamburg, Germany.

### Ethics statement

All animal experiments were approved by the review board of the State of Hamburg, Germany (Acquisition No: 46/13; 133/13) and conducted in accordance with institutional and animal research adhering to the NHI Guidelines for the care and use of laboratory animals (ARRIVE guidelines).

All experiments with human samples were performed in accordance with relevant guidelines and regulations. The experimental protocols were approved by the Bernhard-Nocht-Institute for Tropical Medicine and the medical council of Hamburg. All donors provided informed consent.

### Culture of *L. major* parasites

Promastigote *L. major* ASKH5 were grown at 25 °C in modified Medium 199 (Sigma-Aldrich) supplemented with 20% heat inactivated fetal calf serum (FCS), 40 nM HEPES, pH 7.4, 0.2% NaHCO_3_, 100 µM adenine, 1.2 µg/ml 6-biopterin, 10 µg/ml haem, 20 µg/ml gentamicin, and 2 mm L-glutamine (pH 7.0). For experiments with murine and human macrophages and for the mouse infection experiments, parasites were allowed to grow to stationary phase. Promastigotes were counted using a CASY cell counter (Roche).

### *In vitro* infection of murine and human (THP1) macrophages

Murine BMMs were isolated from the tibiae and femurs of 6–10-week-old female C57BL/6 J mice and cultured for 10 days in Iscove’s Modified Dulbecco’s Medium (IMDM, Sigma) supplemented with 10% heat inactivated FCS (Sigma), 5% horse serum (Sigma), and 30% L929 supernatant (modified from Racoosin, 1989 #31). For infection, BMMs were harvested, washed, and seeded into the wells of an 8-well chamber slide (Nunc®) at a density of 2 × 10^5^ cells/well, or into 24-well plates (Sarstedt) at a density of 1.5 × 10^6^ cells/well. The macrophages were incubated for 72 h at 37 °C/5% CO_2_ to allow adhesion. Adherent BMMs were infected with promastigotes (at stationary phase)^36^ at a multiplicity of infection (MOI) of 8 or 10 parasites per macrophage. After 4 h of incubation at 37 °C in IMDM, non-phagocytosed parasites were removed by multiple washes with warm PBS, and BMMs in 8-well chamber slides were treated with 4.0 µg/ml αGalCer or 8.0 µg/ml *Eh*LPPG for 48 h in the presence/absence of freshly isolated spleen lymphocytes (4 × 10^5^). BMMs in 24-well plates were treated with 4.0 µg/ml αGalCer, 8.0 µg/ml *Eh*LPPG, or 0.1, 1.0, or 10 µg/ml *Eh*PIa C30:1 cis, *Eh*PIb C30:1 cis, *Eh*PIb C30:1 trans, and *Eh*PIb C28:0 for 48 h. After incubation, the supernatants were removed. In the case of the 8-well chamber slides, cells were washed twice and fixed in ice-cold methanol. Intracellular parasites were quantified by nuclear staining with DAPI (1.25 µg/ml, Sigma) followed by epi-fluorescence microscopy (Leica) (counting 1000 macrophages/well [in triplicate] per sample). BMMs in 24-well plates were washed twice, and the cells were collected and subjected to genomic DNA (gDNA) isolation (QIAmp gDNA Kit, QIAGEN) or RNA isolation (InviTrap® Spin Cell RNA Mini Kit, Stratec Molecular). gDNA was then used to determine the parasite burden in a probe PCR designed to quantify *Leishmania* actin versus mouse actin. RNA was used in a quantitative PCR (qPCR) to determine the cytokine profiles of infected and treated BMMs. For the human *in vitro* infection assays, human THP1 cells (ATCC®TIB-202) were cultured at 37 °C/5% CO_2_ in RPMI-1640 containing HEPES (RPMI; Sigma) supplemented with 10% inactivated FCS, 1% L-glutamine (PAN), and 1% Pen/Strep (Biofroxx). For infection, THP1 cells were harvested, washed, and seeded into 24-well plates (Sarstedt) at a density of 6 × 10^5^ cells/well. Differentiation of THP1 cells into macrophages was induced by addition of 50 ng/ml PMA (Sigma). THP1 cells were incubated for 72 h at 37 °C/5% CO_2_ to allow differentiation and adhesion. Differentiated THP1 cells were infected with stationary phase promastigotes at a MOI of eight parasites per macrophage. After 4 h of incubation at 37 °C in RPMI, non-phagocytosed parasites were removed by multiple washing steps with warm PBS and THP1 cells were treated with 4.0 µg/ml αGalCer, 8.0 µg/ml *Eh*LPPG, or 0.1, 1.0, or 10 µg/ml *Eh*PIa C30:1 cis, *Eh*PIb C30:1 cis, *Eh*PIb C30:1 trans, or *Eh*PIb C28:0 for 48 h. After incubation, the supernatants were removed. The cells were washed twice, collected, and subjected to gDNA isolation. gDNA was used to determine the parasite burden in a probe PCR designed to quantify *Leishmania* actin versus human actin.

### Determination of cytokine production by human NKT cells and human PBMCs and cytokine levels in tissue lysates

PBMCs were isolated from human blood donors by density gradient centrifugation and stimulated for 15 h with αGalCer (1.0 µg/ml), *Eh*LPPG (8.0 µg/ml), *Eh*PIa C30:1 cis (1.0 µg/ml), *Eh*PIb C30:1 cis (1.0 µg/ml), *Eh*PIb C30:1 trans (1.0 µg/ml), or *Eh*PIb C28:0 (1.0 µg/ml) plus anti-CD28 (3.0 µg/ml) in X-VIVO™ supplemented with 1% Pen/Strep (Lonza). Intracellular cytokines were measured following addition of Brefeldin A (10 µg/ml; 16 h) and staining for iNKT cell-specific surface markers (anti-CD3-PerCP and anti-TCRVα24-Jα18-APC; BioLegend).

Fixation and permeabilization steps (in Perm/Wash solution; 1:10; BD) were followed by staining with anti-IFNγ-PE/Cy7, anti-IL4-PE, and isotype control antibodies (BioLegend). Data were acquired using a BD LSRII flow cytometer and analyzed with FlowJo × 10.07 (Treestar). Tissue lysates were prepared from the footpads of infected and treated BALB/c mice. Briefly, footpads were sectioned and transferred directly to PBS buffer (200 µl) containing a protease inhibitor (Protease Inhibitor Tablets, SIGMAFAST). After adding the same volume of ceramic beads (diameter, 2 mm; Roth), the solution was minced using a tissue lyser (LT, QIAGEN), and centrifuged for 10 min at 13.000 × g at 4 °C. The cytokine profile in the tissue or culture supernatants (IL-2, IL-4, IL-5, IL-6, IL-9, IL-10, IL-17A, IL-17F, IL-21, IL-22, IFNγ, and TNFα) was assayed as described above (except in cases of Brefeldin A treatment) using the multi-LEGENDplex™ analyte flow assay kit (Human Th Cytokine Panel, Cat. No. 740001, BioLegend) according to the manufacturer’s suggestions.

### Measurement of cytokine production in stimulation assays based on spleen or liver lymphocytes

Bone marrow-derived dendritic cells were generated from BMMs by addition of GMCSF; the cells were then used as APCs^37^. Spleen cells were collected from perfused mouse spleen and subjected to erythrolysis. Perfused mouse livers were passed through a mesh filter and lymphocytes were purified by gradient centrifugation as described previously^7^. APCs were pulsed with αGalCer (1.0 µg/ml), *Eh*LPPG (8.0 µg/ml), *Eh*PIa C30:1 cis (1.0 µg/ml), *Eh*PIb C30:1 cis (1.0 µg/ml), *Eh*PIb C30:1 trans (1.0 µg/ml), or *Eh*PIb 28:0 (1.0 µg/ml) for 3 h in the presence/absence of 5 × 10^5^ splenocytes or liver lymphocytes. Cells were then incubated for 48 h, and IFNγ in the supernatants was measured in an ELISA Reader (R&D Systems).

### BALB/c mouse model of CL

The right rear footpads of female BALB/c mice (10–14 weeks old) were injected s.c. with 2 × 10^5^ *L. major* in 25 µl of PBS. Three days prior to infection, parasites were grown in modified Medium 199 until stationary phase was reached. Parasites were then washed twice and resuspended in pre-chilled PBS at pH 7.0. Footpad size was measured twice a week using a caliper. When footpad swelling developed, mice were treated once a week with αGalCer (1.4 µg), purified *Eh*LPPG (1, 4, or 8 µg) (Experiment in Fig. 1c), or *Eh*PIa C30:1 cis (5 µg), *Eh*PIb C30:1 cis (5 µg), or DMSO ctrl (20%) in 25 µl of PBS (Experiment in Fig. 5d) at the indicated intervals. Mice were sacrificed before lesion ulceration to prevent superinfection of the lesions. The draining lymph nodes were collected from infected and non-infected legs, and gDNA was isolated to determine the parasite burden in a probe PCR designed to detect *Leishmania* actin.

### Probe PCRs

Total gDNA was isolated from BMMs or human THP1 cells using the QIAamp® DNA Mini Kit (QIAGEN). Probe PCR was performed using KAPA PROBE FAST Universal qPCR Mastermix (Peqlab). The parasite burden was calculated using the 2^−ΔΔCt^ method^38^ and normalized to that in the infected control. The following primers (5′–3′) were used: LeishAcF2 CAGAACCGTGAGAAGATG; LeishAcR ACAGCCTGAATACCAATG; LeishAcProbe FAM-ATTCAATGTGCCGTCGCTGT-BHQ-1; MouseBetaAcF CTGGAGAAGAGCTATGAG; MoBetaACR2 CTTACCCAAGAAGGAAGGCTG; MouseBetaAcProbe Cy5-CATCACTATTGGCAACGAGCGG-BHQ-3; human β-actin fwd CCCATCTACGAGGGGTATG; human β-actin rev TCGGTGAGGATCTTCATG; and human β-actin probe Cy5–CCTGGCTGGCCGGGACCTGAC–BHQ3.

### Detection of cytokine mRNA

Total cellular RNA was isolated using the InviTrap® SpinCell RNA Mini Kit (Stratec molecular), and cDNA synthesis was accomplished using a Maxima® First Strand cDNA Synthesis Kit prior to RT-qPCR (Thermo Scientific). qPCR was performed using Maxima® SYBR Green qPCR Master Mix (Thermo Scientific). The amount of mRNA was calculated using the 2^−ΔΔCt^ method^38^ and normalized to the house keeping gene ribosomal protein S9 (RPS9). The following primers (5′–3′) were used to investigate the cytokine profile in macrophages: Arginase1: mARG1 for, AACACTCCCCTGACAACCAG and mARG1 rev, CCAGCAGGTAGCTGAAGGTC; IFNγ: mIFNγ for, GATGCATTCATGAGTATTGCCAAGT and mIFNγ_rev, GTGGACCACTCGGATGAGCTC; IL-1β: mIL-1b for, GGAGAACCAAGCAACGACAAAATA and mIL-1b rev, TGGGGAACTCTGCAGACTCAAAC; IL-4: mIL-4_s, CCAAGGTGCTTCGCATATTT and mIL-4_as, ATCGAAAAGCCCGAAAGAGT; IL-10: mIL-10_s, CCAAGCCTTATCGGAAATGA, and mIL-10_as, TCTCACCCAGGGAATTCAAA; IL-12: mIL-12p35_s, AGGTGGCACAGCTACCTCAG and mIL-12p35_as, GACGTCTTCGCCCCTTAAC; IL-13: mIL-13 for, ATCTACAGGACCCAGAGGATATTGC and mIL-13 rev, CTGATGTGAGAAAGGAAAATGAGTCC; Nos2/iNOS: iNOS_for, TGGTGGTGACAAGCACATTT and iNOS_rev, AAGGCCAAACACAGCATACC; MPO: mMPO for, CCATGGTCCAGATCATCACA and mMPO rev, GCCGGTACTGATTGTTCAGG; TGFβ: TGFb_for, TGGAGCAACATGTGGAACTC and TGFb_rev, CGTCAAAAGACAGCCACTCA; TNFα: TNFa_for, AGTTCCCAAATGGCCTCCCTCTCA and TNFa_rev, GTGGTTTGCTACGACGTGGGCT.

### Hemolytic activity

αGalCer, *Eh*LPPG, *Eh*PIa C30:1 cis, *Eh*PIb C30:1 cis, *Eh*PIb C30:1 trans, and *Eh*PIb C28:0 were diluted in PBS and added to 96-well plates at concentrations ranging from 0.1 to 20 µg/ml. An equal volume of red blood cells (RBCs) obtained from EDTA-preserved peripheral blood from a healthy donor was then added. After incubation for 1 h at 37 °C, RBCs were centrifuged at 800 × g at room temperature for 10 min. The absorbance of the supernatant was then measured at 530 nm in an ELISA counter (MRX^e^, Dynex, Magellan Bioscience) with the reference filter set at 630 nm. The percentage hemolytic activity in the presence of each stimulant was estimated as follows: (A − A_0_/A_max_ − A_0_) × 100, where A_0_ represents the background hemolysis obtained by incubation of RBCs with PBS and A_max_ represents 100% hemolysis achieved upon incubation of RBCs in distilled water.

### Cytotoxicity assay using murine splenocytes and human PBMCs

To examine the cytotoxicity of the analogs against murine splenocytes and human PBMCs, murine spleen cells were collected from perfused mouse spleens and subjected to erythrolysis as described previously^7^. Human PBMCs were isolated by density gradient centrifugation, and 1 × 10^6^ splenocytes or human PBMCs were added to 96-well plates prior to stimulation with 0.1, 1.0, and 10.0 µg/ml αGalCer, *Eh*LPPG, *Eh*PIa C30:1 cis, *Eh*PIb C30:1 cis, *Eh*PIb C30:1 trans, or *Eh*PIb C28:0 for 12, 24, or 48 h. Cells were then harvested and stained by live dead staining (Zombie UV™ Fixable Viability Kit, BioLegend) according to the manufacturer’s instructions. Data were acquired in a BD LSRII flow cytometer.

### Statistical analysis

The percentage of infected macrophages, the parasite burden, and footpad size of treated and control samples and mice were compared using an unpaired Student’s t test. Ulceration-free time in control and treated mice was compared using the Mantel-Cox test. Cytokine expression by untreated and treated macrophages, infected macrophages, and infected/treated macrophages was compared using an unpaired Student’s t test. The percentage of IFNγ- and IL-4-producing NKT cells and IFNγ production by murine splenocytes and liver lymphocytes in the presence/absence of test compounds was compared using the Mann-Whitney U-test. Differences were considered significant at the following p-values: *p < 0.05; **p < 0.005; and ***p < 0.0005.

## Electronic supplementary material

Supplementary Information
