# Supplementary material for: Synthetic analogs of an Entamoeba histolytica glycolipid designed to combat intracellular Leishmania infection
Source: Sci Rep. 2017 Aug 25;7:9472. doi: 10.1038/s41598-017-09894-8 (PMC5572710; doi:10.1038/s41598-017-09894-8)
Supplement: Supplementary file 1 — Supplementary Information [file 41598_2017_9894_MOESM1_ESM.pdf]

Synthetic analogs of an *Entamoeba histolytica* glycolipid designed to combat intracellular *Leishmania* infection

Siew Ling Choy, Hannah Bernin, Toshihiko Aiba, Eugenia Bifeld, Sarah Corinna Lender,

Melina Mühlenpfordt, Jill Noll, Julia Eick, Claudia Marggraff, Hanno Niss,

Nestor González Roldán, Shinji Tanaka, Masato Kitamura, Koichi Fukase,

Joachim Clos, Egbert Tannich, Yukari Fujimoto, and Hannelore Lotter

S1. Chemical synthesis of *Eh*PIb analogs.

This section describes the detailed synthesis (all steps, including the introduction of chemical modifications in *Eh*PIb C30:1 cis, *Eh*PIb C30:1 trans, and *Eh*PIb C28:0).

General procedure: Nuclear magnetic resonance (^{1}H NMR, ^{13}C NMR, ^{31}P NMR) spectra were measured at 25 $^{\circ}$C in an indicated solvent with JEOL ECA 500 or ECX 400 or Agilent INOVA 600 and analyzed Delta 5.0.3 (JEOL). The proton chemical shifts in $CDCl_3$ are reported in parts per million (δ) from trimethylsilane as an internal standard and coupling constants are in Heltz (Hz). The chemical shifts in other solvent are reported in ppm from the residual proton signal of solvent. The chemical shits for ^{13}C NMR are reported in ppm from the internal solvent signal ($CDCl_3$, δ 77.0). High-resolution mass spectra (HRMS) of synthetic compounds were obtained on an electron spray ionization quadrupole time of flight (ESI-QTOF) mass spectrometer (micrOTOF-QII-HC; BRUKER). Analytical thin layer chromatography (TLC) was performed on Silica gel 60 F_{254} Plates (Merck, 0.25 mm thickness). Preparative TLC separations were performed on PLC Silica gel 60 F_{254} Plates (Merck, 0.5 or 1.0 mm thickness). Silica gel column chromatography was performed using Silica gel 60 (Merck, 0.040 – 0.063 mm) or Silica gel 60 N [spherical neutral (Kanto Chemical Co., 40 – 50 μm)] at medium pressure (2 – 4 kgcm^{-2}) using indicated solvent systems. Reagents were purchased from commercial supplier (TCI, nacalai tesque, Wako pure chemical industry, Ltd., Kanto Chemical) and were used without further purification. Unless otherwise noted, Non-aqueous reactions were carried out under argon atmosphere. Anhydrous dichloromethane was prepared by distillation from calcium hydrate. Anhydrous tetrahydrofuran was prepared by distillation from Na. Anhydrous *N, N*-dimethylformamide, methanol, Toluene were purchased from Wako pure chemical industry, Ltd.

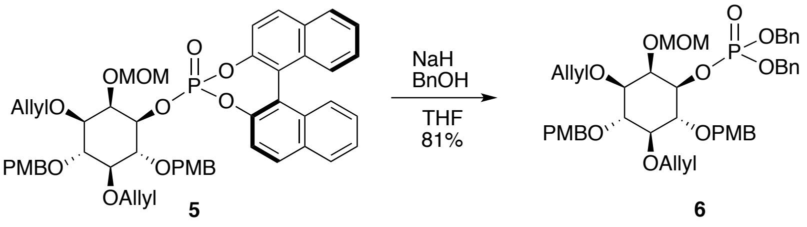

Synthesis of 3,5-di-*O*-allyloxy-4,6-di-*O*-*p*-methoxybenzyl-2-methoxymethyl-D-*myo*-inositol-1-dibenzylphosphate (6)

To a suspension of NaH (60% oil dispersion) (100 mg, 2.52 mmol) in THF (16.8 mL) at 0 °C was added benzyl alcohol (288 μL, 2.77 mmol). After stirring for 30 min, the solution of **5** (734 mg, 0.84 mmol) in THF (6 mL) was added to the reaction at 0 °C. The reaction mixture was warmed up to room temperature, and stirred for 1.5 h. The reaction was cooled to 0 °C, and then quenched with 10% aqueous citric acid. The mixture was diluted with ethyl acetate. The organic solution was washed with saturated aqueous $NaHCO_3$ and brine, dried over anhydrous Na_2SO_4, and concentrated under reduced pressure. The crude mixture was purified with column chromatography (toluene/ethyl acetate = 5/1) to afford **6** (548 mg, 81% yield) as a color less oil. ^{1}H NMR (500 MHz, $CDCl_3$) δ 7.20-7.34 (m, 14H), 6.83-6.87 (m, 2H), 6.75-6.77 (m, 2H), 5.84-5.99 (m, 2H), 5.24-5.30 (m, 2H), 5.11-5.17 (m, 2H), 4.92-5.05 (m, 4H), 4.76-4.80 (m, 4H), 4.66-4.71 (m, 2H), 4.32 (dddt, 2H, J = 28.1, 12.4, 5.5, 1.4 Hz), 4.26 (t, 1H, J = 2.4 Hz), 4.05-4.14 (m, 2H), 3.98 (ddt, 1H, J = 12.6, 5.3, 1.5 Hz), 3.90 (t, 1H, J = 9.6 Hz), 3.83 (t, 1H, J = 9.6 Hz), 3.79 (s, 3H), 3.74 (s, 3H), 3.40 (s, 3H), 3.25 (t, 1H, J = 9.2 Hz), 3.16 (dd, 1H, J = 9.9, 2.4 Hz); ^{13}C NMR (125 MHz, $CDCl_3$) δ 154.36, 134.31, 133.92, 131.61, 131.46, 128.68, 128.64, 128.00, 127.94, 119.08, 119.03, 117.34, 117.16, 97.49, 78.21, 77.36, 77.10, 76.85, 76.70, 76.32, 75.39, 75.35, 73.70, 72.68, 71.05, 69.62, 69.56, 68.77, 68.72, 56.01; HRMS (ESI-QTOF) calculated for $C_{44}H_{53}NaO_{12}P$ $[M+Na]^+$ 827.3167, found 827.3189

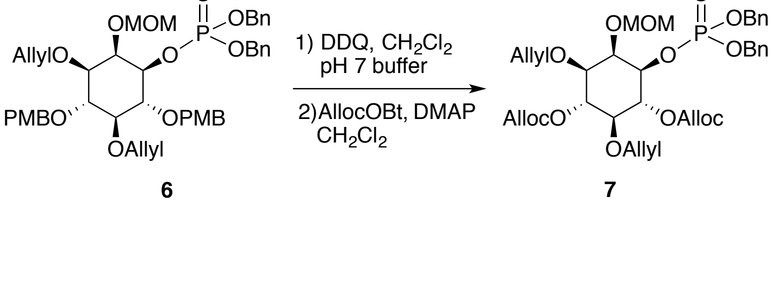

Synthesis of 3,5-di-*O*-allyloxy-2-methoxymethyl-D-*myo*-inositol-1-dibenzylphosphate (7)

To a stirred solution of **6** (124 mg, 0.15 mmol) in CH_2Cl_2 (6.8 mL) and pH 7 buffer (760 μL) was added DDQ (140 mg, 0.62 mmol) at room temperature. The reaction mixture was stirred for 6 h and then quenched with saturated aqueous $NaHCO_3$. H_2O was added to the mixture and extracted with CH_2Cl_2 3 times. The combined organic phase was washed with brine and dried over Na_2SO_4, filtered and concentrated under reduced pressure. The residue was purified with silica gel column chromatography eluting with Toluene/ ethylacetate (1/1) to afford 4,6-diol (60.8 mg, 70% yield) as a white solid. ^{1}H NMR (500 MHz, $CDCl_3$) δ 7.36 – 7.34 (m, 10H), 6.01 – 5.84 (m, 2H), 5.33 – 5.24 (m, 2H), 5.20 – 5.17 (m, 2H), 5.14 – 5.04 (m, 4H), 4.66 (q, *J* = 6.7 Hz, 2H), 4.37 (tq, *J* = 12.7, 1.43 Hz, 2H), 4.17 – 4.10 (m, 3H), 4.00 (dt, *J* = 9.3, 3.0 Hz, 1H), 3.95 - 3.89 (m, 2H), 3.33 (s, 3H), 3.13 (t, *J* = 9.2 Hz, 1H), 3.07 (dd, *J* = 9.9, 2.3 Hz, 1H), 2.82 (d, *J* = 2.9 Hz, 1H), 2.51 (d, *J* = 1.9 Hz, 1H); ^{13}C NMR (125 MHz, $CDCl_3$) δ 135.2, 134.1, 128.7, 128.7, 128.1, 128.1, 117.9, 117.2, 97.4, 82.1, 78.7, 78.6, 73.9, 72.7, 71.9, 71.9, 71. 0, 69.9, 69.6, 55.8 HRMS (ESI-QTOF) calculated for $C_{28}H_{37}NaO_{10}P$ $[M+Na]^+$ 587.2017, found 587.2039. To a solution of AllocOBT (863 mg, 3.94 mmol) and DMAP (300 mg, 2.46 mmol) in CH_2Cl_2 (5 mL) was added 4,6-diol (278 mg. 0.49 mmol). The reaction mixture was stirred for 6 h, and then quenched with saturated 10% aqueous citric acid. The reaction mixture was diluted with dichloromethane, and the solution was washed with saturated aqueous $NaHCO_3$ and brine. The organic solution was dried over anhydrous Na_2SO_4, filtered and concentrated *in vacuo*. The crude product was purified with column chromatography elution with toluene/ethyl acetate (5/1) to afford **7** (333 mg, 92% yield) as colorless oil. ^{1}H NMR (500 MHz, $CDCl_3$) δ 7.30-7.36 (m, 10H), 5.89-5.97 (m, 1H), 5.75-5.85 (m, 3H), 5.32-5.38 (m, 2H), 5.22-5.30 (m, 4H), 5.10-5.21 (m, 4H), 5.03 (dd, 4H, J = 7.9, 2.7 Hz), 4.73 (s, 2H), 4.65 (dt, 2H, J = 5.8, 1.3 Hz), 4.57 (ddt, 1H, J = 13.0, 5.7, 1.4 Hz), 4.40 (ddt, 1H, J = 13.0, 5.9, 1.4 Hz), 4.26-4.31 (m, 2H), 4.06-4.13 (m, 3H), 3.86 (ddt, 1H, J = 12.9, 5.5, 1.5 Hz), 3.43 (t, 1H, J = 9.5 Hz), 3.36 (s, 3H), 3.26 (dd, 1H, J = 10.1, 2.2 Hz); ^{13}C NMR (125 MHz, $CDCl_3$) δ 154.36, 134.31, 133.92, 131.61, 131.46, 128.68, 128.64, 128.00, 127.94, 119.08, 119.03, 117.34, 117.16, 97.49, 78.21, 77.36, 77.10, 76.85, 76.70, 76.32, 75.39, 75.35, 73.70, 72.68, 71.05, 69.62, 69.56, 68.77, 68.72, 56.01; HRMS (ESI-QTOF) calculated for $C_{36}H_{45}NaO_{14}P$ $[M+Na]^+$ 755.2439, found 755.2447

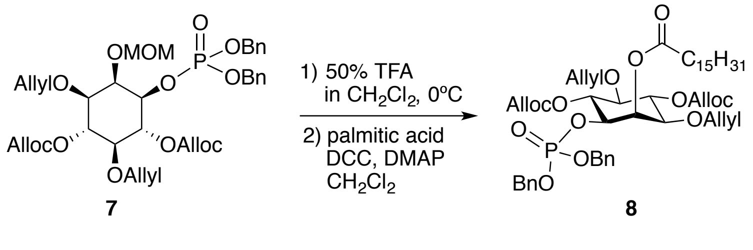

Synthesis of 3,5-di-*O*-allyloxy-4,6-di-*O*-allyloxycarbonyl-2-palmitoyl-D-*myo*-inositol-1-dibenzyl phosphate (8)

The solution of **7** (100 mg, 0.14 mmol) in CH_2Cl_2 (6.8 mL) was cooled to 0 °C. To the solution was added trifluoroacetic acid 6.8 mL) at 0 ºC and stirred for 1h. The solvent removed under reduced pressure and added toluene then concentrated *in vacuo*. The residue was dried under highly vacuum and used for next step without further purification. The residue and palmitic acid (174 mg, 0.68 mmol) and DMAP (16.6 mg, 0.14 mmol) were dissolved with CH_2Cl_2 (1 mL) at room temperature. To the stirred mixture was added DCC (130 mg, 0.68 mmol) and stirred for over night. The mixture was filtered and concentrated under reduced pressure. The residue was purified with silica gel column chromatography eluting with Toluene/ ethyl acetate (20/1 to 10/1) to afford **8** (84.7 mg, 67% yield) as colorless oil. ^{1}H NMR (400 MHz, $CDCl_3$) δ 7.28-7.35 (m, 10H), 5.93 (ddd, 1H, J = 22.7, 10.8, 5.6 Hz), 5.71-5.84 (m, 4H), 5.36 (dd, 1H, J = 17.3, 1.3 Hz), 5.23-5.30 (m, 4H), 5.10-5.19 (m, 5H), 4.95-5.05 (m, 4H), 4.66 (d, 2H, J = 5.8 Hz), 4.50-4.57 (m, 2H), 4.31 (dd, 1H, J = 13.0, 5.8 Hz), 4.08-4.18 (m, 5H), 3.86 (dd, 1H, J = 12.8, 5.8 Hz), 3.43-3.51 (m, 2H), 2.31-2.43 (m, 2H), 1.56-1.64 (m, 2H), 1.23-1.30 (m, 24H), 0.88 (t, 3H, J = 6.8 Hz); ^{13}C NMR (150 MHz, $CDCl_3$) δ 177.85, 172.75, 171.27, 154.33, 154.28, 135.65, 135.59, 134.11, 133.72, 131.50, 131.34, 128.58, 128.53, 128.32, 127.93, 127.74, 119.18, 119.13, 117.70, 117.51, 77.95, 77.46, 77.14, 76.81, 76.18, 74.69, 74.23, 73.78, 70.86, 69.59, 69.44, 68.86, 68.82, 67.68, 60.51, 34.22, 33.99, 32.03, 29.81, 29.79, 29.58, 29.47, 29.38, 29.22, 29.09, 25.07, 24.89, 22.79, 21.15, 14.29, 14.23; HRMS (ESI-QTOF) calculated for $C_{50}H_{71}NaO_{14}P$ $[M+Na]^+$ 949.4477, found 949.4511

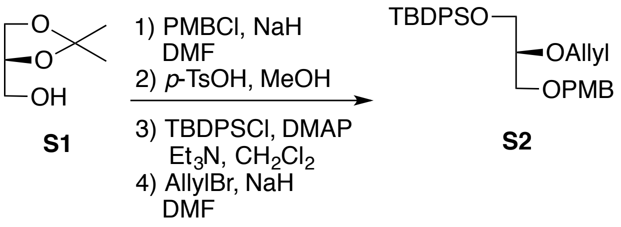

2-allyl-3-*tert*-butyldiphenylsilyl-1-(4-methoxybenzyl)-*sn*-glycerol (S2)

To a stirred solution of (*S*)-(+)-2,2-dimethyl-1,3-dioxolane-4-methanol (**S1**) (10 g, 75.7 mmol) in DMF (200 mL) were added NaH (60% oil dispersion) (4.5 g, 0.11 mol) and then PMBCl (12.4 mL, 91.0 mmol) and the mixture was stirred for 4 h. The reaction was quenched with MeOH and ans the mixture was extracted with CH_2Cl_2. The extract was washed with brine, dried over Na_2SO_4, and concentrated under reduced pressure. To the solution of the residue in MeOH (200 mL) was added *p*-toluene sulfonic acid monohydrate (700 mg, 3.68 mmol). After stirring for 1 h, the reaction was quenched by addition of $NaHCO_3$ (1 g) and the solvent was removed under reduced pressure. The residue was purified with silica

gel column chromatography (hexane/ethyl acetate = 1/3) to afford diol (7.9 g, 50% yield, for 2 steps) as a colorless oil. To a stirred solution of the diol (7.9 g, 41.9 mmol), Et_3N (26 mL, 0.19 mol), and DMAP (590 g, 4.83 mmol) in CH_2Cl_2 (74 mL) was added TBDPSCl (11 mL, 42.8 mmol). After stirring for over night, the reaction was extracted with CH_2Cl_2. The extract was washed with brine, dried over Na_2SO_4, and concentrated under reduced pressure. The residue was purified with silica gel column chromatography (hexane/ethyl acetate = 5/1) to afford *sn*-2 alcohol (14.6 g, 87% yield) as a colorless oil. To a stirred solution of *sn*-2 alcohol (2.0 g, 4.44 mmol) in DMF (13 mL) at 0 ºC was added NaH (60% oil dispersion) (242 mg, 5.77 mmol). After stirring for 15 min, allyl bromide (0.60 mL, 6.66 mmol) was added and then the mixture was stirred for 2 d at room temperature. The reaction was quenched with 10% citric acid aq. and extracted with ethyl acetate. The extract was washed with brine, dried over Na_2SO_4, and concentrated under reduced pressure. The residue was purified with silica gel column chromatography (hexane/ethyl acetate = 10/1) to afford **S2** (1.54 g, 71% yield) as a colorless oil. ^{1}H NMR (400 MHz, $CDCl_3$) δ 7.65-7.67 (m, 4H), 7.34-7.43 (m, 6H), 7.22-7.24 (m, 2H), 6.83-6.87 (m, 2H), 5.84-5.92 (m, 1H), 5.23 (dq, 1H, *J* = 17.2, 1.7 Hz), 5.12 (dq, 1H, *J* = 10.4, 1.4 Hz), 4.47 (dd, 2H, *J* = 16.8, 11.6 Hz), 4.07-4.09 (m, 2H), 3.79 (s, 3H), 3.73 (t, 2H, *J* = 2.6 Hz), 3.61-3.65 (m, 2H), 3.52-3.56 (m, 1H), 1.03 (s, 9H); ^{13}C NMR (125 MHz, $CDCl_3$) δ 159.21, 135.74, 135.71, 135.37, 133.66, 133.59, 130.59, 129.72, 129.33, 127.75, 116.73, 113.83, 78.82, 73.13, 71.39, 69.92, 63.56, 55.36, 26.90, 19.31; HRMS (ESI-QTOF) calcd for $C_{30}H_{38}NaO_4Si$ $[M+Na]^+$, 513.2432; found: 513.2439

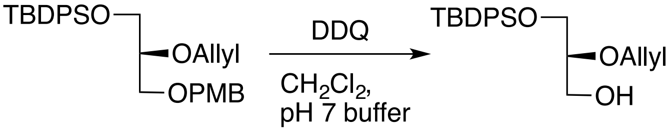

S2 **S3**

2-allyl-3-*tert*-butyldiphenylsilyl-*sn*-glycerol (S3)

To a stirred mixture of **S2** (0.85 g, 1.73 mmol) in CH_2Cl_2 (50 mL) and pH 7 phosphate buffer (5.5 mL) was added DDQ (787 mg, 3.47 mmol). After stirring for over night, the mixture was extracted with CH_2Cl_2. The extract was washed with saturated $NaHCO_3$ aq. and brine, dried over Na_2SO_4, and concentrated under reduced pressure. The residue was purified with silica gel column chromatography (hexane/ethyl acetate = 5/1) to afford **S3** (528 mg, 82% yield) as a colorless oil. ^{1}H NMR (400 MHz, $CDCl_3$) δ 7.66-7.68 (m, 4H), 7.37-7.46 (m, 6H), 5.82-5.91 (m, 1H), 5.20-5.26 (m, 1H), 5.13-5.16 (m, 1H), 4.08 (ddt, 1H, *J* = 12.7, 5.6, 1.4 Hz), 3.99 (ddt, 1H, *J* = 12.7, 5.7, 1.3 Hz), 3.73-3.84 (m, 2H), 3.65-3.70 (m, 2H), 3.51-3.58 (m, 1H), 2.05-2.09 (m, 1H), 1.05 (s, 9H); ^{13}C NMR (100 MHz, $CDCl_3$) δ 135.70, 135.67, 134.87, 133.32, 133.20, 129.89, 127.84, 117.24, 79.40, 71.24, 63.53, 62.95, 26.89, 19.25; HRMS

(ESI-QTOF) calcd for $C_{22}H_{30}NaO_3Si$ $[M+Na]^+$, 393.1856; found: 393.1858

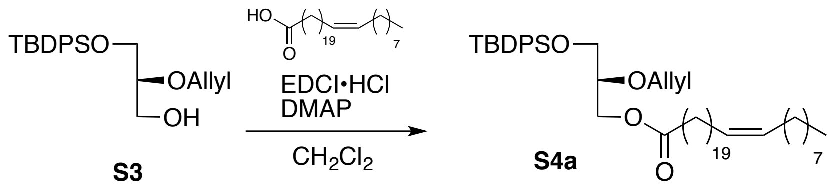

2-allyl-3-*tert*-butyldiphenylsilyl-1-(*cis*-21-triacontenyl)-*sn*-glycerol (S4a)

To a stirred solution of $C_{30}H_{57}COOH$ (557 mg, 1.10 mmol) in CH_2Cl_2 (5.5 mL) was added trifluoroacetic acid (5.5 mL) and the mixture was stirred for 1 h at room temperature. The solvent was removed under reduced pressure and co-evaporated with toluene three times. To the residue was added the solution of **S3** (488 mg, 1.32 mmol) and DMAP (67 mg, 0.55 mmol) in toluene (5.5 mL). EDCI•HCl (632 mg, 3.30 mmol) was added to the reaction mixture and the mixture was stirred and heated under reflux for over night. The reaction mixture was extracted with ethyl acetate. The extract was washed with water and brine, dried over Na_2SO_4, and concentrated under reduced pressure. The residue was purified with silica gel column chromatography (hexane/ethyl acetate = 15/1) to afford **S4a** (839 mg, 95%) as a colorless oil. ^{1}H NMR (400 MHz, $CDCl_3$) δ 7.66-7.68 (m, 4H), 7.36-7.46 (m, 6H), 5.80-5.90 (m, 1H), 5.32-5.39 (m, 2H), 5.23 (dq, 1H, *J* = 17.2, 1.6 Hz), 5.14 (ddd, 1H, *J* = 10.3, 2.9, 1.2 Hz), 4.32 (dd, 1H, *J* = 11.6, 3.9 Hz), 4.14-4.18 (m, 1H), 3.99-4.08 (m, 2H), 3.68-3.75 (m, 2H), 3.61-3.67 (m, 1H), 2.29 (t, 2H, *J* = 7.6 Hz), 1.97-2.04 (m, 3H), 1.58-1.67 (m, 2H), 1.24-1.34 (m, 44H), 1.05 (s, 9H), 0.88 (t, 3H, *J* = 6.8 Hz); ^{13}C NMR (100 MHz, $CDCl_3$) δ 173.82, 135.68, 134.70, 133.31, 130.00, 129.81, 127.79, 117.21, 77.25, 71.30, 63.58, 63.08, 34.35, 32.01, 29.80, 29.58, 29.42, 29.26, 27.31, 26.86, 25.03, 22.78, 19.28, 14.22; HRMS (ESI-QTOF) calcd for $C_{52}H_{86}NaO_4Si$ $[M+Na]^+$, 825.6188; found: 825.6207

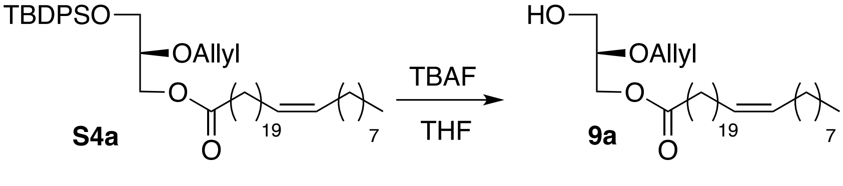

2-allyl-1-(*cis*-21-triacontenyl)-*sn*-glycerol (9a)

To a stirred solution of **S4a** (300 mg, 0.37 mmol) and AcOH (22 μL) in THF (3.7 mL) was added TBAF (1 M in THF) (0.82 mL, 0.82 mmol). After stirring for over night, the solvent was removed under reduced pressure. The crude mixture was purified with silica gel column chromatography (hexane/ethyl acetate = 5/1) to afford **9a** (191 mg, 90% yield) as a white solid. ^{1}H NMR (400 MHz, $CDCl_3$) δ 5.88-5.95 (m, 1H),

5.34-5.39 (m, 2H), 5.30 (dq, 1H, *J* = 17.2, 1.6 Hz), 5.21 (dq, 1H, *J* = 10.4, 1.4 Hz), 4.15-4.20 (m, 3H), 4.09 (ddt, 1H, *J* = 12.7, 5.8, 1.3 Hz), 3.59-3.73 (m, 3H), 2.33 (t, 2H, *J* = 7.6 Hz), 1.95-2.03 (m, 4H), 1.61 (q, 2H, *J* = 7.3 Hz), 1.25-1.34 (m, 44H), 0.88 (t, 3H, *J* = 6.9 Hz); ^{13}C NMR (125 MHz, $CDCl_3$) δ 173.91, 134.54, 129.98, 117.68, 71.27, 62.77, 62.10, 34.29, 32.00, 29.86, 29.79, 29.75, 29.70, 29.65, 29.61, 29.55, 29.41, 29.35, 29.22, 27.29, 25.01, 22.77, 14.19; HRMS (ESI-QTOF) calcd for $C_{36}H_{68}NaO_4$ $[M+Na]^+$, 580.5010; found: 587.5021

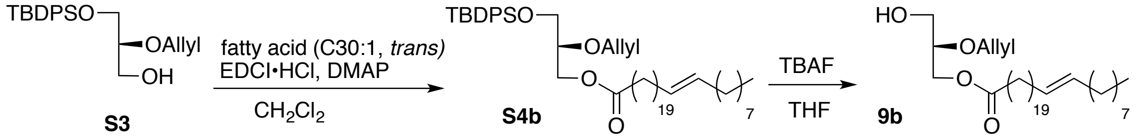

2-allyl-1-(*trans*-21-triacontenyl)-*sn*-glycerol (9b)

To the solution of fatty acid (C30:1, *trans*) ^{t}Bu ester (253 mg, 0.50 mmol) in CH_2Cl_2 (2.5 mL) was added TFA (2.5 mL) at room temperature and stirred for 1h. The solvent was removed under reduced pressure and dried under highly vacuumed. The residue and **S3** (370 mg, 1.0 mmol) was dissolved with CH_2Cl_2/toluene (1/1) (5 mL) and then added DMAP (6.25 mg, 51 μmol) and DCC (400 mg, 1.94 mmol). The mixture was stirred for over night at room temperature. The mixture was filtered and concentrated under reduced pressure. The residue was roughly purified with silica gel column chromatography (Hexane/ethyl acetate = 5/1). The mixture was dissolved with THF (4 mL) and added TBAF (1 M in THF) (1 mL, 1.0 mmol) and stirred for over night. The mixture was concentrated *in vacuo*. The residue was purified with silica gel column chromatography (Hexane/ethyl acetate = 5/1) to afford **9b** (176 mg, 62% yield for 3 steps) as a white solid. ^{1}H NMR (500 MHz, $CDCl_3$) δ 5.95 – 5.87 (m, 1H), 5.38 (t, *J* = 3.7 Hz, 2H), 5.30 (dq, *J* = 17.2, 1.4 Hz, 1H), 5.21 (dd, *J* = 10.3, 1.1 Hz, 1H), 4.22 – 4.07 (m, 5H), 3.70 – 3.60 (m, 3H), 2.33 (t, *J* = 7.6 Hz, 2H), 1.96 (q, *J* = 6.2 Hz, 4H), 1.62 (quin, *J* = 7.3 Hz, 2H), 1.34 – 1.24 (m, 44H), 0.88 (t, *J* = 6.7 Hz, 3H); ^{13}C NMR (125 MHz, $CDCl_3$) δ 173.9, 134.5, 130.4, 117.7, 71.3, 62.8, 62.1, 60.5, 34.3, 32.7, 32.0, 29.8, 29.7, 29.7, 29.6, 29.6, 29.6, 29.5, 29.4, 29.3, 29.3, 29.2, 25.0, 22.8, 14.3, 14.2, 21.1; HRMS (ESI-QTOF) calculated for $C_{36}H_{68}NaO_4$ $[M+Na]^+$ 587.5010, found 587.5020

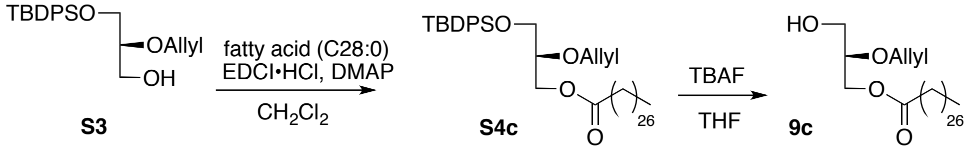

2-allyl-1-octacosanoyl-*sn*-glycerol (9c)

In a manner similar to the synthesis of **9b, S3** (200 mg, 0.416 mmol) was converted into **9c** (144 mg, 64% yield for 3 steps) as a white solid. ^{1}H NMR (500 MHz, $CDCl_3$) δ 5.94 – 5.86 (m, 1H), 5.29 (dq, J = 17.2, 1.6 Hz, 1H), 5.19 (dq, J = 10.3, 1.1 Hz, 1H), 4.19 – 4.14 (m, 3H), 4.08 (ddt, J = 12.7, 5.9, 1.3 Hz, 1H), 3.69 – 3.60 (m, 3H), 2.32 (t, J = 7.4 Hz, 2H), 1.61 (quin, J = 7.6 Hz, 2H), 1.29 – 1.21 (m, 48H), 0.87 (t, J = 6.9 Hz, 3H); ^{13}C NMR (125 MHz, $CDCl_3$) δ 173.9, 134.5, 117.7, 71.3, 62.8, 62.1, 34.3, 32.1, 29.8, 29.7, 29.7, 29.5, 29.4, 29.3, 29.2, 25.0, 22.8, 14.2; HRMS (ESI-QTOF) calculated for $C_{34}H_{66}NaO_4$ $[M+Na]^+$ 561.4853, found 561.4864

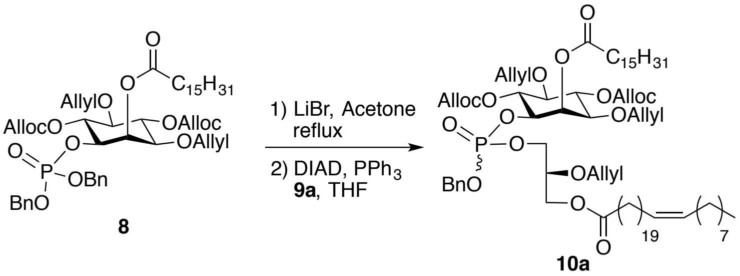

Synthesis of 3,5-di-allyl-4,6-di-*O*-allyloxycarbonyl-2-allyl-3-*O*-((*cis*-21-triacontenyl)-*sn*-glycerol-1-benzylphosphate)-2-palmitoyl-D-*myo*-inositol (10a)

To a stirred solution of **8** (38.1 mg, 41.1 μmol) in Acetone (2.7 mL) was added LiBr (7.2 mg, 82.2 μmol). The reaction mixture was refluxed for 1 d. The mixture was cooled to ambient temperature, and then concentrated under reduced pressure. The residue was purified with silica gel column chromatography eluting with Toluene/AcOEt (3 : 1) to CH_2Cl_2/MeOH (6 : 1) to afford lithium salt. The salt was passed through the cation exchange resin column (Dowex 50WX8 50-100) to obtain proton form. Fractions containing the desire product was collected and concentrated under reduced pressure. The residue was dissolved with THF (411 μL). **9a** (46.4 mg, 82.2 μmol), PPh_3 (21.6 mg, 82.2 μmol) was added to the solution. The mixture was cooled to 0 °C, and then DIAD (16 μL, 82.2 μmol) was added to the reaction mixture. The reaction was warmed to room temperature. After stirred for 1d, the reaction mixture was concentrated under reduced pressure. The residue was purified with Preparative TLC (Toluene/AcOEt = 6 / 1) to afford **10a** (37 mg, 60% for 2 steps) (~1:1 diasteromixture) as colorless oil. ^{1}H NMR (500 MHz, $CDCl_3$) δ 7.30-7.40 (m, 5H), 5.72-5.97 (m, 6H), 4.97-5.38 (m, 18H), 4.28-4.71 (m, 5H), 3.98-4.21 (m, 9H), 3.85-3.90 (m, 1H), 3.64-3.72 (m, 1H), 3.42-3.50 (m, 2H), 2.33-2.44 (m, 2H), 2.29 (dd, 2H, J = 14.9, 7.2 Hz), 1.94-2.03 (m, 4H), 1.56-1.65 (m, 4H), 1.25-1.33 (m, 68H), 0.88 (t, 6H, J = 6.9 Hz); ^{13}C NMR (125 MHz, $CDCl_3$) δ 173.58, 172.60, 154.31, 134.48, 134.11, 133.74, 131.51, 129.98, 128.62, 127.96, 127.77, 119.11, 117.63, 117.49, 77.94, 77.36, 77.10, 76.85, 76.16, 75.08, 74.76, 74.21, 72.39, 71.36, 70.88, 70.39, 69.63, 68.98, 68.86, 68.80, 67.66, 66.34, 62.51, 34.19, 32.02, 29.86, 29.81, 29.65, 29.61,

29.45, 29.41, 29.26, 29.13, 27.29, 25.14, 24.96, 22.78, 22.00, 21.80, 14.20; HRMS (ESI-QTOF) calculated for $C_{79}H_{131}NaO_{17}P$ $[M+Na]^+$ 1405.9016, found 1405.9041

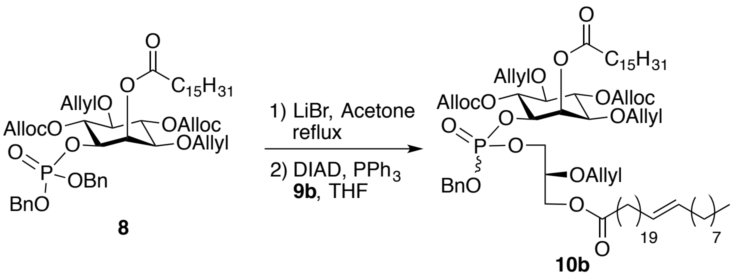

Synthesis of 3,5-di-allyl-4,6-di-*O*-allyloxycarbonyl-2-allyl-3-*O*-((*trans*-21-triacontenyl)-*sn*-glycerol-1-benzylphosphate)-2-palmitoyl-D-*myo*-inositol (10b)

In a manner similar to the synthesis of **10a, 8** (55 mg, 59.4 μmol) was converted into **10b** (52.3 mg, 60% for 2 steps) (~1:1 diasteromixture) as a color less oil. ^{1}H NMR (500 MHz, $CDCl_3$) δ 7.30-7.39 (m, 5H), 5.72-5.97 (m, 6H), 4.97-5.39 (m, 18H), 4.31-4.71 (m, 5H), 3.98-4.21 (m, 9H), 3.85-3.90 (m, 1H), 3.64-3.72 (m, 1H), 3.43-3.51 (m, 2H), 2.35-2.43 (m, 2H), 2.27-2.33 (m, 2H), 1.96 (dd, 4H, J = 11.0, 6.1 Hz), 1.58-1.68 (m, 4H), 1.25-1.32 (m, 68H), 0.88 (t, 6H, J = 6.9 Hz); ^{13}C NMR (125 MHz, $CDCl_3$) δ 173.50, 172.65, 154.31, 134.48, 134.11, 133.74, 131.51, 131.46, 131.36, 130.44, 128.64, 128.62, 127.95, 127.76, 119.22, 119.14, 119.09, 117.67, 117.61, 117.47, 77.93, 77.38, 77.12, 76.86, 76.17, 75.08, 74.75, 74.72, 74.20, 72.35, 71.36, 71.28, 70.87, 70.83, 70.40, 69.63, 68.97, 68.85, 68.80, 62.50, 34.27, 34.18, 32.69, 32.01, 31.98, 29.80, 29.74, 29.62, 29.57, 29.45, 29.39, 29.25, 29.12, 29.08, 25.14, 25.04, 24.96, 22.77, 22.00, 21.80, 14.19; HRMS (ESI-QTOF) calculated for $C_{79}H_{131}NaO_{17}P$ $[M+Na]^+$ 1405.9016, found 1405.9041

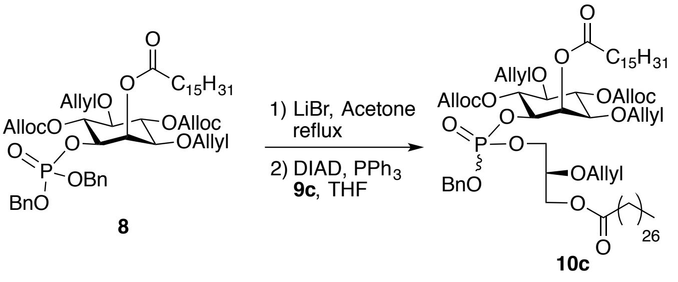

Synthesis of 3,5-di-allyl-4,6-di-*O*-allyloxycarbonyl-(2-allyl-1-*O*-((octacosanyl)-*sn*-glycerol-3-benzylphosphate))-2-palmitoyl-D-*myo*-inositol (10c)

In a manner similar to the synthesis of **10a**, **8** (62.5 mg, 67.5 μmol) was converted into **10c** (65.8 mg, 72% for 2 steps) (~1:1 diasteromixture) as a color less oil. ^{1}H NMR (500 MHz, $CDCl_3$) δ 7.29-7.40 (m, 5H), 5.71-5.96 (m, 6H), 4.94-5.38 (m, 14H), 4.31-4.70 (m, 5H), 3.98-4.23 (m, 9H), 3.87 (ddd, 1H, J =

12.9, 5.6, 4.3 Hz), 3.62-3.72 (m, 1H), 3.42-3.50 (m, 2H), 2.36-2.44 (m, 2H), 2.28 (q, 2H, J = 7.4 Hz), 1.53-1.64 (m, 4H), 1.25-1.31 (m, 72H), 0.87 (t, 6H, J = 6.9 Hz); ^{13}C NMR (125 MHz, $CDCl_3$) δ 173.51, 172.60, 154.31, 154.27, 138.00, 134.49, 134.12, 133.75, 131.52, 131.46, 129.12, 128.65, 128.30, 127.96, 127.77, 125.38, 119.10, 117.62, 117.48, 77.94, 77.37, 77.12, 76.97, 76.86, 76.17, 75.09, 74.76, 74.21, 71.36, 71.31, 70.88, 69.63, 68.98, 68.86, 68.80, 67.65, 66.64, 66.52, 66.35, 62.58, 62.51, 34.28, 34.19, 32.02, 29.79, 29.75, 29.59, 29.45, 29.39, 29.25, 29.13, 29.09, 25.14, 25.05, 24.96, 22.78, 21.53, 14.21; HRMS (ESI-QTOF) calculated for $C_{77}H_{129}NaO_{17}P$ $[M+Na]^+$ 1379.8860, found 1379.8861

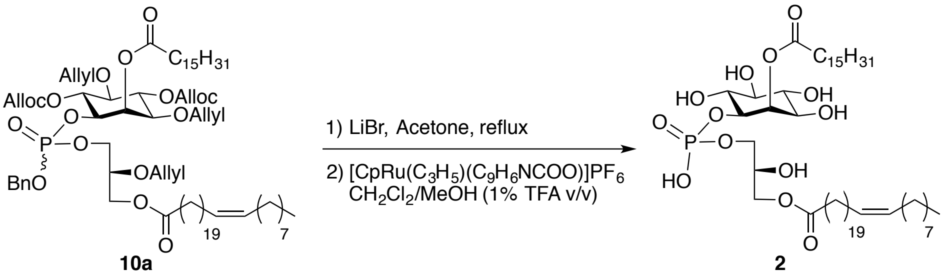

Synthesis of *Eh*PIb C30:1 *cis* (2)

To a stirred solution of **10a** (22.8 mg, 16.4 µmol) in Acetone (1.1 mL) was added LiBr (28.6 mg, 0.33 mmol) and refluxed for 6 h. After the removal of solvent, the crude mixture was subjected to silica gel column chromatography (Toluene/ethyl acetate = 5/1 to CH_2Cl_2/MeOH = 6/1) to afford lithium salt. The salt was dissolved in CH2Cl2/MeOH = 1/1 containing 1% TFA (vol/vol) (1 mL). $[CpRu(C_3H_5)(C_9H_6NCOO)]PF_6$ (8.6 mg, 1.6 µmol) was added to reaction mixture. After stirring for 3 h, the solvent was removed under reduced pressure. The crude mixture was purified by silica gel column chromatography ($CHCl_3$/MeOH/2.2 M NH_4OH = 45/10/1.4) to afford **2** (14.6 mg, 88% yield for 2 steps) as a white solid. ^{1}H NMR (600 MHz, $CDCl_3/CD_3OD/D_2O$) δ 5.57-5.58 (m, 1H), 5.36 (t, 2H, *J* = 4.7 Hz, –CH_2C*H*=C*H*CH_2–) , 4.09-4.17 (m, 3H, ino-1, Gly-3x2), 3.89 (1H, Gly-2), 3.82 (m, 1H, Gly-1), 3.73-3.77 (m, 1H, Gly-1), 3.64 (t, 1H, *J* = 9.0 Hz, ino-6), 3.54 (dd, 1H, *J* = 2.4, 9.4 Hz, ino-3), 3.49 (t, 1H, *J* = 9.5 Hz, ino-4), 3.25 (1H, ino-5), 2.35-2.41 (m, 4H, –COCH_2–), 2.03 (dd, 4H, *J* = 12.5, 6.7 Hz, –CH_2CH=CHCH_2–), 1.59-1.64 (m, 4H, –COCH$_2$CH_2–), 1.28-1.35 (m, 68H, –CH_2–), 0.89 (t, 6H, *J* = 6.9 Hz, –CH_3); ^{13}C NMR (150 MHz, $CDCl_3/CD_3OD/D_2O$) δ 174.43, 174.40, 129.60, 74.06, 72.91, 72.81, 72.57, 72.22, 69.75, 68.54, 66.53, 64.79, 33.92, 33.73, 31.61, 31.58, 30.12, 29.41, 29.38, 29.33, 29.30, 29.27, 29.13, 29.11, 29.05, 29.02, 28.97, 28.94, 28.91, 28.87, 26.83, 26.80, 24.58, 24.53, 22.32, 13.54; 31P NMR (125 MHz, $CDCl_3/CD_3OD/D_2O$) δ 1.21; HRMS (ESI-Q-TOF) calculated for $C_{55}H_{104}O_{13}P^-$ $[M]^-$ 1003.7220, found 1003.7219

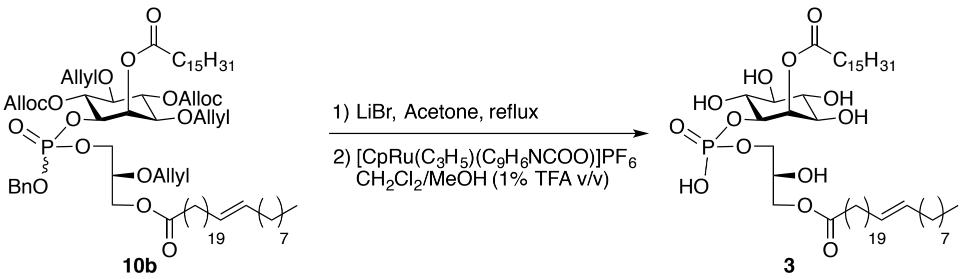

Synthesis of *Eh*PIb C30:1 *trans* (3)

In a manner similar to the synthesis of **2**, **10b** (10 mg, 7.22 μmol) was converted into **3** (5.46 mg, 75% yield for 2 steps) as a white solid. ^{1}H NMR (600 MHz, $CDCl_3/CD_3OD/D_2O$) δ 5.58 (1H, ino-2), 5.39 (t, 2H, *J* = 3.7 Hz, –$CH_2CH{=}CHCH_2$–), 4.10-4.17 (m, 3H, ino-1, Gly-3x2), 3.97-4.01 (m, 1H, Gly-2), 3.90-3.96 (m, 1H, Gly-1), 3.83-3.88 (m, 1H, Gly-1), 3.75 (t, 1H, *J* = 9.4 Hz), 3.64 (dd, 1H, *J* = 10.8, 2.4 Hz, ino-3), 3.59 (t, 1H, *J* = 9.5 Hz, ino-4), 3.34 (1H, ino-5), 2.35-2.41 (m, 4H, –$COCH_2$–), 1.96-2.00 (m, 4H, –$CH_2CH{=}CHCH_2$–), 1.60-1.65 (m, 4H, –$COCH_2CH_2$–), 1.27-1.36 (m, 68H, –CH_2–), 0.89 (t, 6H, *J* = 7.0 Hz, –CH_3); ^{13}C NMR (150 MHz, $CDCl_3/CD_3OD/D_2O$) δ 175.18, 174.59, 130.85, 74.93, 74.78, 73.57, 73.38, 72.98, 70.73, 70.49, 69.44, 69.28, 67.36, 65.57, 65.48, 58.88, 57.18, 57.08, 34.68, 34.47, 32.93, 32.34, 32.31, 30.11, 30.01, 29.97, 29.85, 29.76, 29.70, 29.67, 29.63, 29.47, 29.41, 25.32, 25.28, 23.05, 14.25; ^{31}P NMR (125 MHz, $CDCl_3/CD_3OD/D_2O$) δ 1.50; HRMS (ESI-Q-TOF) calculated for $C_{55}H_{104}O_{13}P^-$ $[M]^-$ 1003.7220, found 1003.7222

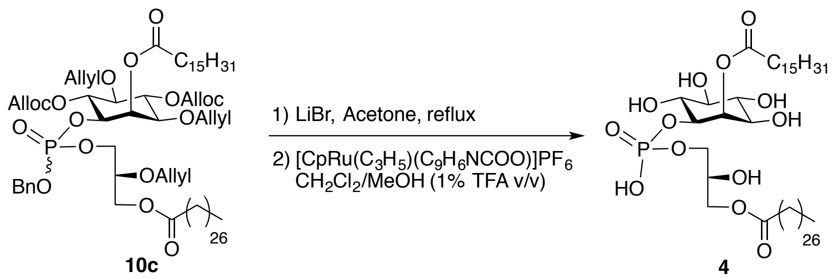

Synthesis of *Eh*PIb C28:0 (4)

In a manner similar to the synthesis of **2**, **10c** (8.8 mg, 6.48 μmol) was converted into **4** (5.21 mg, 82% yield for 2 steps) as a white solid. ^{1}H NMR (600 MHz, $CDCl_3/CD_3OD/D_2O$) δ 5.59 (dd, 1H, *J* = 2.4, 2.4 Hz, ino-2), 4.10-4.20 (m, 3H, ino-1, Gly-3x2), 3.98-4.01 (m, 1H, Gly-2), 3.93-3.96 (m, 1H, Gly-1), 3.84-3.89 (m, 1H, Gly-1), 3.75 (t, 1H, *J* = 9.6 Hz, ino-6), 3.58-3.65 (m, 2H, ino-4, ino-3), 3.56 (1H, ino-5), 2.35-2.41 (m, 4H, –$COCH_2$–), 1.61-1.65 (m, 4H, –$COCH_2CH_2$–), 1.28-1.34 (m, 72H, –CH_2–), 0.89 (t, 6H, *J* = 7.0 Hz, –CH_3); ^{13}C NMR (150 MHz, $CDCl_3/CD_3OD/D_2O$) δ 174.13, 174.52, 73.94, 73.80, 72.62, 72.36, 72.03, 69.51, 68.25, 66.28, 64.54, 33.66, 33.45, 31.31, 30.89, 28.92, 28.74, 24.31, 24,27,

22.03, 13.17; ^{31}P NMR (125 MHz, $CDCl_3/CD_3OD/D_2O$) δ 1.15; HRMS (ESI-Q-TOF) calculated for $C_{53}H_{102}O_{13}P^-$ [M]$^-$ 977.7064, found 977.7063

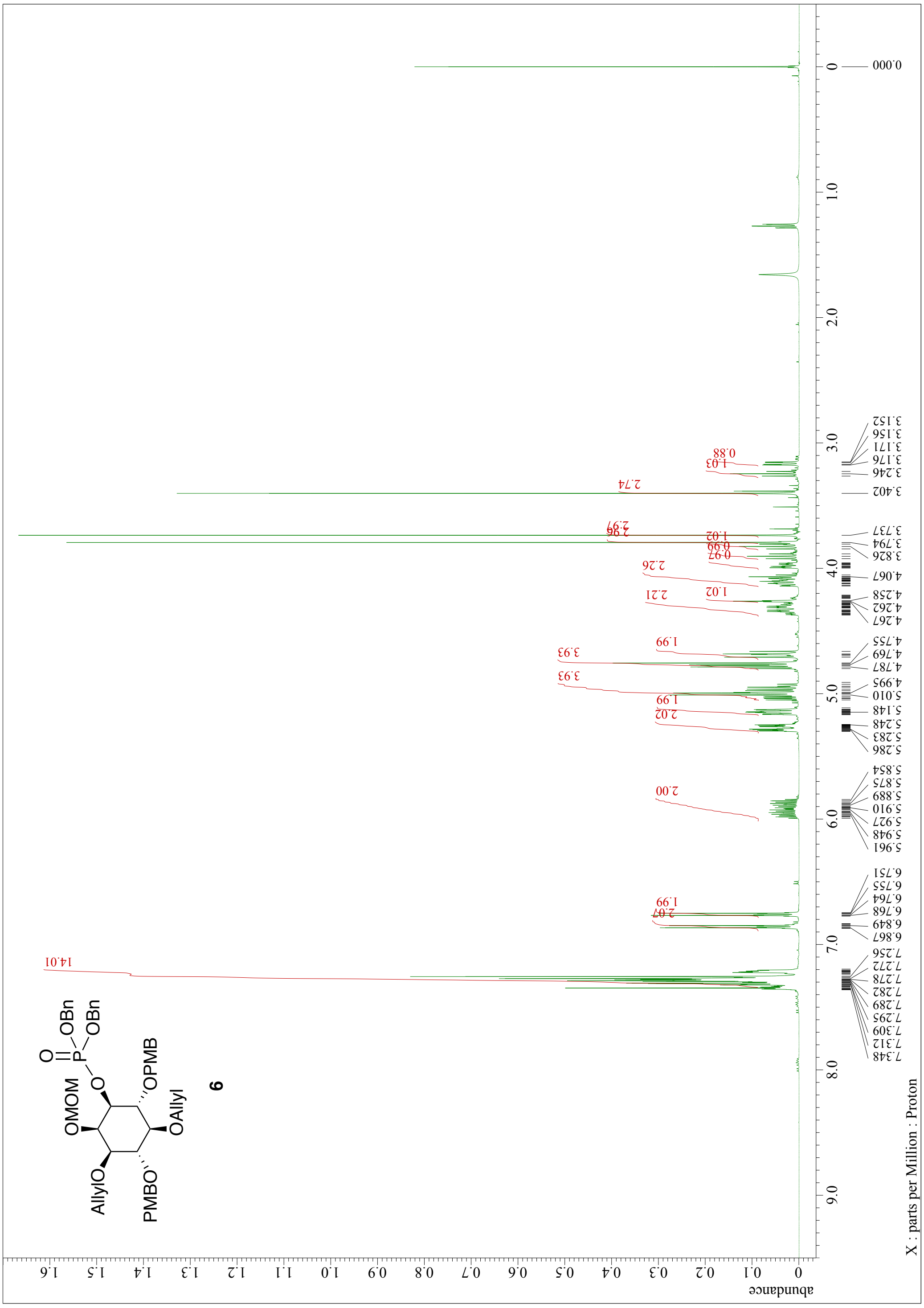

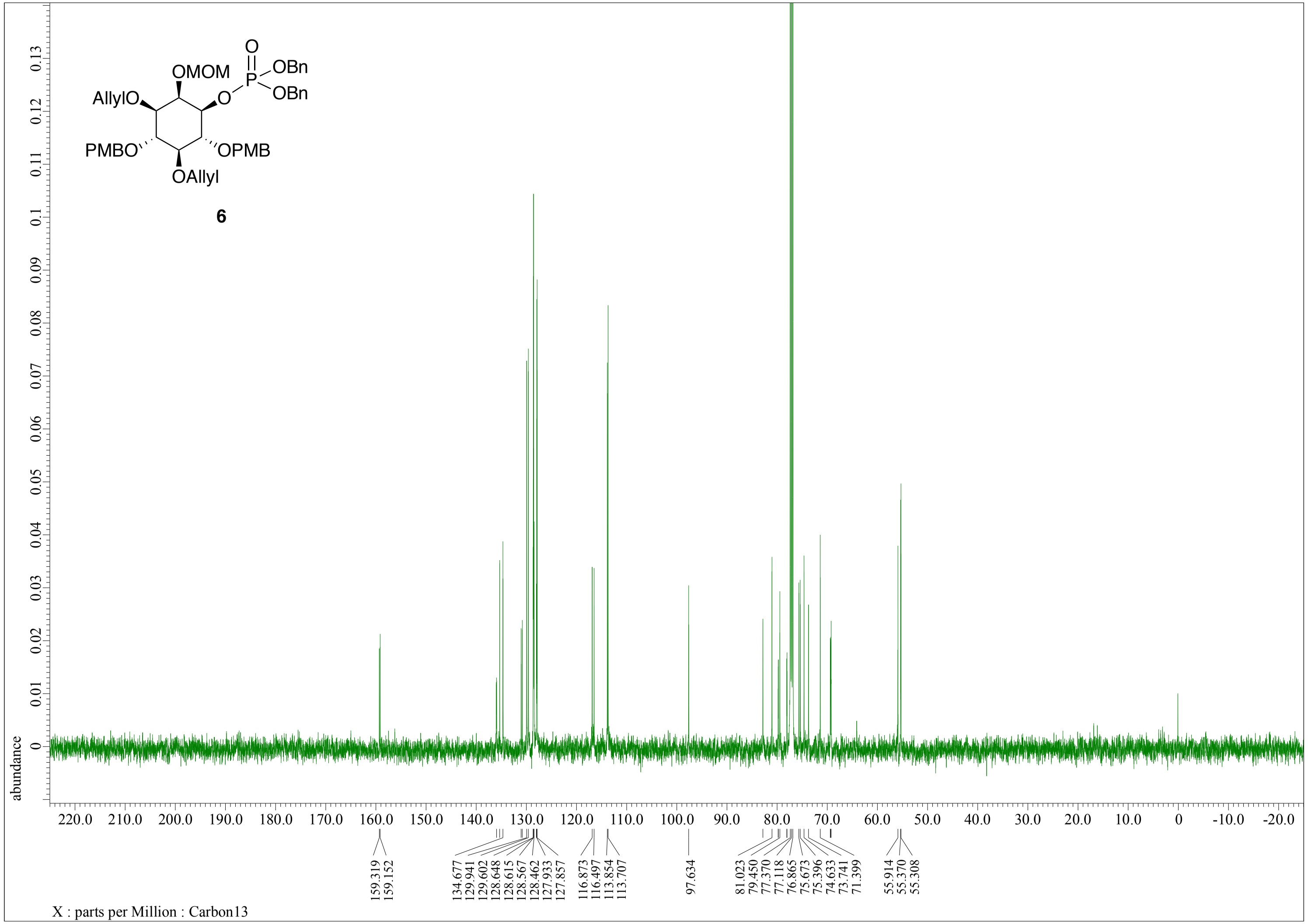

OMOM
AllylO
PMBO
OPMB
OAllyl
O
P
OBn
OBn
6
abundance
X : parts per Million : Carbon13
159.319
159.152
134.677
129.941
129.602
128.648
128.615
128.567
128.462
127.933
127.857
116.873
116.497
113.854
113.707
97.634
81.023
79.450
77.370
77.118
76.865
75.673
75.396
74.633
73.741
71.399
55.914
55.370
55.308

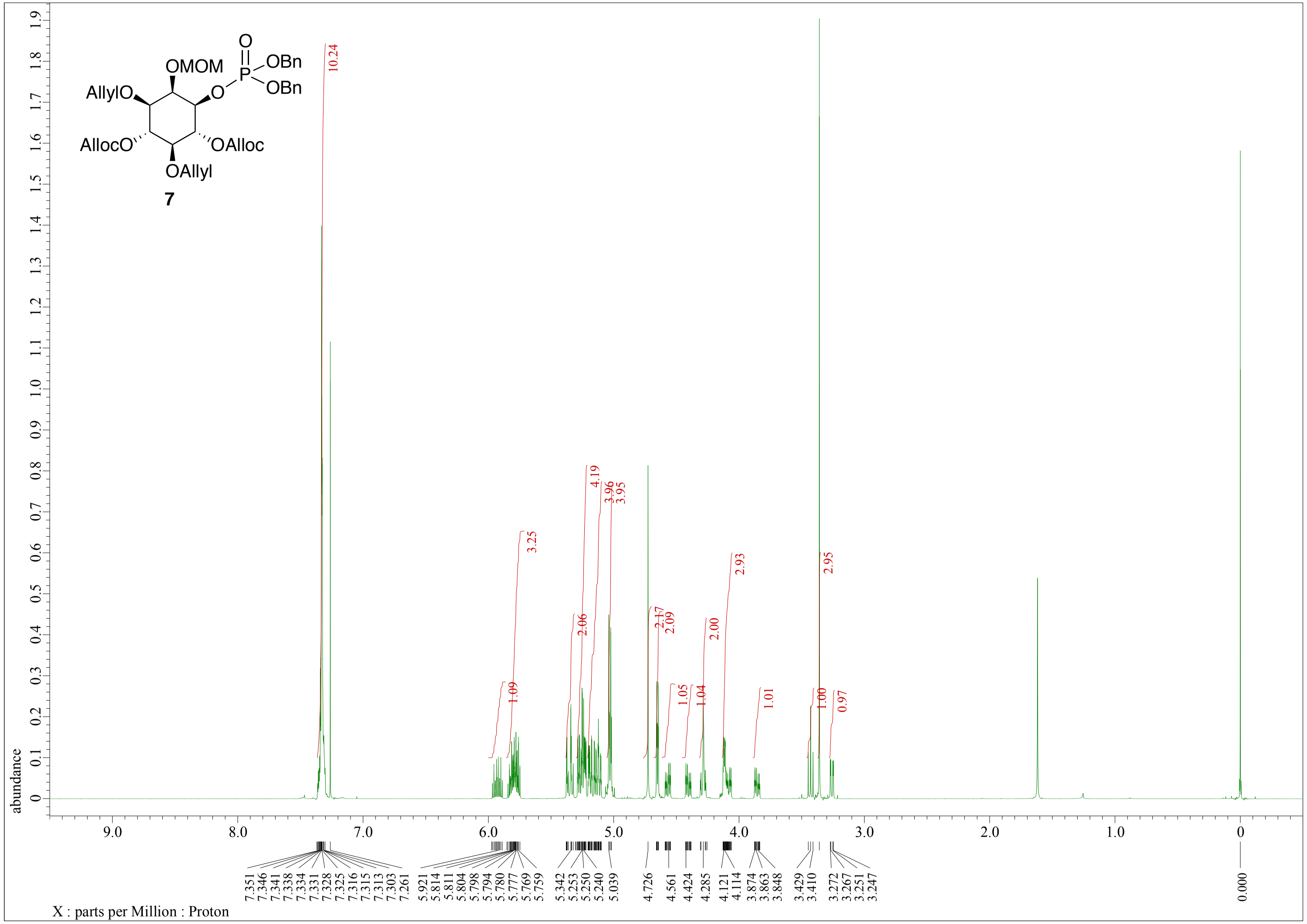

AllylO
OMOM
O
P
OBn
OBn
AllocO
OAlloc
OAllyl
7
abundance
10.24
3.25
1.09
2.06
4.19
3.96
3.95
2.17
2.09
1.05
1.04
2.00
2.93
1.01
1.00
2.95
0.97
7.351
7.346
7.341
7.338
7.334
7.331
7.328
7.325
7.316
7.315
7.313
7.303
7.261
5.921
5.814
5.811
5.804
5.798
5.794
5.780
5.777
5.769
5.759
5.342
5.253
5.250
5.240
5.039
4.726
4.561
4.424
4.285
4.121
4.114
3.874
3.863
3.848
3.429
3.410
3.272
3.267
3.251
3.247
0.000
X : parts per Million : Proton

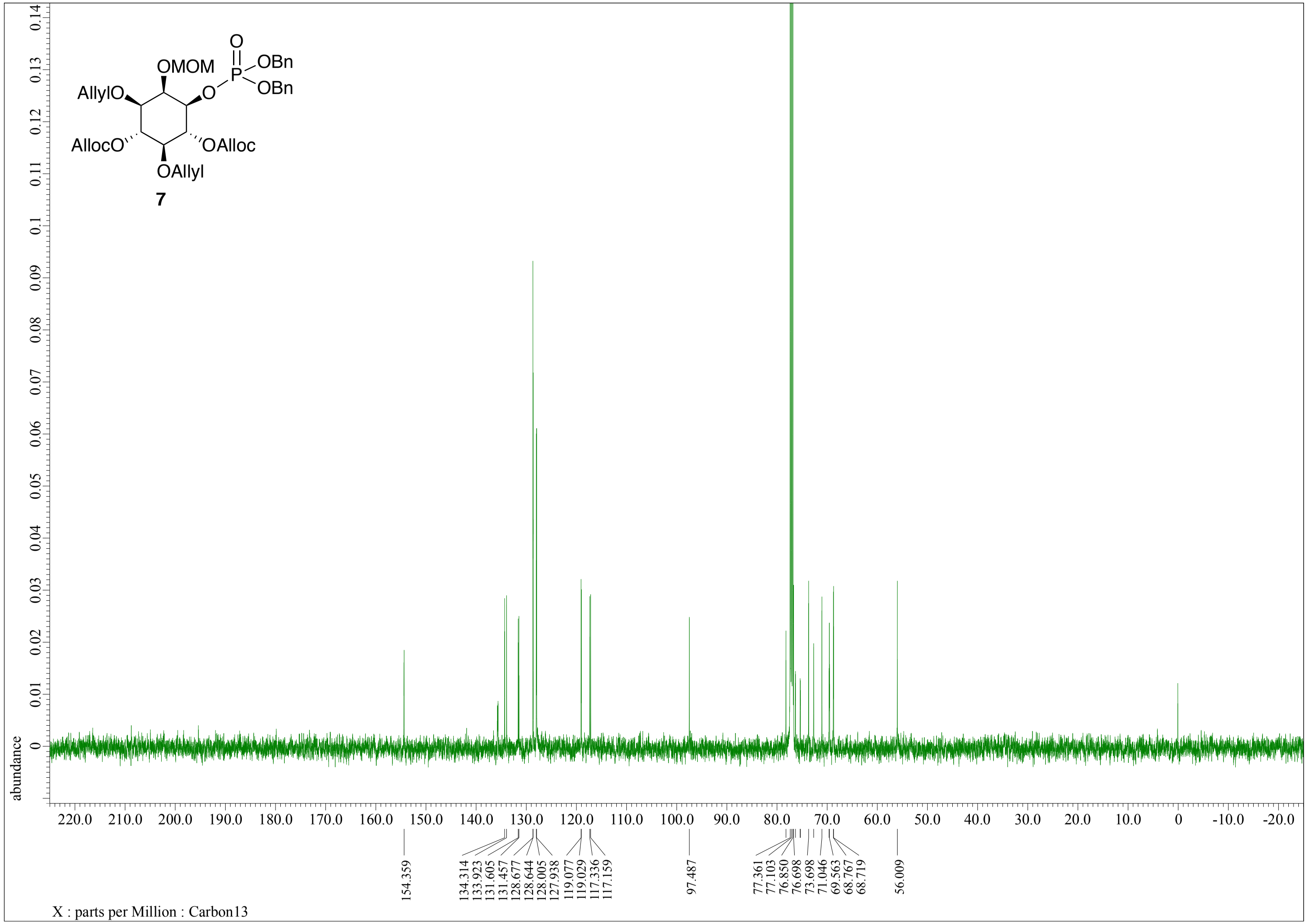
OMOM
AllylO
AllocO
OAlloc
OAllyl
OBn
OBn
7
abundance
0.14
0.13
0.12
0.11
0.1
0.09
0.08
0.07
0.06
0.05
0.04
0.03
0.02
0.01
0
220.0 210.0 200.0 190.0 180.0 170.0 160.0 150.0 140.0 130.0 120.0 110.0 100.0 90.0 80.0 70.0 60.0 50.0 40.0 30.0 20.0 10.0 0 -10.0 -20.0
154.359
134.314
133.923
131.605
131.457
128.677
128.644
128.005
127.938
119.077
119.029
117.336
117.159
97.487
77.361
77.103
76.850
76.698
73.698
71.046
69.563
68.767
68.719
56.009
X : parts per Million : Carbon13

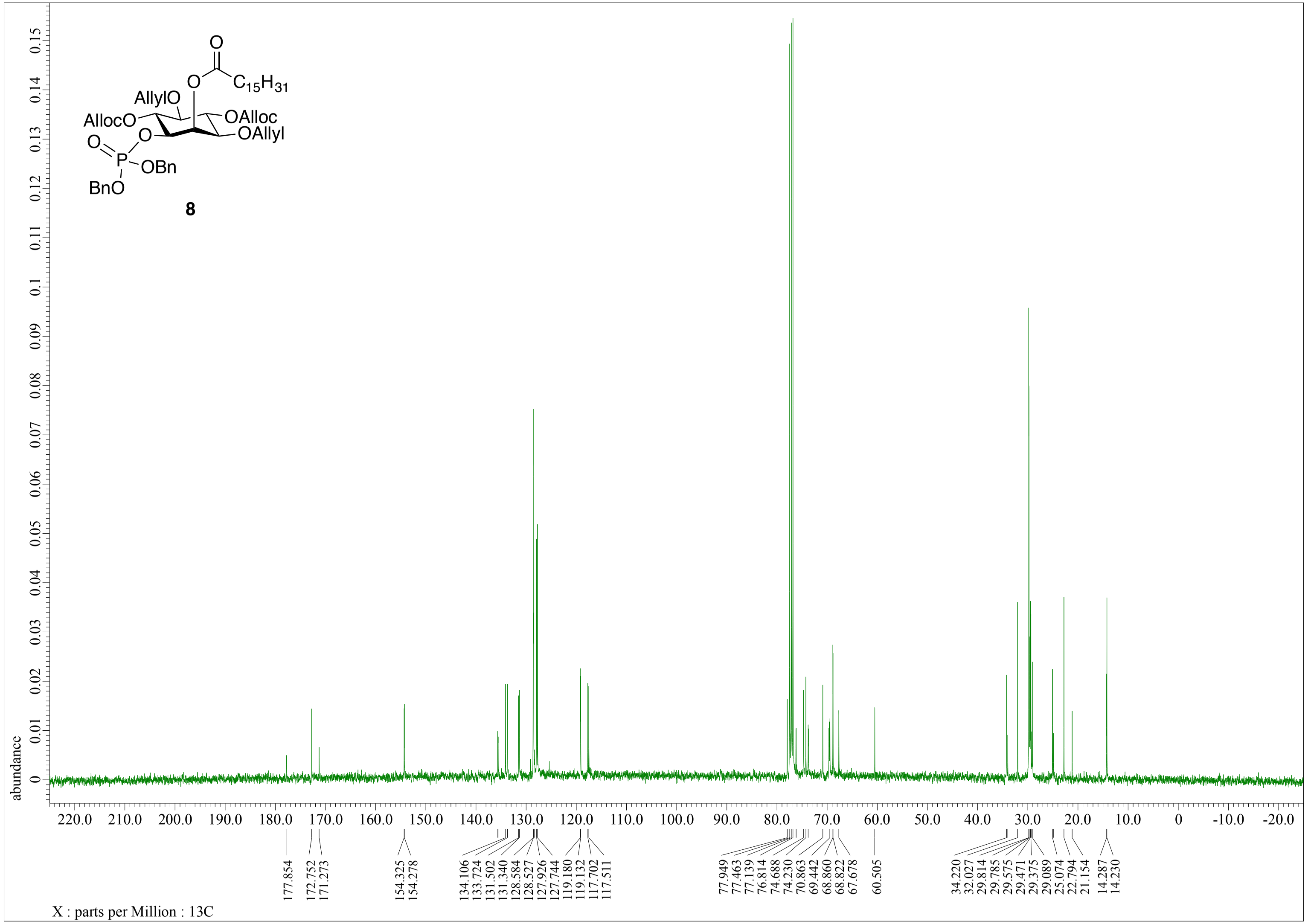

AllocO
AllylO
O
C15H31
OAlloc
OAllyl
OBn
BnO
8
abundance
X : parts per Million : 13C
177.854
172.752
171.273
154.325
154.278
134.106
133.724
131.502
131.340
128.584
128.527
127.926
127.744
119.180
119.132
117.702
117.511
77.949
77.463
77.139
76.814
74.688
74.230
70.863
69.442
68.860
68.822
67.678
60.505
34.220
32.027
29.814
29.785
29.575
29.471
29.375
29.089
25.074
22.794
21.154
14.287
14.230

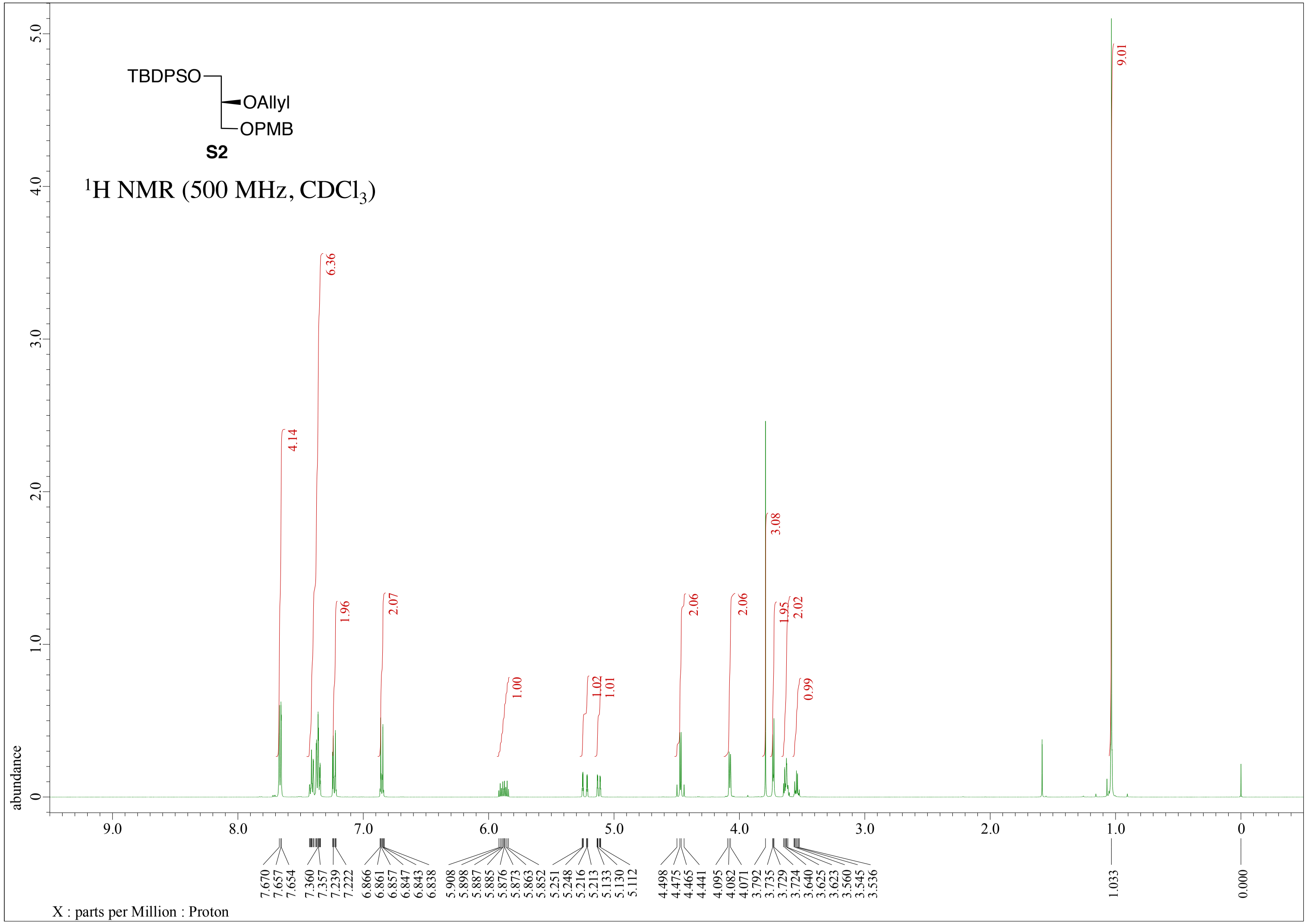
TBDPSO
OAllyl
OPMB
S2
^{1}H NMR (500 MHz, CDCl$_3$)
abundance
X : parts per Million : Proton
4.14
6.36
1.96
2.07
1.00
1.02
1.01
2.06
2.06
3.08
1.95
2.02
0.99
9.01
7.670 7.657 7.654 7.360 7.357 7.239 7.222 6.866 6.861 6.857 6.847 6.843 6.838 5.908 5.898 5.887 5.885 5.876 5.873 5.863 5.852 5.251 5.248 5.216 5.213 5.133 5.130 5.112 4.498 4.475 4.465 4.441 4.095 4.082 4.071 3.792 3.735 3.729 3.724 3.640 3.625 3.623 3.560 3.545 3.536 1.033 0.000

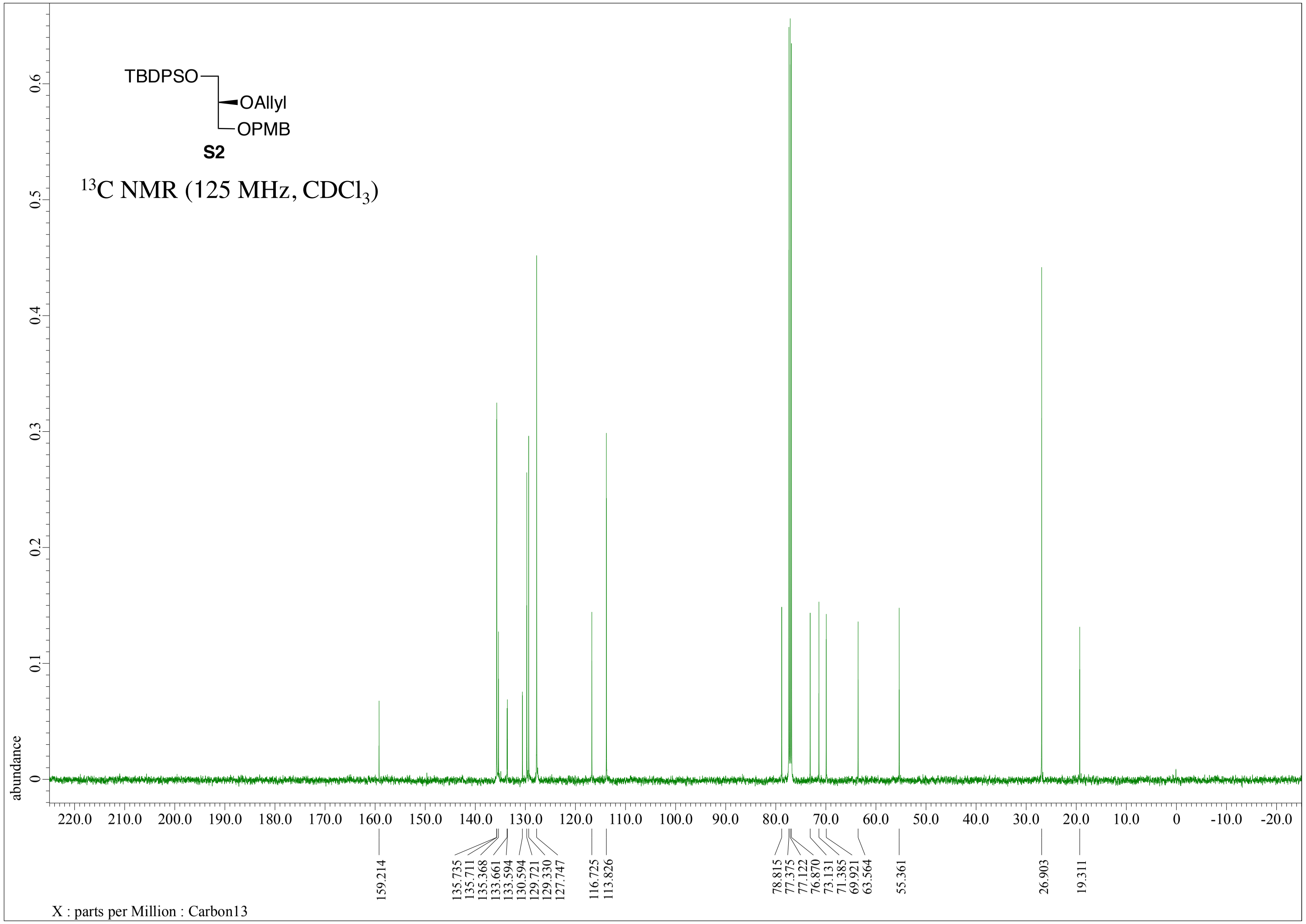
TBDPSO
OAllyl
OPMB
S2
13C NMR (125 MHz, CDCl3)
abundance
0
0.1
0.2
0.3
0.4
0.5
0.6
220.0 210.0 200.0 190.0 180.0 170.0 160.0 150.0 140.0 130.0 120.0 110.0 100.0 90.0 80.0 70.0 60.0 50.0 40.0 30.0 20.0 10.0 0 -10.0 -20.0
159.214
135.735
135.711
135.368
133.661
133.594
130.594
129.721
129.330
127.747
116.725
113.826
78.815
77.375
77.122
76.870
73.131
71.385
69.921
63.564
55.361
26.903
19.311
X : parts per Million : Carbon13

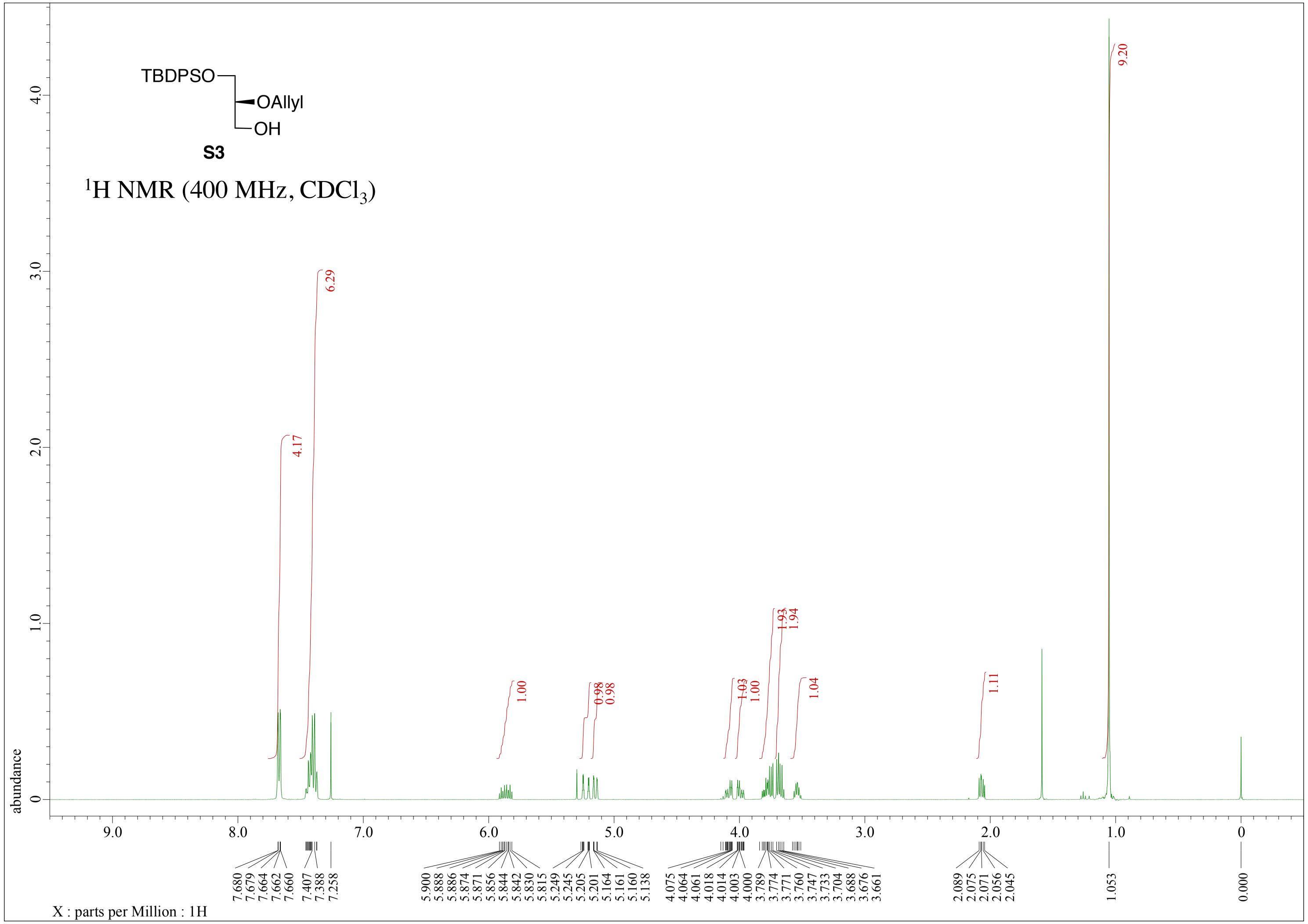
TBDPSO
OAllyl
OH
S3
1H NMR (400 MHz, CDCl3)
abundance
X : parts per Million : 1H
4.17
6.29
1.00
0.98
0.98
1.03
1.00
1.93
1.94
1.04
1.11
9.20
7.680
7.679
7.664
7.662
7.660
7.407
7.388
7.258
5.900
5.888
5.886
5.874
5.871
5.856
5.844
5.842
5.830
5.815
5.249
5.245
5.205
5.201
5.164
5.161
5.160
5.138
4.075
4.064
4.061
4.018
4.014
4.003
4.000
3.789
3.774
3.771
3.760
3.747
3.733
3.704
3.688
3.676
3.661
2.089
2.075
2.071
2.056
2.045
1.053
0.000

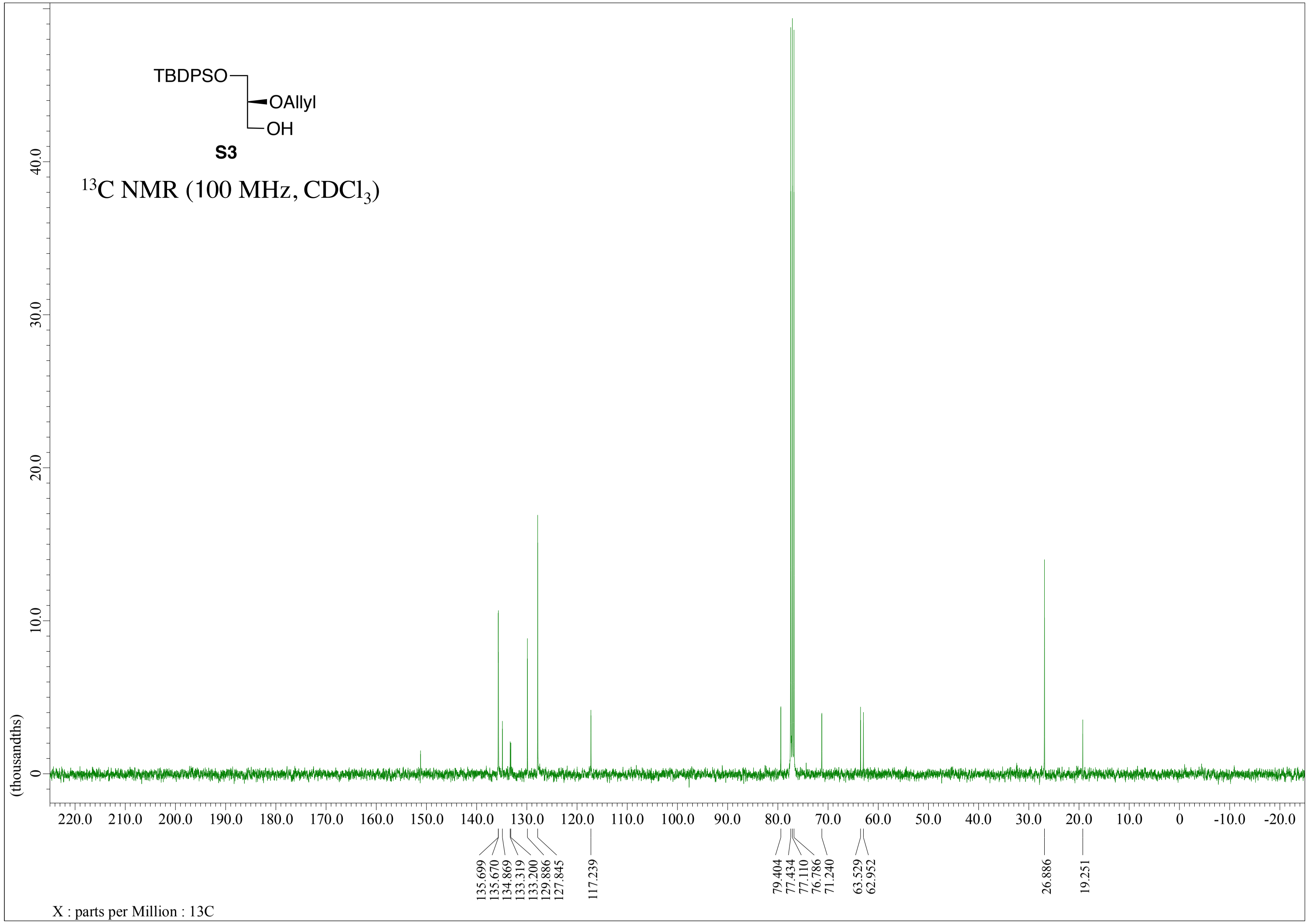
TBDPSO
OAllyl
OH
S3
^{13}C NMR (100 MHz, $CDCl_3$)
(thousandths)
0
10.0
20.0
30.0
40.0
220.0 210.0 200.0 190.0 180.0 170.0 160.0 150.0 140.0 130.0 120.0 110.0 100.0 90.0 80.0 70.0 60.0 50.0 40.0 30.0 20.0 10.0 0 -10.0 -20.0
135.699
135.670
134.869
133.319
133.200
129.886
127.845
117.239
79.404
77.434
77.110
76.786
71.240
63.529
62.952
26.886
19.251
X : parts per Million : 13C

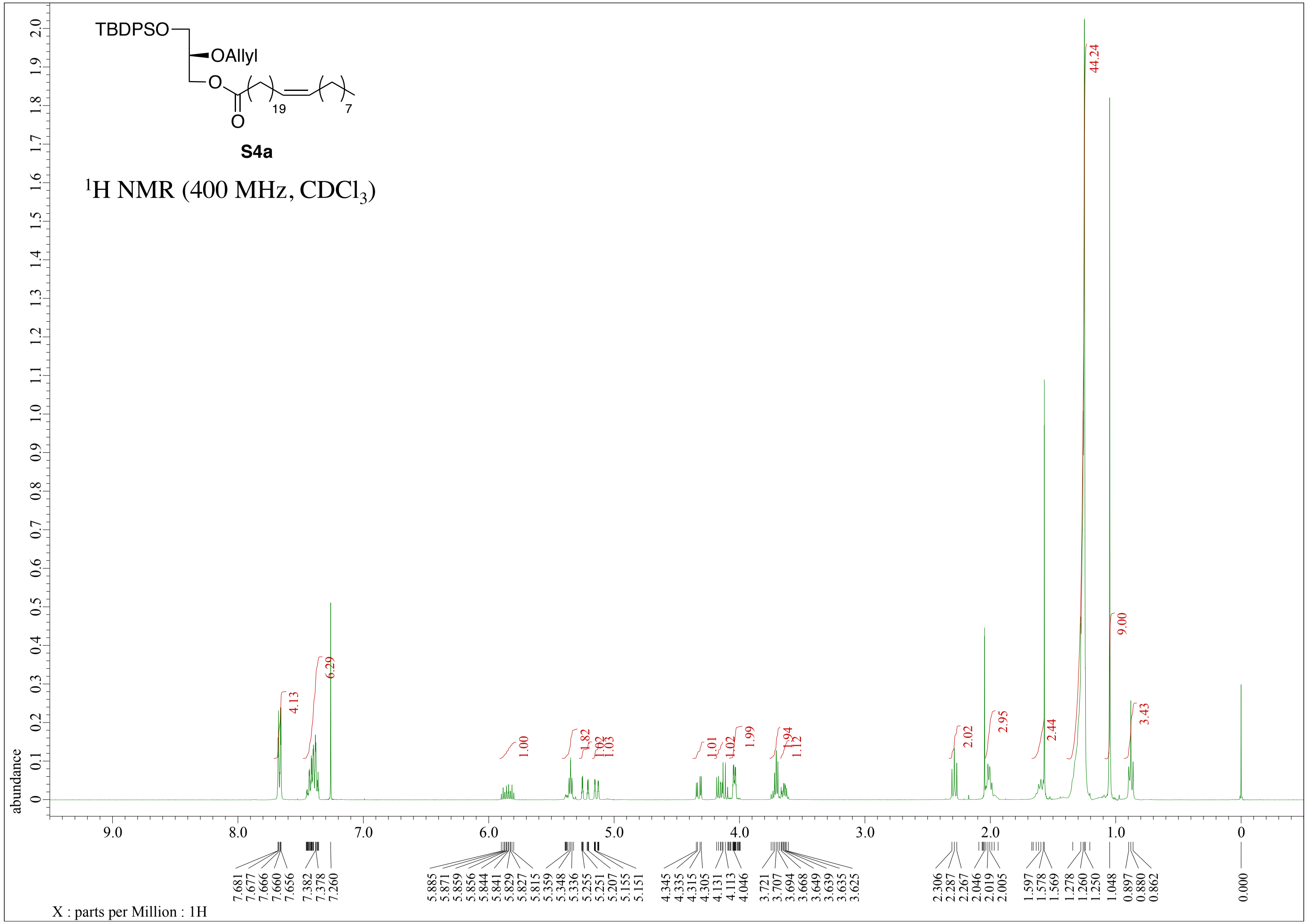
TBDPSO
OAllyl
O
O
19
7
S4a
1H NMR (400 MHz, CDCl3)
abundance
X : parts per Million : 1H
7.681 7.677 7.666 7.660 7.656 7.382 7.378 7.260
5.885 5.871 5.859 5.856 5.844 5.841 5.829 5.827 5.815 5.359 5.348 5.336 5.255 5.251 5.207 5.155 5.151
4.345 4.335 4.315 4.305 4.131 4.113 4.046
3.721 3.707 3.694 3.668 3.649 3.639 3.635 3.625
2.306 2.287 2.267 2.046 2.019 2.005
1.597 1.578 1.569 1.278 1.260 1.250 1.048 0.897 0.880 0.862
0.000
4.13 6.29 1.00 1.82 1.02 1.03 1.01 1.02 1.99 1.94 1.12 2.02 2.95 2.44 44.24 9.00 3.43

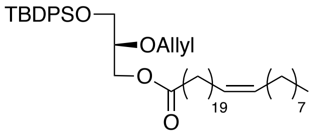

S4a

^{13}C NMR (100 MHz, $CDCl_3$)

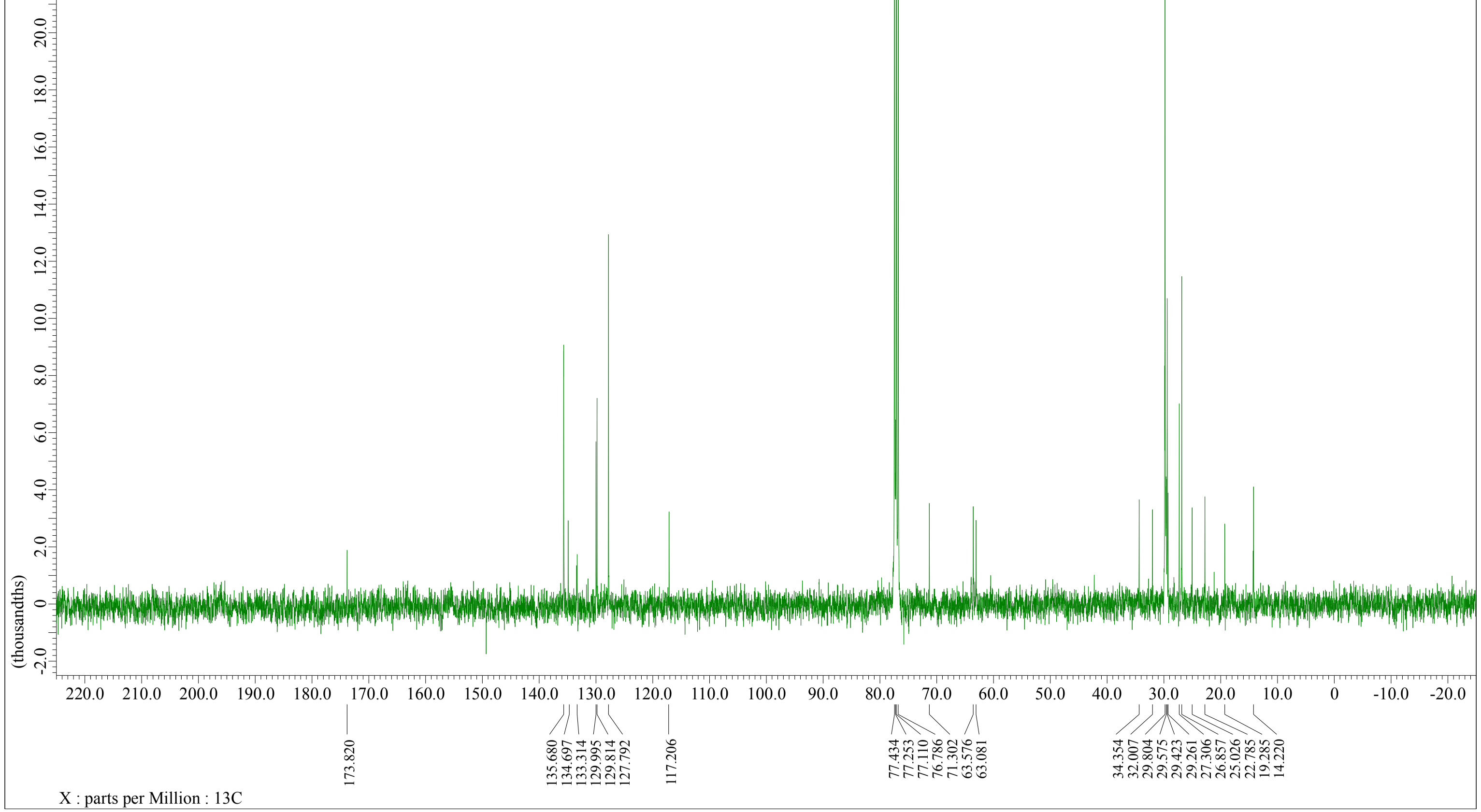

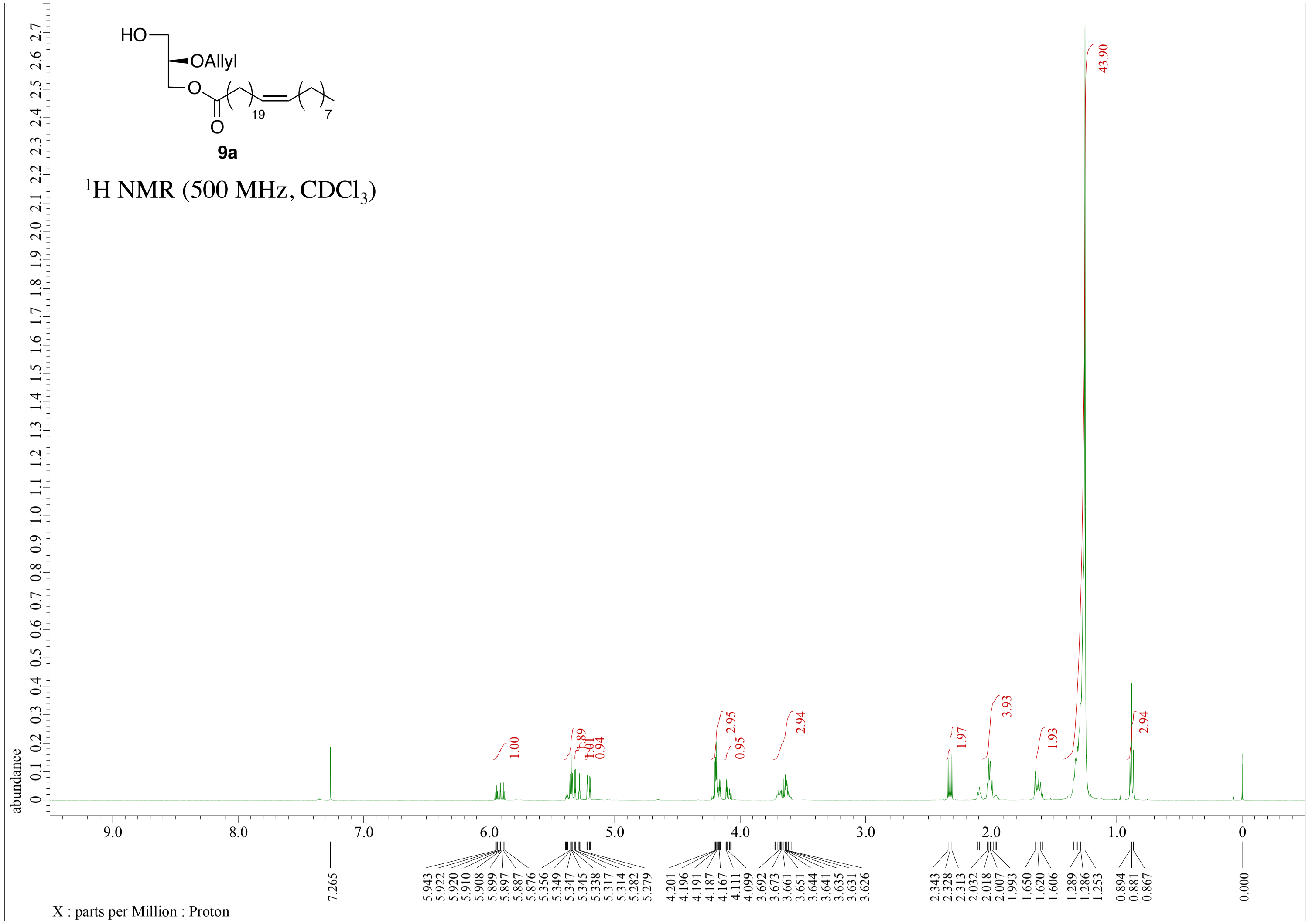

HO
OAllyl
O
O
19
7
9a
^{1}H NMR (500 MHz, $CDCl_3$)
abundance
X : parts per Million : Proton
1.00
1.89
1.01
0.94
2.95
0.95
2.94
1.97
3.93
1.93
43.90
2.94
7.265
5.943 5.922 5.920 5.910 5.908 5.899 5.897 5.887 5.876
5.356 5.349 5.347 5.345 5.338 5.317 5.314 5.282 5.279
4.201 4.196 4.191 4.187 4.167 4.111 4.099
3.692 3.673 3.661 3.651 3.644 3.641 3.635 3.631 3.626
2.343 2.328 2.313
2.032 2.018 2.007 1.993
1.650 1.620 1.606
1.289 1.286 1.253
0.894 0.881 0.867
0.000

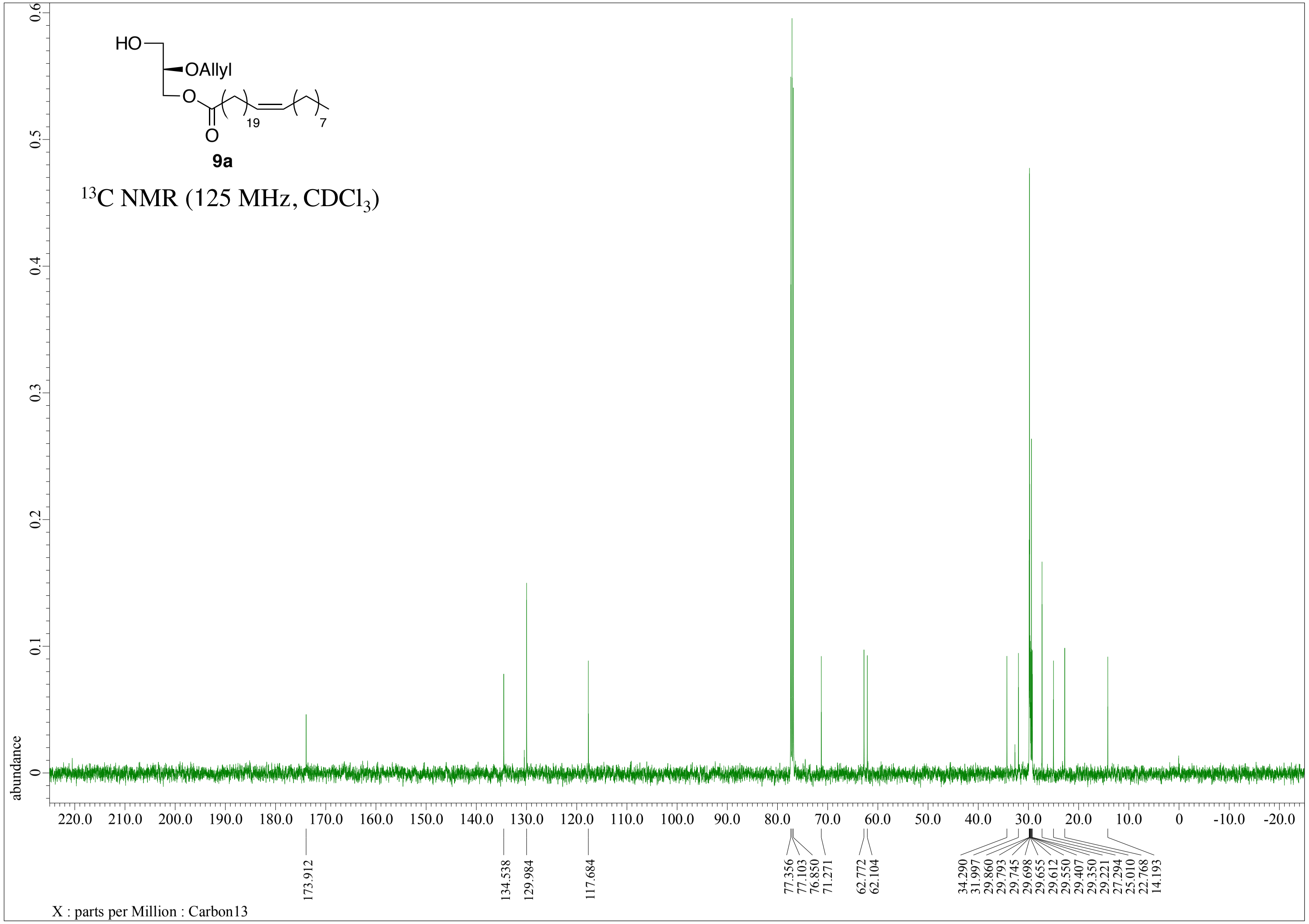
HO
OAllyl
O
O
19
7
9a
13C NMR (125 MHz, CDCl3)
abundance
0.6
0.5
0.4
0.3
0.2
0.1
0
220.0 210.0 200.0 190.0 180.0 170.0 160.0 150.0 140.0 130.0 120.0 110.0 100.0 90.0 80.0 70.0 60.0 50.0 40.0 30.0 20.0 10.0 0 -10.0 -20.0
173.912
134.538
129.984
117.684
77.356
77.103
76.850
71.271
62.772
62.104
34.290
31.997
29.860
29.793
29.745
29.698
29.655
29.612
29.550
29.407
29.350
29.221
27.294
25.010
22.768
14.193
X : parts per Million : Carbon13

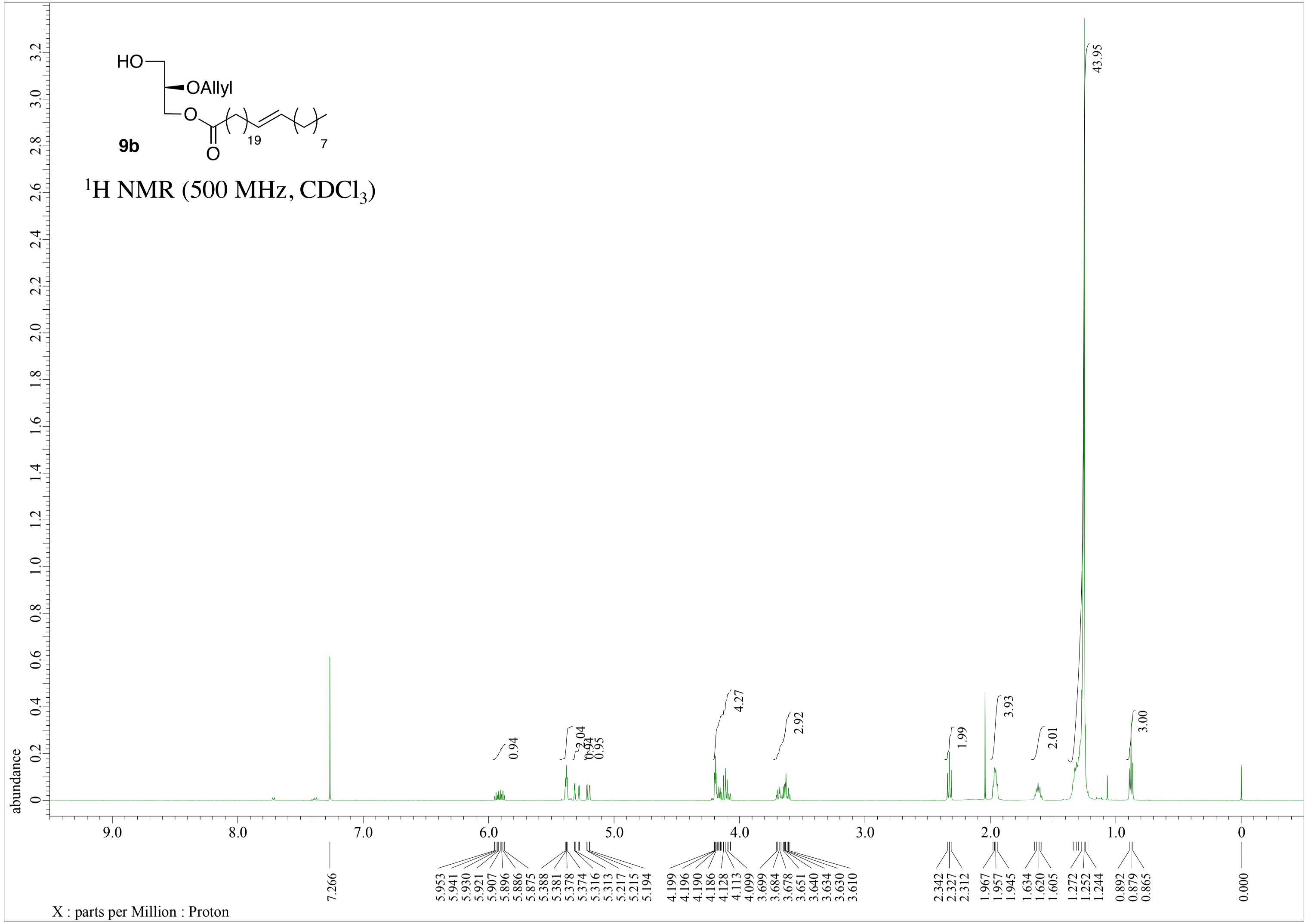

HO
OAllyl
O
19
7
O
9b
1H NMR (500 MHz, CDCl3)
abundance
X : parts per Million : Proton
43.95
4.27
3.93
3.00
2.92
2.01
2.04
1.99
0.94
0.94
0.95
7.266
5.953 5.941 5.930 5.921 5.907 5.896 5.886 5.875
5.388 5.381 5.378 5.374 5.316 5.313 5.217 5.215 5.194
4.199 4.196 4.190 4.186 4.128 4.113 4.099
3.699 3.684 3.678 3.651 3.640 3.634 3.630 3.610
2.342 2.327 2.312
1.967 1.957 1.945
1.634 1.620 1.605
1.272 1.252 1.244
0.892 0.879 0.865
0.000

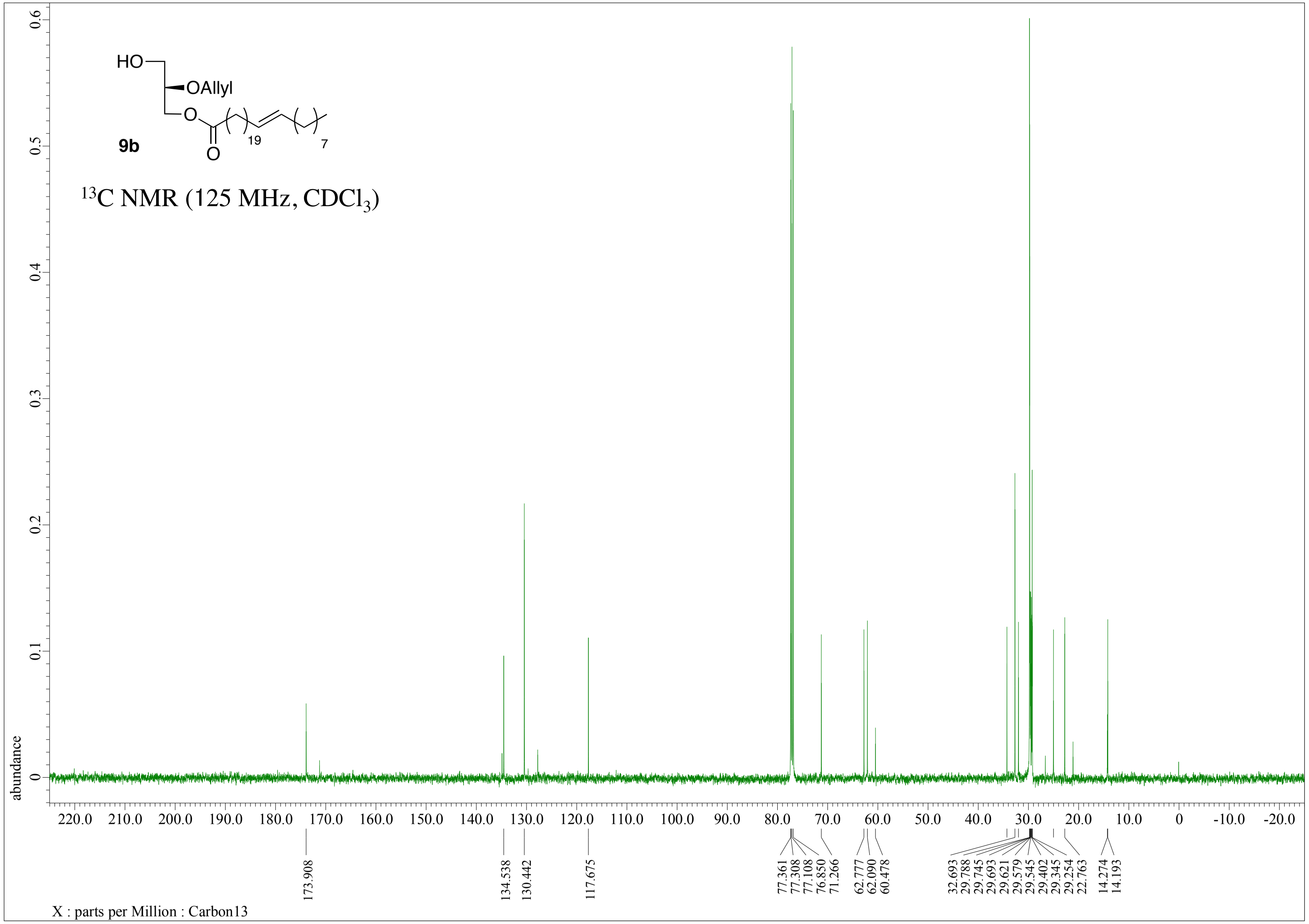
HO
OAllyl
19
7
9b
^{13}C NMR (125 MHz, $CDCl_3$)
abundance
173.908
134.538
130.442
117.675
77.361
77.308
77.108
76.850
71.266
62.777
62.090
60.478
32.693
29.788
29.745
29.693
29.621
29.579
29.545
29.402
29.345
29.254
22.763
14.274
14.193
X : parts per Million : Carbon13

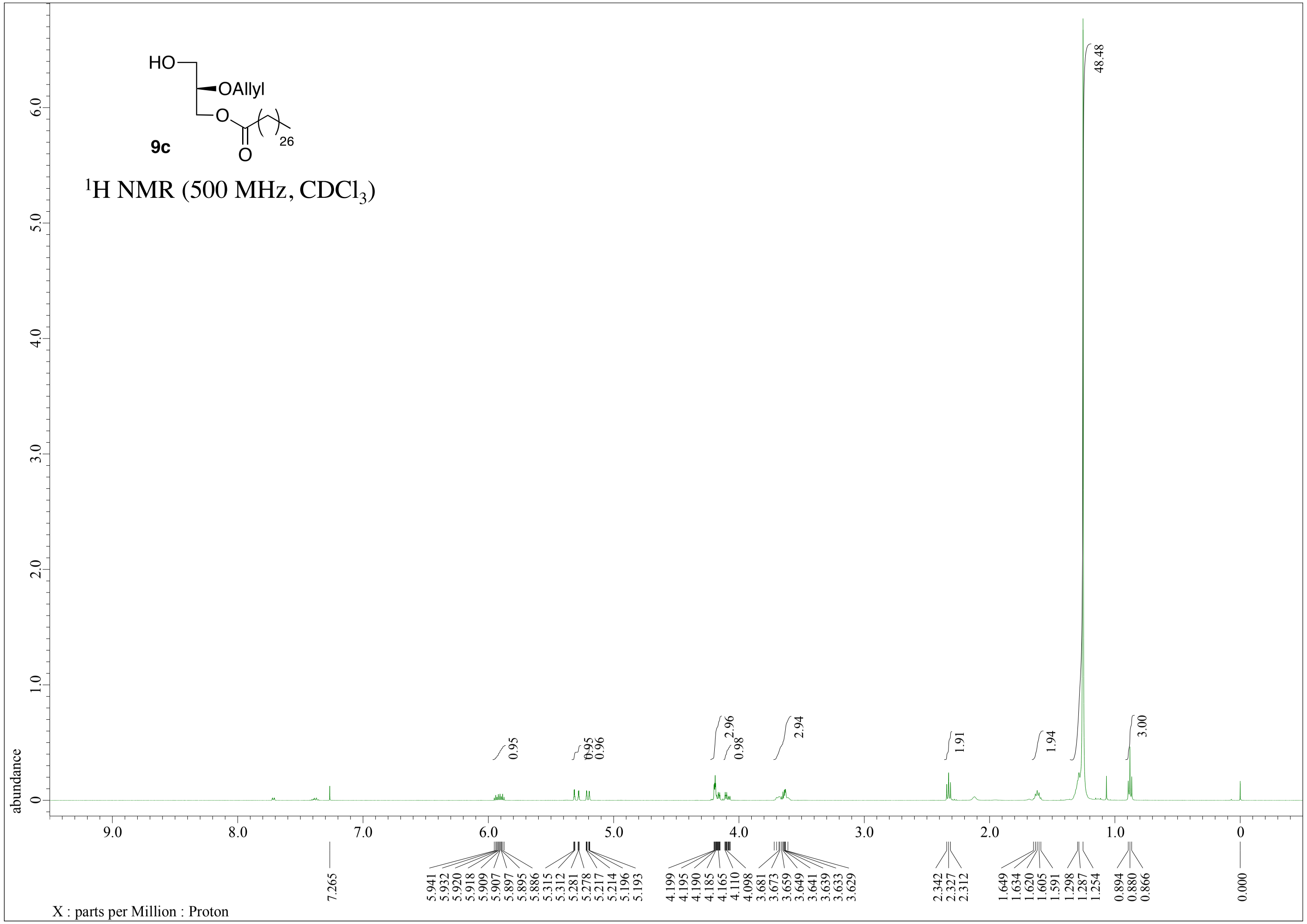
HO
OAllyl
O
O
26
9c
^{1}H NMR (500 MHz, CDCl$_3$)
48.48
3.00
1.94
1.91
2.94
2.96
0.98
0.95
0.96
0.95
abundance
0
1.0
2.0
3.0
4.0
5.0
6.0
9.0
8.0
7.0
6.0
5.0
4.0
3.0
2.0
1.0
0
7.265
5.941 5.932 5.920 5.918 5.909 5.907 5.897 5.895 5.886
5.315 5.312 5.281 5.278 5.217 5.214 5.196 5.193
4.199 4.195 4.190 4.185 4.165 4.110 4.098
3.681 3.673 3.659 3.649 3.641 3.639 3.633 3.629
2.342 2.327 2.312
1.649 1.634 1.620 1.605 1.591
1.298 1.287 1.254
0.894 0.880 0.866
0.000
X : parts per Million : Proton

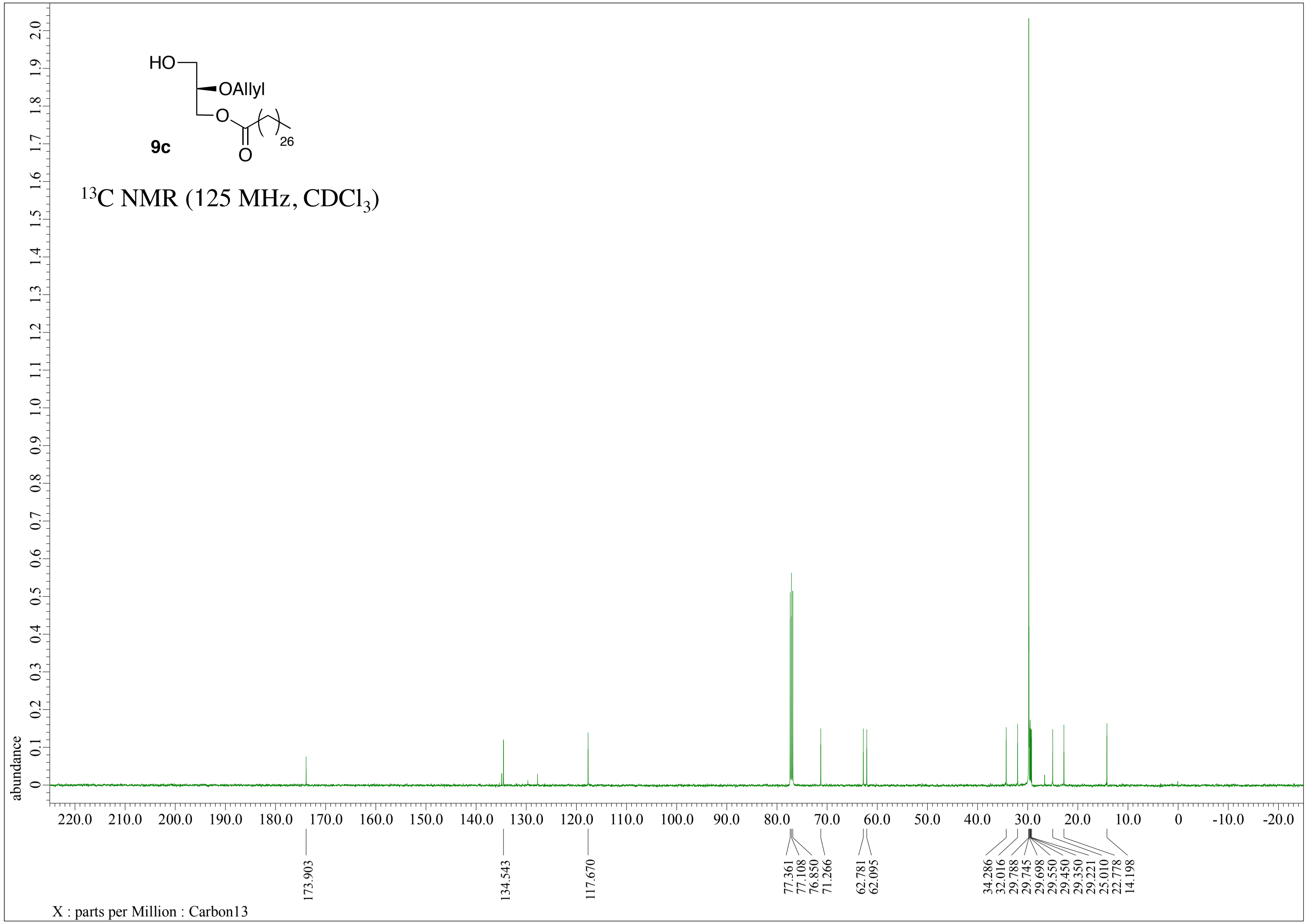
HO
OAllyl
9c
26
O
^{13}C NMR (125 MHz, CDCl$_3$)
abundance
173.903
134.543
117.670
77.361
77.108
76.850
71.266
62.781
62.095
34.286
32.016
29.788
29.745
29.698
29.550
29.450
29.350
29.221
25.010
22.778
14.198
X : parts per Million : Carbon13

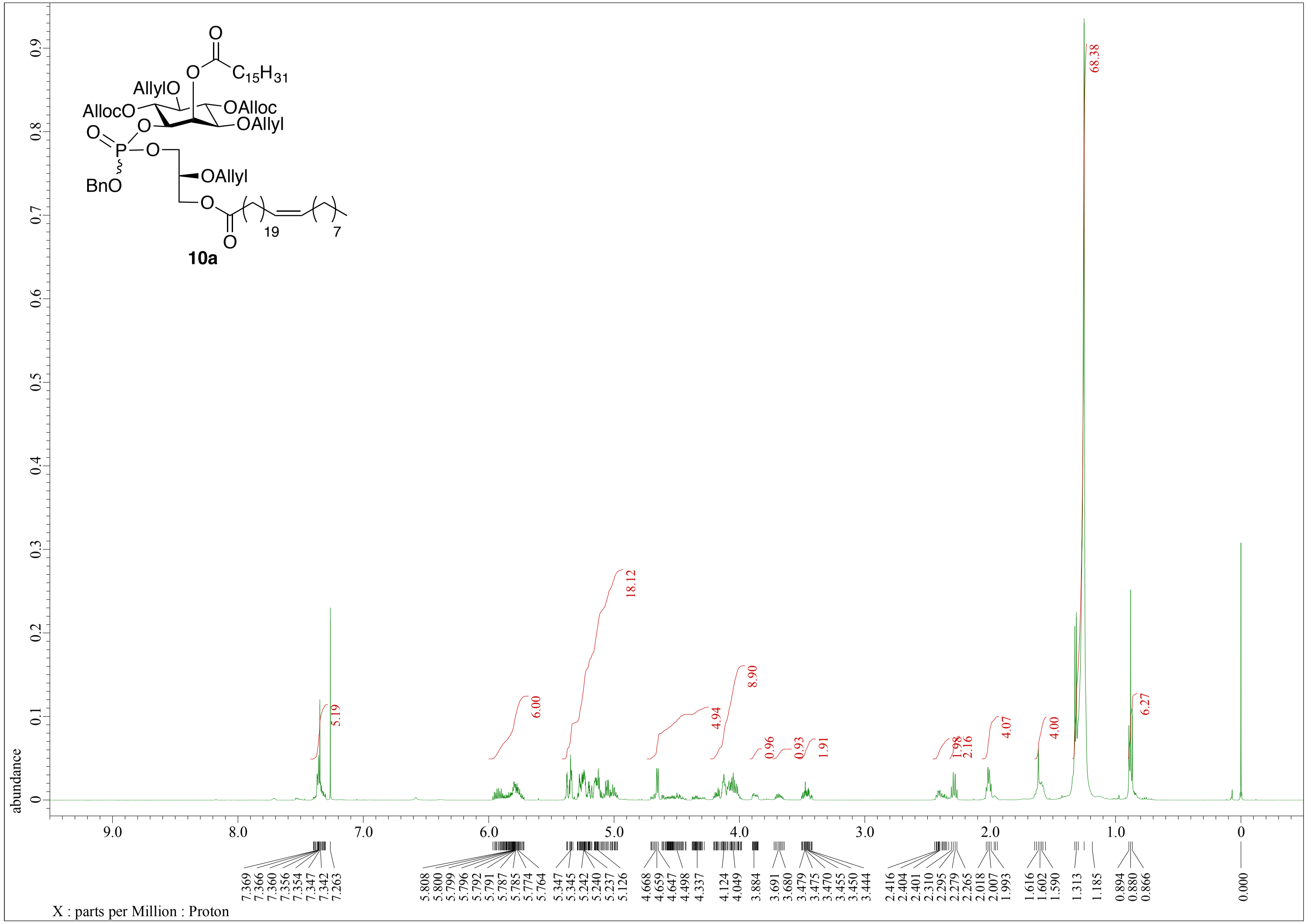

AllocO
AllylO
O
C15H31
OAlloc
OAllyl
O
P
BnO
OAllyl
19
7
10a
abundance
X : parts per Million : Proton

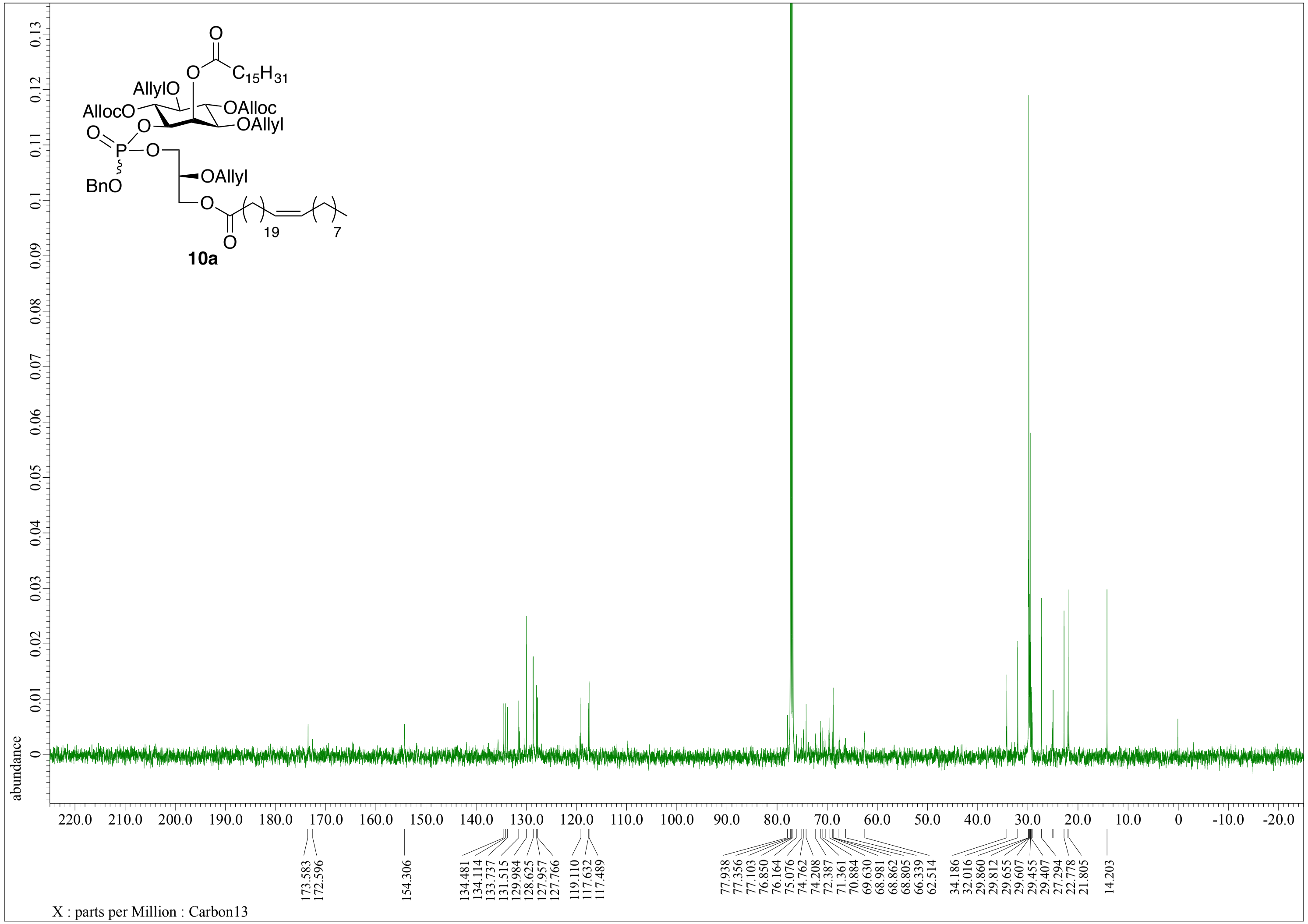
10a
AllocO
AllylO
OAlloc
OAllyl
BnO
OAllyl
C15H31
19
7
abundance
X : parts per Million : Carbon13
173.583
172.596
154.306
134.481
134.114
133.737
131.515
129.984
128.625
127.957
127.766
119.110
117.632
117.489
77.938
77.356
77.103
76.850
76.164
75.076
74.762
74.208
72.387
71.361
70.884
69.630
68.981
68.862
68.805
66.339
62.514
34.186
32.016
29.860
29.812
29.655
29.607
29.455
29.407
27.294
22.778
21.805
14.203

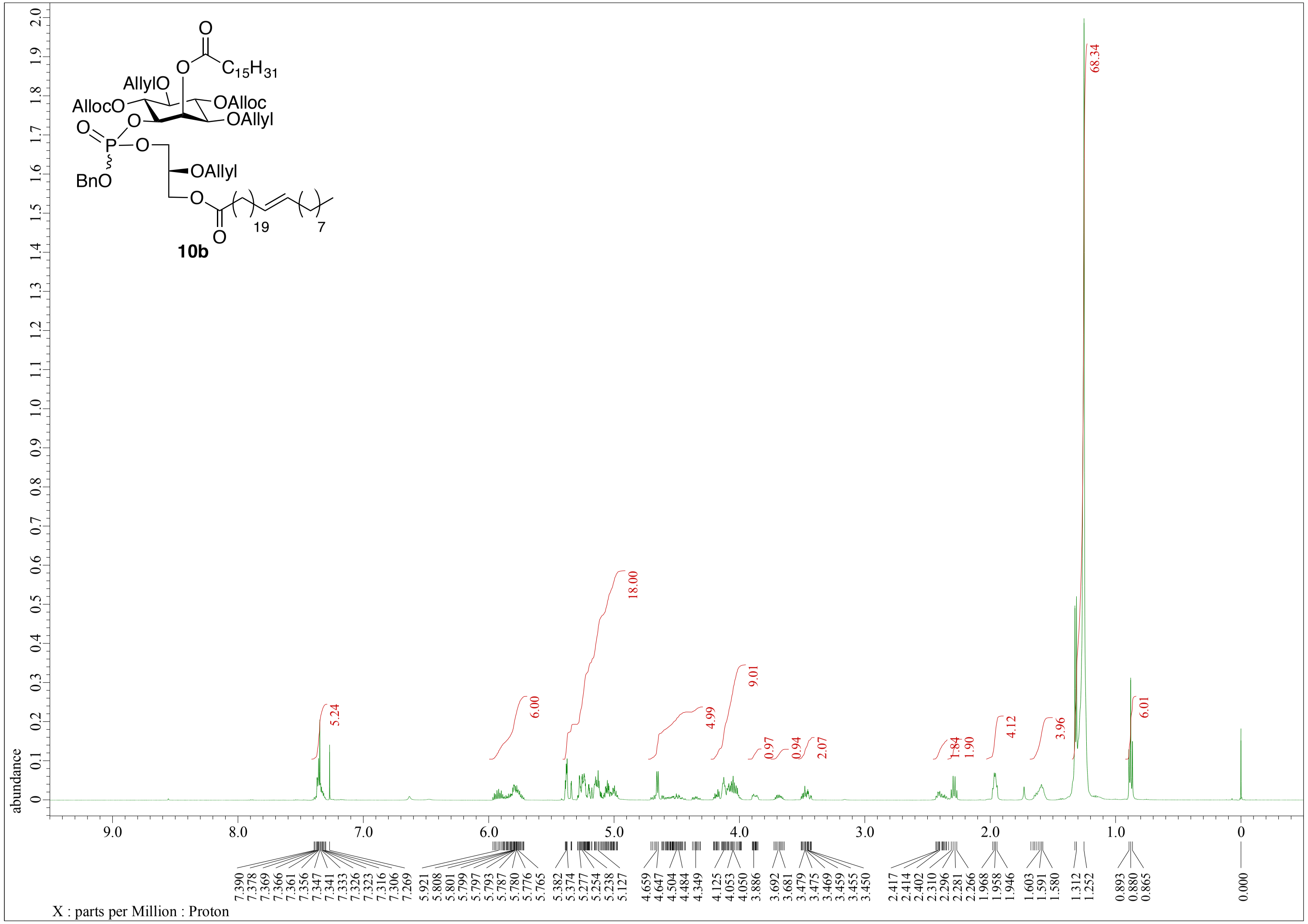

abundance
X : parts per Million : Proton
10b
AllylO
AllocO
OAlloc
OAllyl
C15H31
BnO
OAllyl
19
7
5.24
6.00
18.00
4.99
9.01
0.97
0.94
2.07
1.84
1.90
4.12
3.96
68.34
6.01
7.390 7.378 7.369 7.366 7.361 7.356 7.347 7.341 7.333 7.326 7.323 7.316 7.306 7.269
5.921 5.808 5.801 5.799 5.797 5.793 5.787 5.780 5.776 5.765
5.382 5.374 5.277 5.254 5.238 5.127
4.659 4.647 4.504 4.484 4.349
4.125 4.053 4.050 3.886
3.692 3.681 3.479 3.475 3.469 3.459 3.455 3.450
2.417 2.414 2.402 2.310 2.296 2.281 2.266
1.968 1.958 1.946
1.603 1.591 1.580
1.312 1.252
0.893 0.880 0.865
0.000

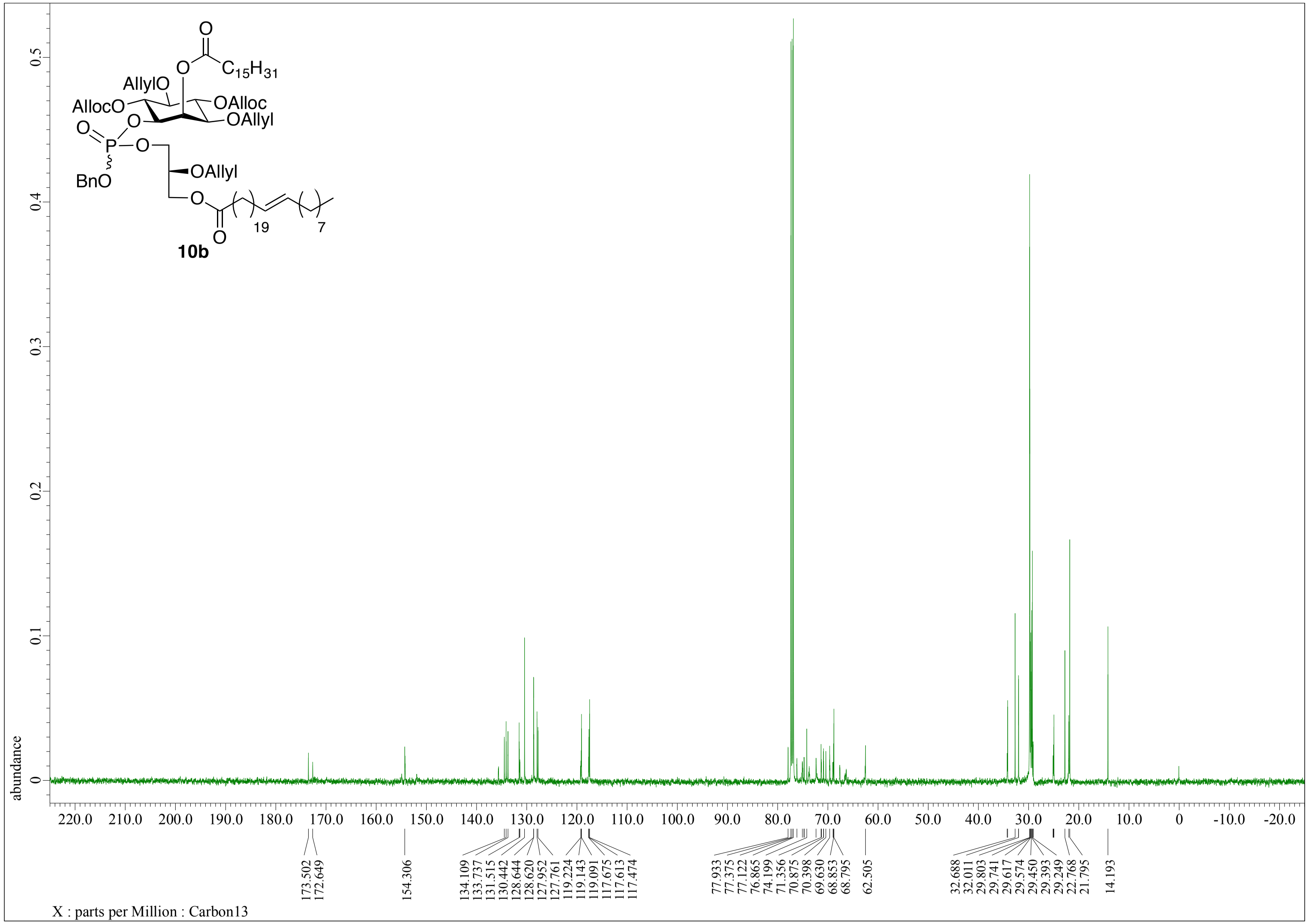

10b
AllocO
AllylO
OAlloc
OAllyl
BnO
OAllyl
$C_{15}H_{31}$
19
7
abundance
0.5
0.4
0.3
0.2
0.1
0
220.0 210.0 200.0 190.0 180.0 170.0 160.0 150.0 140.0 130.0 120.0 110.0 100.0 90.0 80.0 70.0 60.0 50.0 40.0 30.0 20.0 10.0 0 -10.0 -20.0
173.502
172.649
154.306
134.109
133.737
131.515
130.442
128.644
128.620
127.952
127.761
119.224
119.143
119.091
117.675
117.613
117.474
77.933
77.375
77.122
76.865
74.199
71.356
70.875
70.398
69.630
68.853
68.795
62.505
32.688
32.011
29.803
29.741
29.617
29.574
29.450
29.393
29.249
22.768
21.795
14.193
X : parts per Million : Carbon13

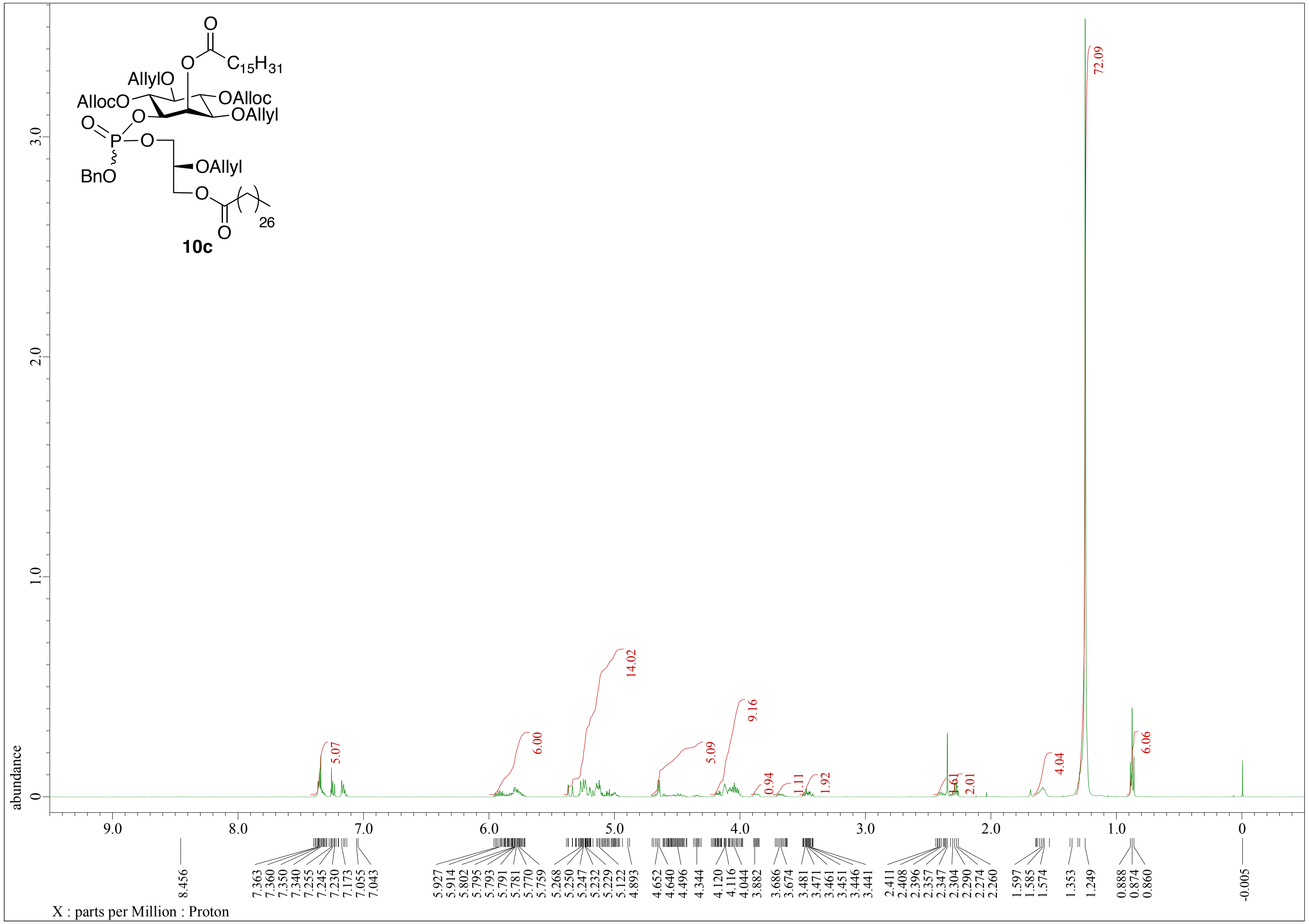

AllylO
AllocO
OAlloc
OAllyl
BnO
OAllyl
26
10c
abundance
X : parts per Million : Proton

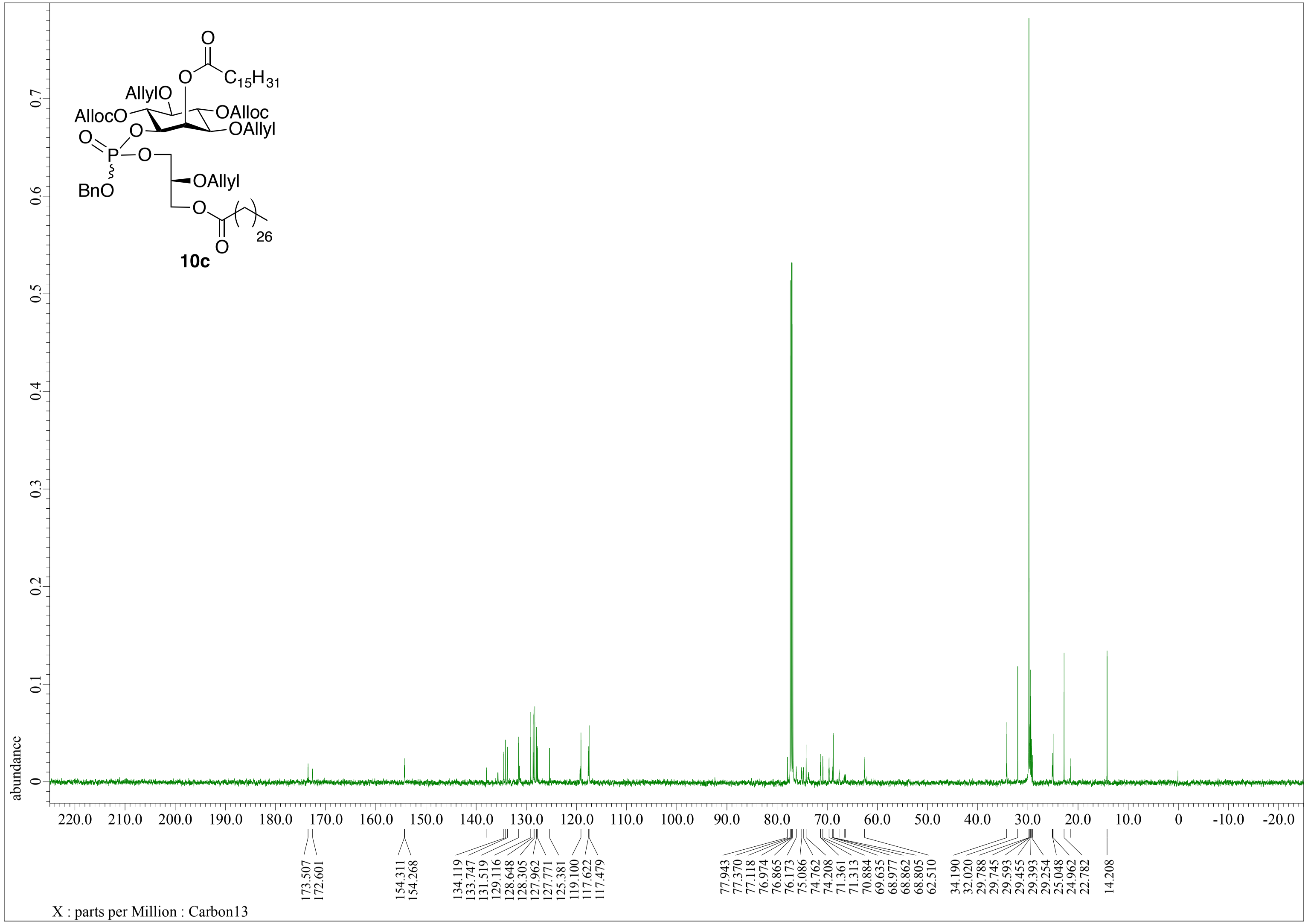

AllocO
AllylO
O
C15H31
OAlloc
OAllyl
P
BnO
OAllyl
26
10c
abundance
0.7
0.6
0.5
0.4
0.3
0.2
0.1
0
220.0 210.0 200.0 190.0 180.0 170.0 160.0 150.0 140.0 130.0 120.0 110.0 100.0 90.0 80.0 70.0 60.0 50.0 40.0 30.0 20.0 10.0 0 -10.0 -20.0
173.507
172.601
154.311
154.268
134.119
133.747
131.519
129.116
128.648
128.305
127.962
127.771
125.381
119.100
117.622
117.479
77.943
77.370
77.118
76.974
76.865
76.173
75.086
74.762
74.208
71.361
71.313
70.884
69.635
68.977
68.862
68.805
62.510
34.190
32.020
29.788
29.745
29.593
29.455
29.393
29.254
25.048
24.962
22.782
14.208
X : parts per Million : Carbon13

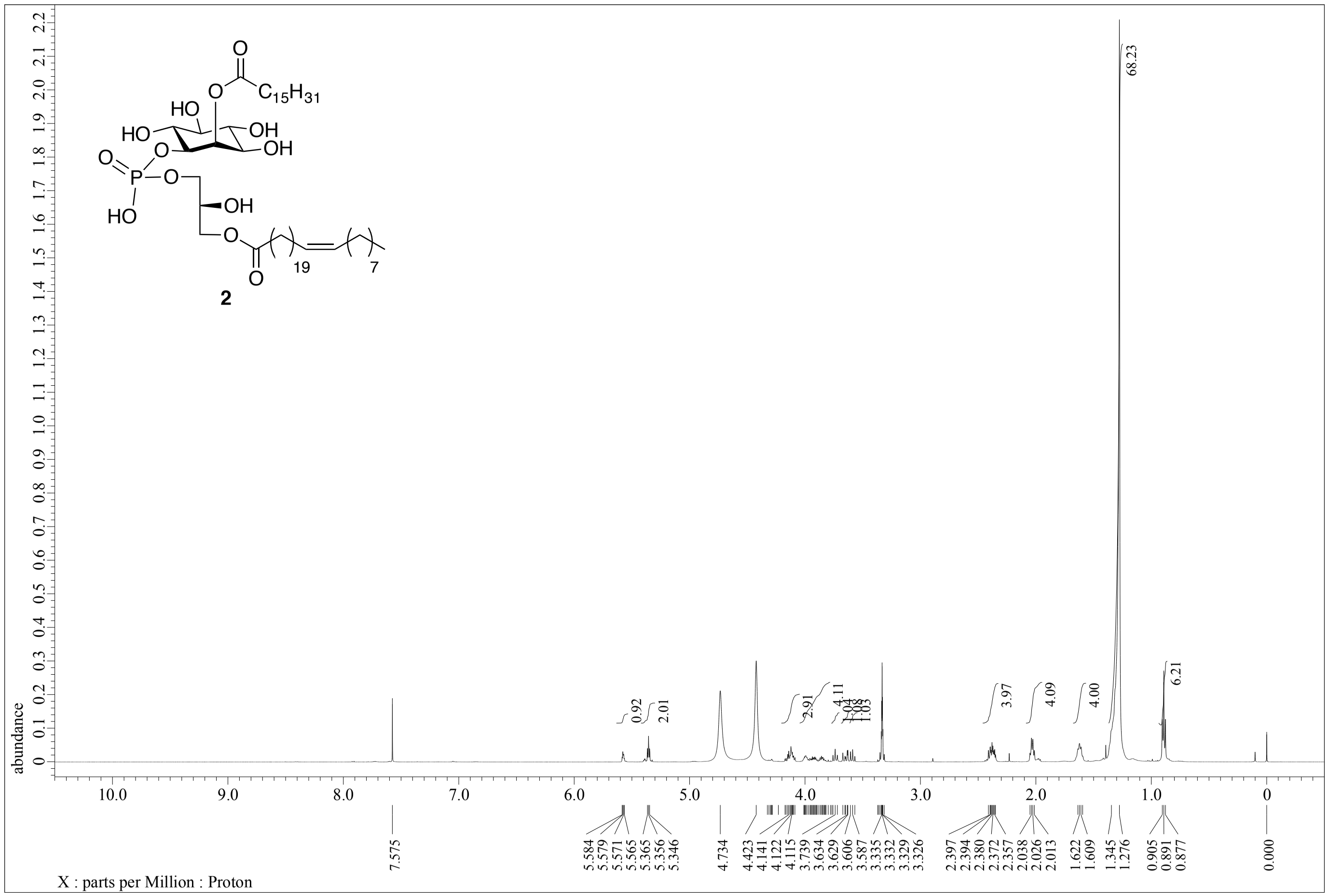

abundance
2.2
2.1
2.0
1.9
1.8
1.7
1.6
1.5
1.4
1.3
1.2
1.1
1.0
0.9
0.8
0.7
0.6
0.5
0.4
0.3
0.2
0.1
0
O
O
$C_{15}H_{31}$
HO
HO
OH
OH
O
P
O
O
HO
OH
O
O
19
7
0.92
2.01
2.91
4.11
1.04
1.08
1.03
3.97
4.09
4.00
68.23
6.21
10.0
9.0
8.0
7.0
6.0
5.0
4.0
3.0
2.0
1.0
0
7.575
5.584
5.579
5.571
5.565
5.365
5.356
5.346
4.734
4.423
4.141
4.122
4.115
3.739
3.634
3.629
3.606
3.587
3.335
3.332
3.329
3.326
2.397
2.394
2.380
2.372
2.357
2.038
2.026
2.013
1.622
1.609
1.345
1.276
0.905
0.891
0.877
0.000
X : parts per Million : Proton

2

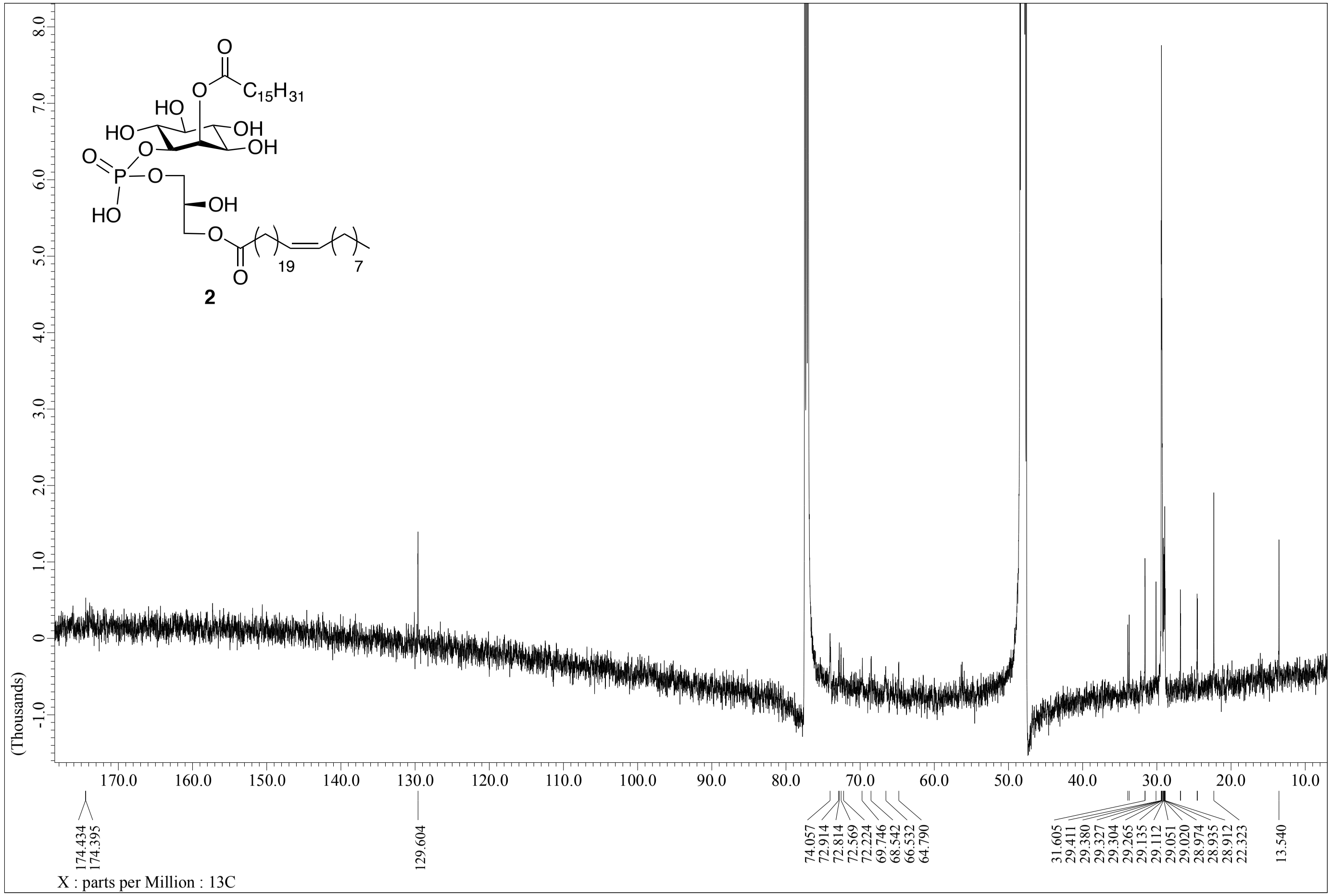
O
C15H31
HO
HO
OH
OH
O
O
P
O
HO
OH
O
19
7
O
8.0
7.0
6.0
5.0
4.0
3.0
2.0
1.0
0
-1.0
(Thousands)
170.0
160.0
150.0
140.0
130.0
120.0
110.0
100.0
90.0
80.0
70.0
60.0
50.0
40.0
30.0
20.0
10.0
174.434
174.395
129.604
74.057
72.914
72.814
72.569
72.224
69.746
68.542
66.532
64.790
31.605
29.411
29.380
29.327
29.304
29.265
29.135
29.112
29.051
29.020
28.974
28.935
28.912
22.323
13.540
X : parts per Million : 13C

2

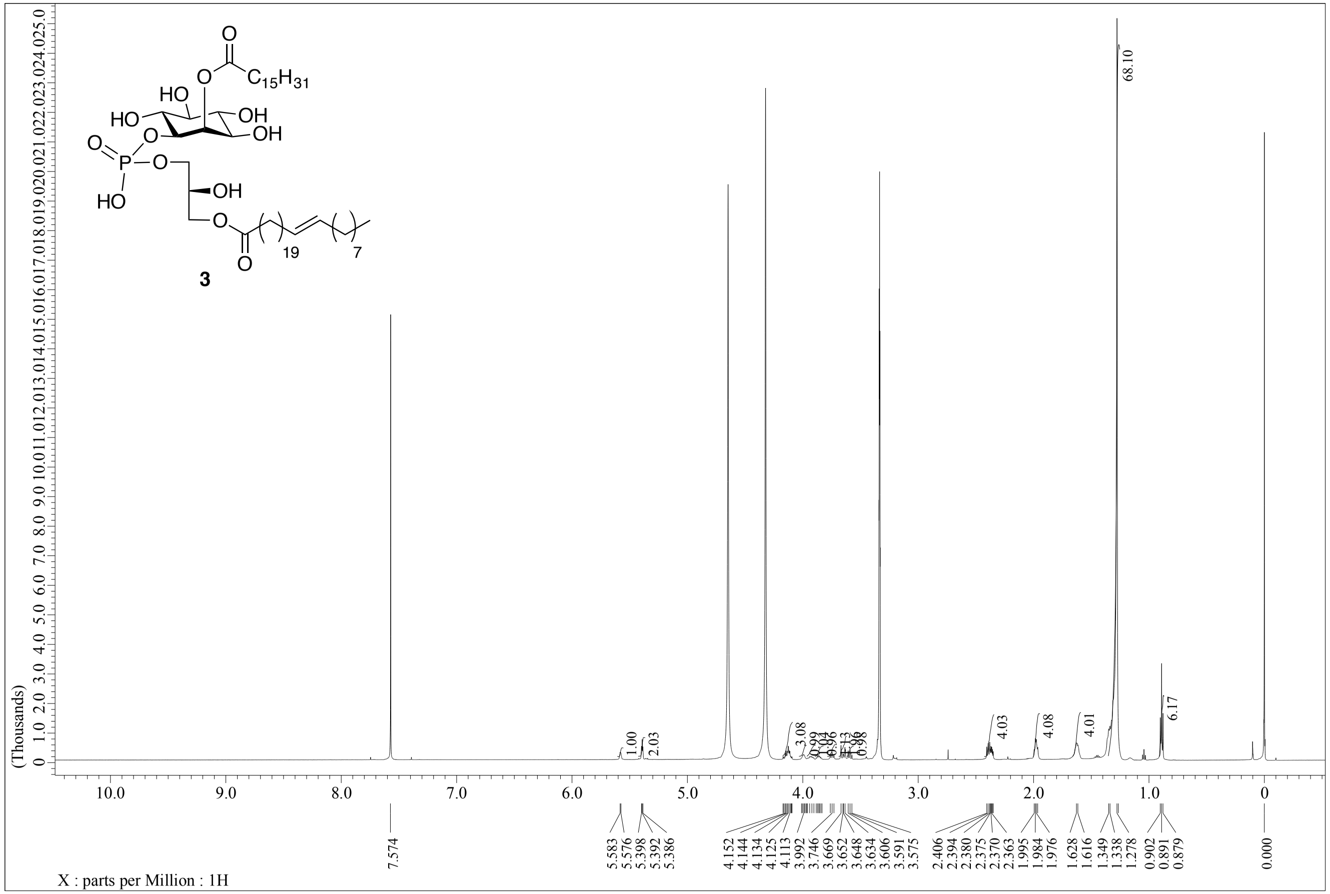
(Thousands)
X : parts per Million : 1H
3
7.574
5.583 5.576 5.398 5.392 5.386
4.152 4.144 4.134 4.125 4.113 3.992 3.746 3.669 3.652 3.648 3.634 3.606 3.591 3.575
2.406 2.394 2.380 2.375 2.370 2.363 1.995 1.984 1.976
1.628 1.616 1.349 1.338 1.278 0.902 0.891 0.879
0.000
1.00 2.03 3.08 4.03 4.08 4.01 68.10 6.17

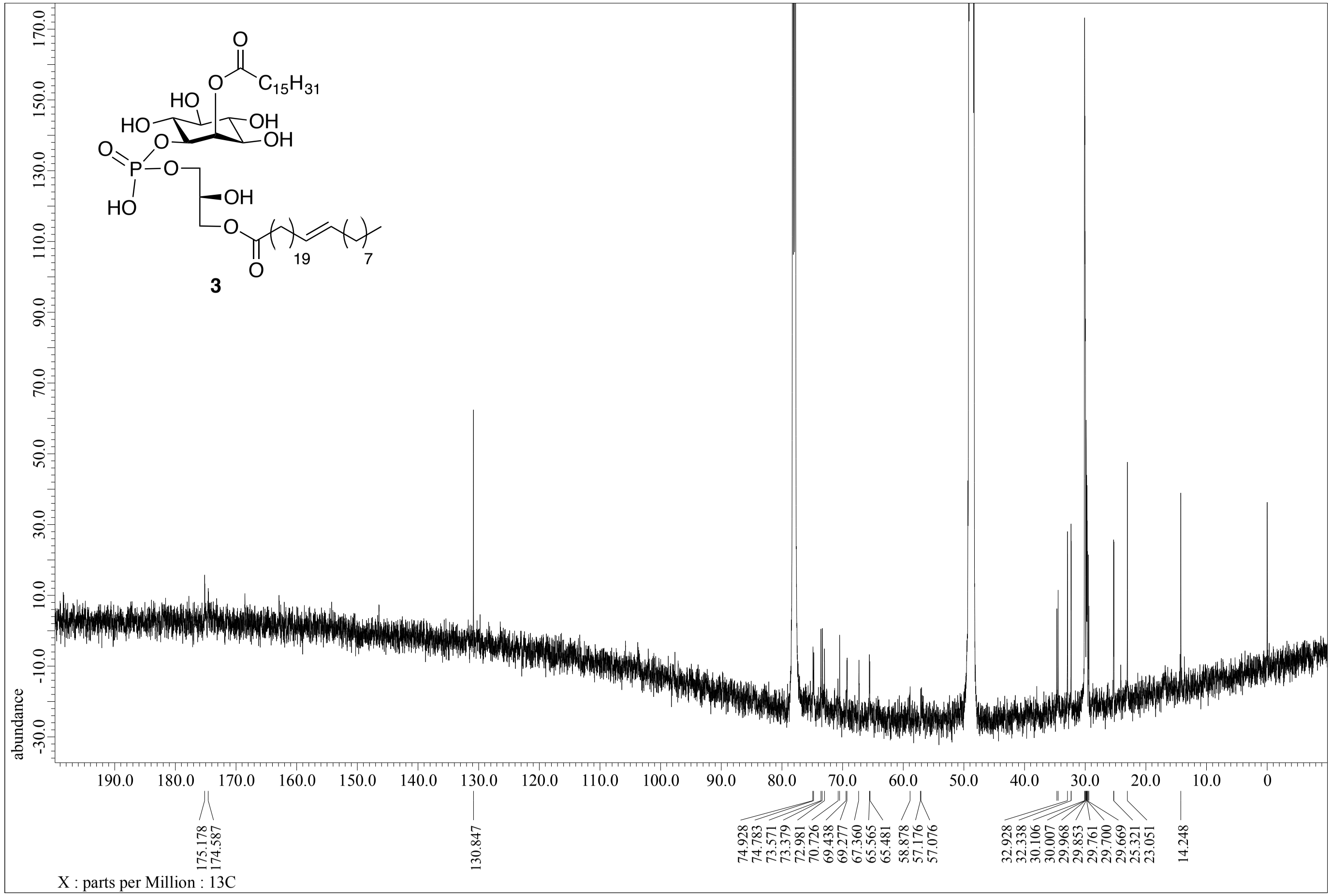

O
$C_{15}H_{31}$
HO
HO
OH
OH
O
P
O
HO
OH
O
19
7
3
abundance
170.0
150.0
130.0
110.0
90.0
70.0
50.0
30.0
10.0
-10.0
-30.0
190.0
180.0
170.0
160.0
150.0
140.0
130.0
120.0
110.0
100.0
90.0
80.0
70.0
60.0
50.0
40.0
30.0
20.0
10.0
0
175.178
174.587
130.847
74.928
74.783
73.571
73.379
72.981
70.726
69.438
69.277
67.360
65.565
65.481
58.878
57.176
57.076
32.928
32.338
30.106
30.007
29.968
29.853
29.761
29.700
29.669
25.321
23.051
14.248
X : parts per Million : 13C

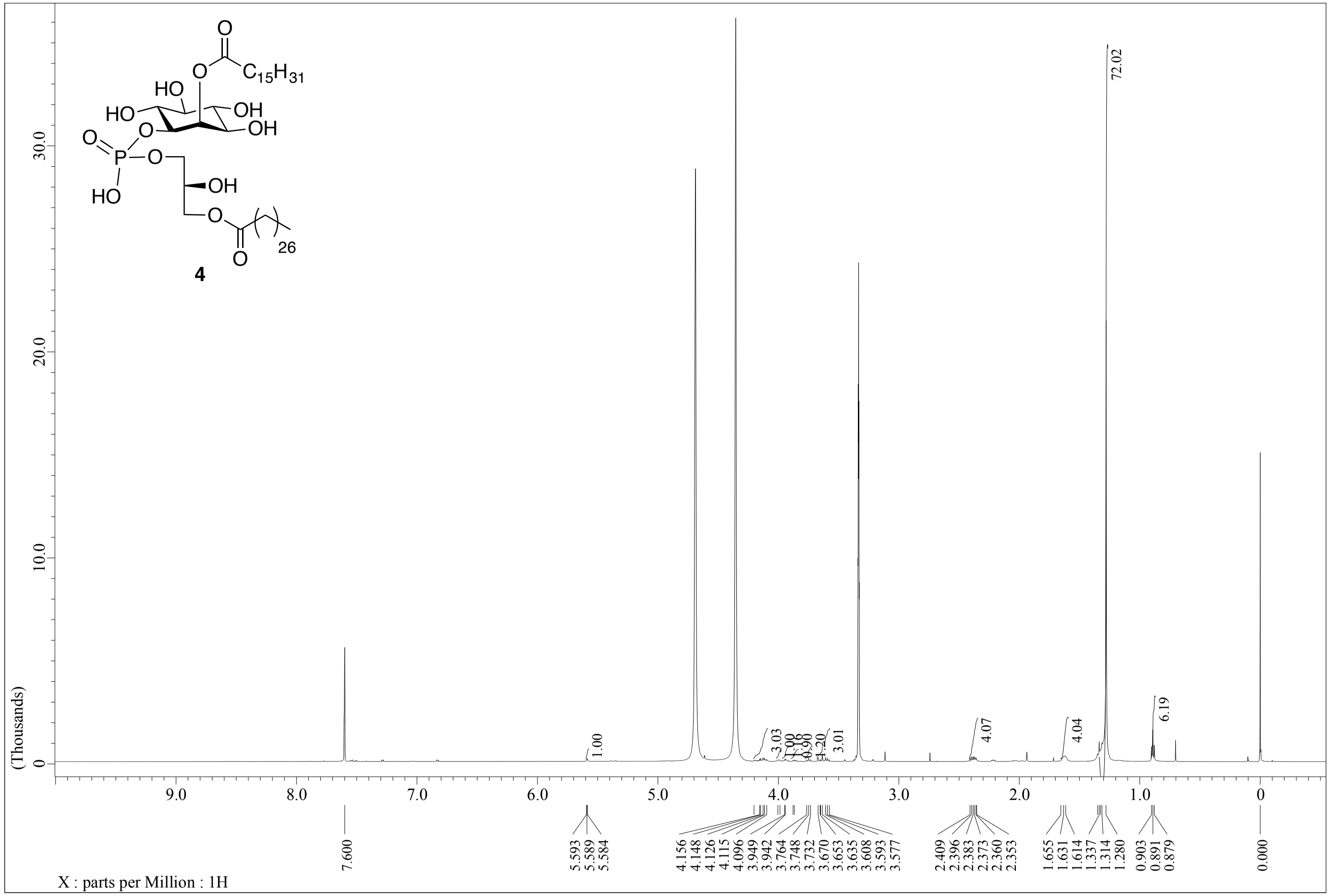

4
(Thousands)
30.0
20.0
10.0
0
9.0
8.0
7.0
6.0
5.0
4.0
3.0
2.0
1.0
0
X : parts per Million : 1H
7.600
5.593
5.589
5.584
4.156
4.148
4.126
4.115
4.096
3.949
3.942
3.764
3.748
3.732
3.670
3.653
3.635
3.608
3.593
3.577
2.409
2.396
2.383
2.373
2.360
2.353
1.655
1.631
1.614
1.337
1.314
1.280
0.903
0.891
0.879
0.000
1.00
3.03
3.01
4.07
4.04
72.02
6.19

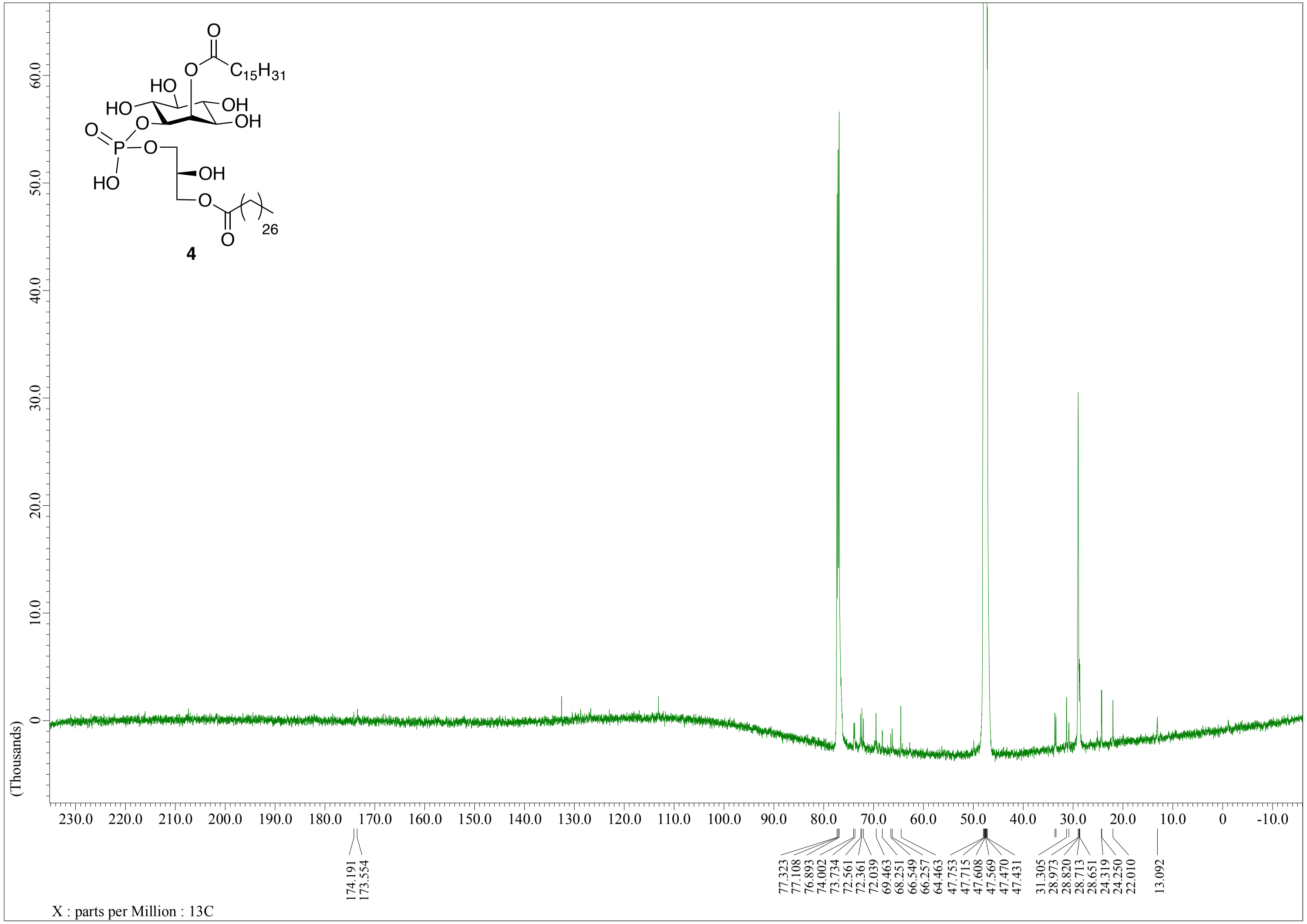

4
(Thousands)
X : parts per Million : 13C
174.191
173.554
77.323
77.108
76.893
74.002
73.734
72.561
72.361
72.039
69.463
68.251
66.549
66.257
64.463
47.753
47.715
47.608
47.569
47.470
47.431
31.305
28.973
28.820
28.713
28.651
24.319
24.250
22.010
13.092

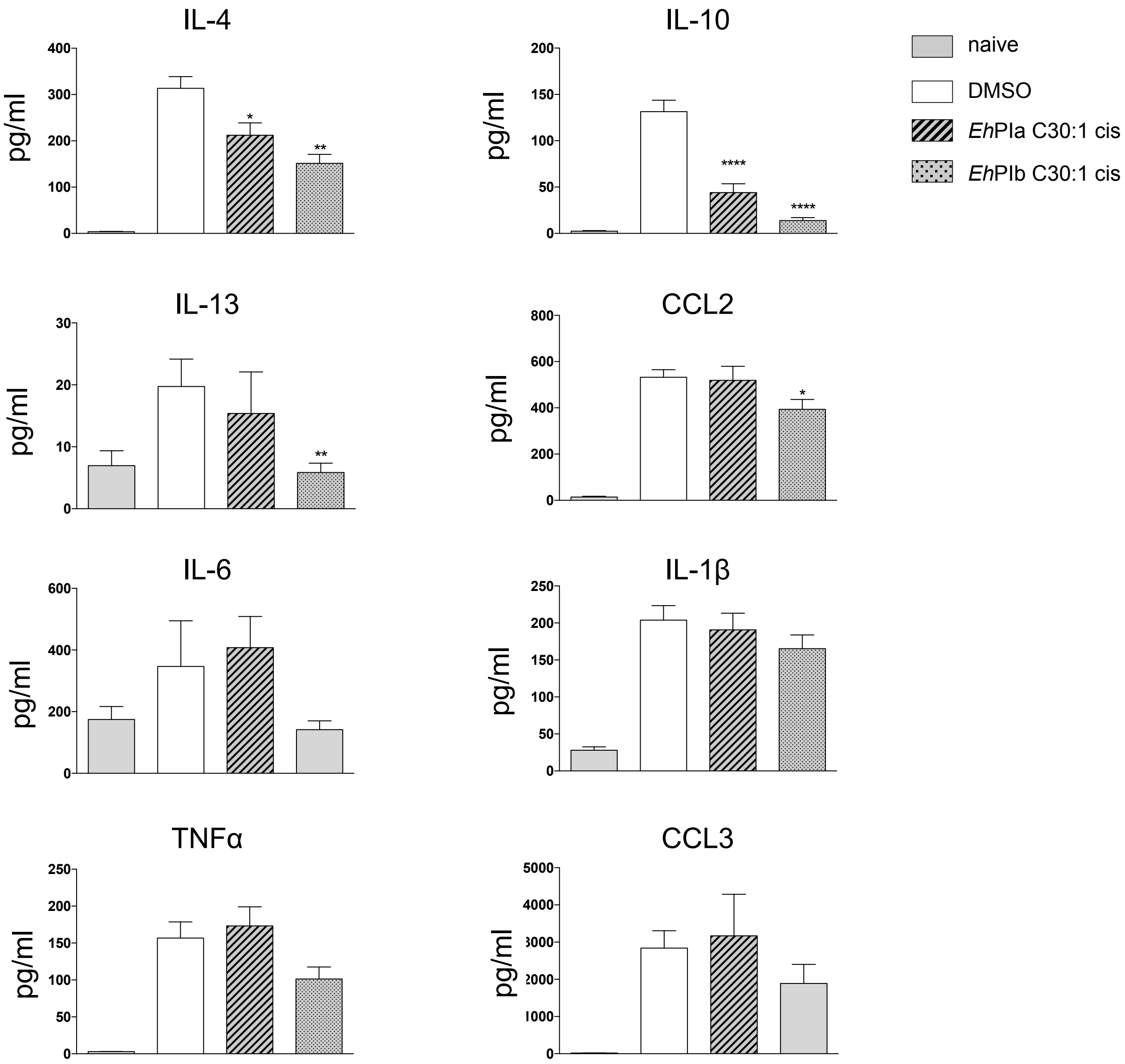

Supplementary Fig 1. Cytokine and chemokine profiles in tissue lysates derived from the footpads of *L. major*-infected and treated BALB/c mice. Cytokine and chemokine concentrations in the supernatants of tissue lysates from *L. major*-infected female BALB/c mice treated with DMSO (diluted 1:10/25 µl) or *Eh*PIa or *Eh*PIb C30:1 cis (5 µg in PBS/25 µl) as determined in a cytometric bead assay (LEGENDplex™, BioLegend). Data are expressed as the mean ± SEM of n=4 for each group. *p<0.05; **p<0.01; and ***p<0.001 (unpaired Student's t test).
